# Photocatalytic CO_2_ reduction with aminoanthraquinone organic dyes

**DOI:** 10.1038/s41467-023-36784-7

**Published:** 2023-02-25

**Authors:** Qinqin Lei, Huiqing Yuan, Jiehao Du, Mei Ming, Shuang Yang, Ya Chen, Jingxiang Lei, Zhiji Han

**Affiliations:** MOE Key Laboratory of Bioinorganic and Synthetic Chemistry, School of Chemistry, Sun Yat-sen University, Guangzhou, 510275 China

**Keywords:** Light harvesting, Photocatalysis, Energy, Homogeneous catalysis

## Abstract

The direct utilization of solar energy to convert CO_2_ into renewable chemicals remains a challenge. One essential difficulty is the development of efficient and inexpensive light-absorbers. Here we show a series of aminoanthraquinone organic dyes to promote the efficiency for visible light-driven CO_2_ reduction to CO when coupled with an Fe porphyrin catalyst. Importantly, high turnover numbers can be obtained for both the photosensitizer and the catalyst, which has not been achieved in current light-driven systems. Structure-function study performed with substituents having distinct electronic effects reveals that the built-in donor-acceptor property of the photosensitizer significantly promotes the photocatalytic activity. We anticipate this study gives insight into the continued development of advanced photocatalysts for solar energy conversion.

## Abstract (web summary)

A class of inexpensive aminoanthraquinone organic dyes are shown to facilitate visible-light-driven CO_2_ reduction. Overall reaction efficiencies were found to be optimal when both electron donating and accepting groups were on a single dye molecule.

## Introduction

Light-driven reduction of CO_2_ into value-added chemicals represents a sustainable way for the direct utilization of solar energy and conversion of greenhouse gas^1–3^. In an artificial photosynthetic (AP) scheme, a photosensitizer (PS) harvests the sunlight and transfers the energetic electron to a catalytic center which reduces CO_2_^4–6^. In the past decades, both homogeneous and heterogeneous AP systems have been investigated extensively for photocatalytic CO_2_ reduction^7,8^. However, the activity of current systems is still low for practical use. A frequent challenge is the development of highly active PSs that promote light-driven redox reactions. For the rational design of catalytic systems, molecular approaches have shown great advantages in unraveling factors that govern photocatalytic reactions. In this context, we report here a class of highly active organic PSs in precious metal-free systems for photocatalytic CO_2_ reduction.

Noble-metal-based PSs have demonstrated high activity in photocatalytic CO_2_ reduction^6,9–19^. Their long-lived excited states facilitate electron transfer from the excited state of the PS (PS*) to the catalyst in an oxidative quenching pathway. To provide a potentially widespread implementation, accelerating progress has been made in the development of inexpensive PSs to perform the same catalytic reaction^20^. Recently, PSs based on first-row transition metals such as Cu^21–24^ and Zn^25^ have been studied for light**-**driven CO_2_ reduction, with turnover numbers (TONs) of 40–1566.

Due to being readily available in nature and because they are synthetically easy to functionalize, organic PSs are promising alternative light-absorbers for photocatalytic CO_2_ reduction^26–28^. However, there are only a few reported organic PSs functioning in the visible region, and these systems usually have to perform with high PS concentrations due to their relatively low activity^29–43^. For example, 9-cyanoanthracene has been reported to give a turnover frequency (TOF) of ~0.015 h^−1^ (vs. PS) in a noble-metal-free system using Fe–tetraphenylporphyrin as the catalyst (TOF_Fe_ ~1.5 h^−1^)^29^. Acriflavine was found to exhibit a TON_PS_ of 5, when employed with a Co dipyridyl catalyst^30^. Purpurin, reported by Lau, Robert, and Chen groups, has shown activity for the reduction of CO_2_ to CO with a series of Co, Fe, Ni polypyridyl, and Fe porphyrin catalysts^31–33^, achieving an optimal TON_PS_ of 1300^31^. Later, Robert et al. found that a phenoxazine-based organic PS promotes the reduction of CO_2_ to CO and CH_4_ with a total TON_PS_ of ~2 in 102 h^34^. 2,4,5,6-tetrakis(carbazole-9-yl)-1,3-dicyanobenzene studied by Chao et al. gives a TON_PS_ up to 1196 in CO generation using *mono*– and *bis*–terpydrine Fe catalysts^35–37^. Eosin Y, which was originally reported in photocatalytic H_2_ production by the Eisenberg group^38–40^, is also an active PS for CO_2_ reduction to formate when using Ni pyridylthiolate catalysts, although it performs with considerably lower activity (TON_PS_ = 28) than that of the catalyst (TON_Ni_ = 14,000)^41^. Because achieving high activity for both the PS and the catalyst would be beneficial for developing versatile photocatalytic systems and applications in other relevant studies such as photoelectrochemical and supramolecular photocatalytic systems, this difficulty has led to assembling complicated molecular architectures with precious metals^44–49^.

**Fig. 1:**
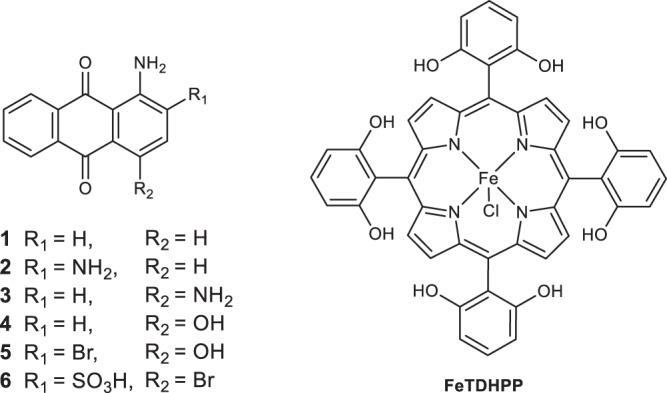
Structure diagram. Structures of PSs **1**–**6** and FeTDHPP in the study.

Several methods have been studied for improving the photocatalytic activity of organic PSs^28^. For example, the introduction of a heavy atom (such as Br or I) or a heteroatom (such as S or Se) to the xanthene-based dye has been found to facilitate intersystem crossing to generate a longer-lived ^3^ππ* state, leading to improved activity in photocatalytic H_2_ production^38,50^. Tuning the electron donors and acceptors in organic dyes help increasing the power conversion efficiencies of dye-sensitized solar cells^51,52^. We recently demonstrated that the coordination of polyhydroxy-anthraquinones to a redox active Cu center effectively enhanced the photocatalytic activity in both proton and CO_2_ reductions^22,53^. In the present study, we report the application of simple yet more active aminoanthraquinone organic PSs **1**–**6** (Fig. 1) for visible light-driven reduction of CO_2_ to CO. Different from previous systems, high TONs for both the PS and the catalyst can be realized. In addition, the systems contain no precious metal and use commercially available organic PSs. The photochemical steps and mechanism for CO_2_ reduction have been studied in detail. Our structure-function study shows that the donor–*π*–acceptor design of the anthraquinone (AQ) unit through controlling electron substituents facilitates faster reductive quenching of the PS* and results in a much higher catalytic rate for CO_2_ reduction.

## Results and discussion

### Absorption, emission, and electrochemistry of PSs

**Fig. 2:**
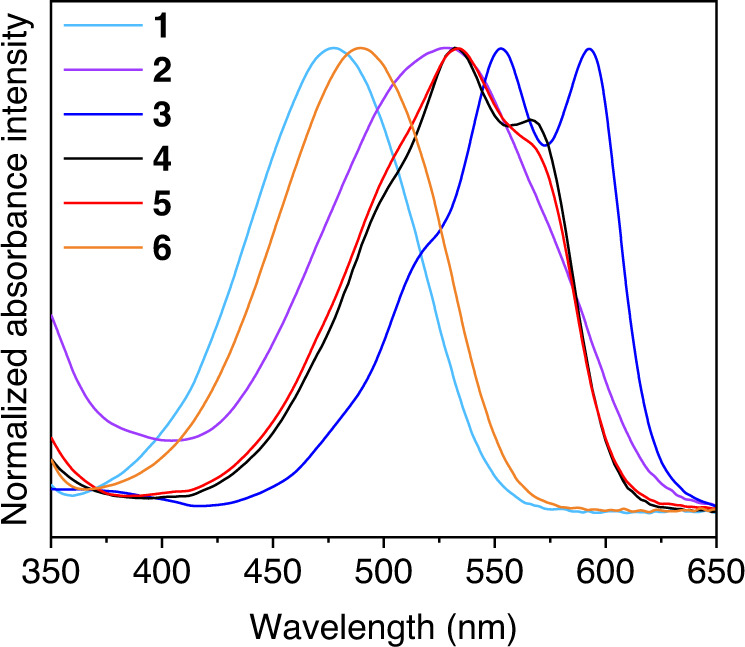
UV–vis spectra. Normalized absorption spectra of PSs **1**–**6** in DMF. Source data are provided as a Source Data file.

**1**–**6** display strong electronic absorption across the visible region in dimethylformamide (DMF). The maximum absorption bands ranging from 478 to 592 nm can be largely tuned by altering the substituents on the anthraquinone moiety (Fig. 2). The molar absorption coefficients (0.68–1.58 × 10^4^ M^−1^ cm^−1^) were calculated from the linear relationship between the absorbance and the concentration (Table 1 and Supplementary Figs. 1–6). Upon irradiation with 365 nm light, these PSs produce intense red fluorescence at the 600–700 nm region with lifetimes (*τ*_0_) of 0.66–1.02 nanoseconds (Table 1 and Supplementary Fig. 7).

**Table 1:** Photophysical, electrochemical, and photocatalytic CO_2_ reduction data of PSs **1**–**6**

PS	*λ*_max_ abs/nm (ε M^−1^ cm^−1^)	*λ*_max_ em (nm)	*E*_red_ (V vs. SCE)	TON_Fe_^a^	TOF_Fe_^a^	Sel_CO_^a^ (%)	CO^b^ (μmol)	TON_PS_^b^	*Φ*^c^ (%)	*Φ*_FL_ (%)	*τ*_0_^d^ (ns)	*k*_q_^f^ (M^−1^ s^−1^)
**1**	478 (6790)	600	−0.96, −1.59	2395 ± 228	1510 ± 104	99.6 ± 0.1	50 ± 6	2011 ± 257	8.9 ± 0.8	4.7	0.88 ± 0.013	3.9 × 10^9^
**2**	528 (8940)	650	−1.10, −1.70	2738 ± 190	69 ± 8	99.5 ± 0.2	12 ± 2	482 ± 76	0.3 ± 0.04	2.3	0.72 ± 0.004	2.1 × 10^9^
**3**	592 (15,810)	662	−1.15, −1.64	3551 ± 501	593 ± 24	99.3 ± 0.2	38 ± 3	1523 ± 126	3.0 ± 0.1	5.3	0.82 ± 0.003	2.7 × 10^9^
**4**	532 (12,040)	620	−0.84, −1.44	8360 ± 449	1614 ± 112	99.6 ± 0.1	71 ± 4	2849 ± 161	8.1 ± 0.3	7.1	0.94 ± 0.008	5.2 × 10^9^
**5**	534 (9170)	635	−0.68, −1.19	21616 ± 2351	4028 ± 669	>99.9	153 ± 10	6012 ± 606	11.1 ± 0.9	7.0	1.02^e^ ± 0.005	7.5 × 10^9^
**6**	490 (7460)	607	−0.86, −1.30	907 ± 154	93 ± 17	98.5 ± 0.6	30 ± 2	1183 ± 78	2.0 ± 0.3	2.6	0.66 ± 0.002	1.6 × 10^9^

Error bars denote standard deviations, based on at least three separated runs. Source data are provided as a Source Data file.

^a^60 mM BIH, 0.6 μM FeTDHPP, and 20 μM PS, *λ* > 400 nm, TON and Sel_CO_ calculated in 48 h, TOF calculated in 0.5 h for PS **1**, 2 h for PS **2**, and 1 h for PSs **3**–**6**.

^b^60 mM BIH, 20 μM FeTDHPP and 5 μM PS, *λ* > 400 nm, amount of CO and TON_PS_ calculated in 72 h.

^c^60 mM BIH, 20 μM FeTDHPP and 20 μM PS, *λ* = 450 nm, *Φ* calculated in 1 h.

^d^50 μM PS, a picosecond pulsed diode laser (*λ* = 472 nm) was used as the excitation source.

^e^Under CO_2_. The *λ*_max_em of each photosensitizer is selected as the emission wavelength.

^f^*k*_q_ calculated from linear fitting of the Stern–Volmer plot based on average values of three sets of data.

To further examine the organic dyes as potential PSs for photocatalytic reactions, electrochemical measurements were conducted (Table 1). Cyclic voltammograms (CVs) of **1**–**6** show two reduction events (Supplementary Fig. 9), with both reversible waves for **1**–**5**, whereas for PS **6**, a reversible and a second quasi-reversible reduction peaks were observed. The exact reduction potentials were measured by square wave voltammetry (SWV) (Table 1 and Supplementary Fig. 10). The photophysical and redox properties of the aminoanthraquinone dyes (Table 1 and Supplementary Table 1) thus suggest they serve as promising PSs for photocatalytic CO_2_ reduction.

### Photocatalytic CO_2_ reduction

The activity of CO_2_ reduction by PSs **1**–**6** was studied in CO_2_-saturated DMF solutions under irradiation with a white light-emitting diode (LED, *λ* > 400 nm, 100 mW/cm^2^). FeTDHPP (Fig. 1) was used as the CO_2_ reduction catalyst, for the reason that it has been demonstrated to provide high activity in photocatalytic systems from our previous study^22^. 1,3-dimethyl-2-phenyl-2,3-dihydro-1*H*-benzo[*d*]imidazole (BIH) was used as the sacrificial donor to replace the oxidative half-reaction in the AP scheme. The gaseous products in the headspace were quantified in real time by gas chromatography (GC) equipped with a thermal conductivity detector (TCD) and a flame ionization detector (FID).

**Fig. 3:**
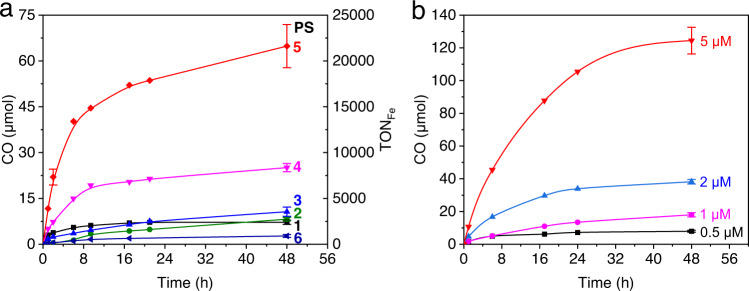
Photocatalytic CO_2_ reduction. **a** Systems containing 20 µM **1**–**6**, 0.6 µM FeTDHPP, and 60 mM BIH; **b** systems containing 60 mM BIH, the same concentrations (0.5, 1, 2, 5 µM) of **5** and FeTDHPP. Irradiating conditions: CO_2_ atmosphere, 5.0 mL CO_2_-saturated DMF, 20 °C, white LED (*λ* > 400 nm, 100 mW/cm^2^), 6.33 cm^2^ light contact surface area. Error bars denote standard deviations, based on at least three separated runs. Source data are provided as a Source Data file.

Figure 3a and Table 1 display the photocatalytic results of **1**–**6** under the same conditions (20 μM PS, 0.6 μM FeTDHPP, 60 mM BIH). The yield rate of CO is shown in Supplementary Table 2. In the series of experiments, CO is observed as the major product and the production of H_2_ is significantly suppressed. PSs **1**–**5**, with amino and hydroxyl substituents on the AQ, exhibit generally high selectivity of CO (>99%), whereas PS **6** which contains a strongly acidic sulfonyl group gives a slightly lower selectivity of 98.5% ± 0.6%. Importantly, varying the functional groups on the AQ ring results in very different TONs of CO. The systems with the amino-substituted AQs (**1**–**3**) show TON_Fe_ of 2395–3551 in 48 h. Under the same conditions but with an OH-substituted aminoanthraquinone (**4**), a considerably higher TON of 8360 ± 449 was obtained. The activity of the system can be further improved by including a heavy atom Br as the substituent at the 2–position (**5**), achieving a TON of 21,616 ± 2351 and a TOF of 4028 ± 669 mole CO/h per mole of catalyst. However, changing the OH group to a sulfonyl one (**6**) markedly decreases the light-driven activity (TON = 907 ± 154).

To investigate the optimal activity of the PS, photocatalytic experiments were performed at high concentrations of FeTDHPP (20 μM) and BIH (60 mM) (Table 1), where the activity is controlled by the [PS] (Supplementary Figs. 11–13). The PS **1** shows a TON_CO_ (vs. PS) of 2011 ± 257 in 72 h. The NH_2_- or SO_3_H- substituted ones result in decrease in activity (TON = 482 ± 76 for **2**, 1523 ± 126 for **3**, 1183 ± 78 for **6**). Consistent with the results described above, the PSs **4** and **5** give much higher activity in the series, with TONs of 2849 ± 161 and 6012 ± 606, respectively.

The high activity of the PS and the catalyst, although obtained at different catalytic conditions, suggests that it may be possible to realize high activity for both the PS and the catalyst in one photocatalytic system. Indeed, when the experiment was performed under the same concentration of **5** and FeTDHPP (Fig. 3b and Supplementary Table 3), the system achieves a TON as high as 4978 ± 326 and a quantum efficiency of 11.1% ± 0.9% at 450 nm (based on two photons per CO), underscoring that both the light-harvesting and the CO_2_ reducing processes are efficient in catalysis. The exceptional light-driven activity of the study is in contrast to those reported for other noble metal-free systems which usually show very different activity for the PS and the catalyst (Supplementary Table 4).

To study factors that influenced the light-driven stability, each component was added to the system when the rate of CO production was substantially decreased (Supplementary Fig. 14). Although BIH was nearly consumed in the conditions, addition of BIH to the system did not resume the activity (Supplementary Fig. 14a), which suggests decomposition of either the PS or the catalyst. When a mixture of PS and BIH was added, only ~5% activity was recovered (Supplementary Fig. 14d), indicating that most of the catalyst has decomposed. Indeed, with the addition of catalyst and BIH, CO production continued with a ~50% increase (Supplementary Fig. 14f). However, even though all three components were added, a similar level (~60%) of recovery was observed (Supplementary Fig. 14g), which is presumably due to light competition between the decomposed species and the added PS. These results thus indicate that the Fe porphyrin catalyst undergoes a faster decomposition than the anthraquinone dye in the catalytic system.

The nature of the system was investigated. To confirm the homogeneity of the system, dynamic light scattering (DLS) and Hg-poisoning experiments were conducted. DLS results indicate there is no presence of nanoparticles in the pre- and post- catalytic systems (Supplementary Fig. 15). An excess amount of metallic Hg^0^ in the system does not result in a significant change of the activity (Supplementary Fig. 16), which rules out the possibility that the activity of CO_2_ reduction is contributed from amalgam-forming metal impurities. In addition, isotopic labeling experiments performed under an atmosphere of ^13^CO_2_ show exclusive ^13^CO as the product (Supplementary Fig. 17). These pieces of evidence are consistent with a homogeneous CO_2_ reduction system in the study.

### Mechanism of CO_2_ reduction

**Fig. 4:**
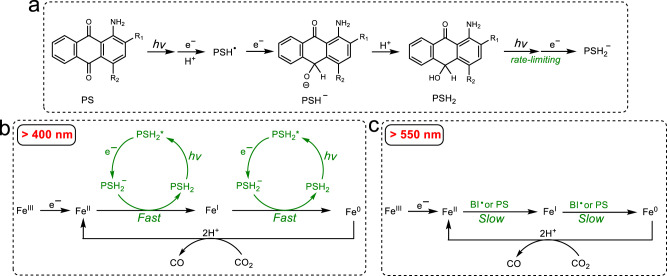
Proposed mechanism of light-driven CO_2_ reduction. **a** Light-driven redox steps of the PS; **b** CO_2_ reduction mechanism under >400 nm light irradiation; **c** CO_2_ reduction mechanism under >550 nm light irradiation.

In a multi-component system, reductive quenching (electron transfer from the electron donor to the PS*) and oxidative quenching (electron transfer from the PS* to the catalyst) are two major photochemical pathways affecting the overall light-driven catalytic rate. Because aminoanthraquinone undergoes excited-state intramolecular proton transfer (ESIPT)^54^, changing proton concentration may affect the fluorescence spectra during quenching experiments. In fact, we observed that the addition of BIH (which is slightly basic) to PSs **4** and **5** resulted in uneven quenching of the fluorescence at different wavelengths and that the fluorescence quenching rate constants (*k*_q_) were calculated to be higher than the diffusion-controlled limit in DMF (Supplementary Figs. 18 and 19). Since the fluorescence lifetimes of the ESIPT tautomers have been reported to be identical^54^, we determined the *k*_q_ values by measuring the change of fluorescence lifetime in the presence of BIH. The reductive fluorescence quenching of **1**–**6** was found to be fast near the diffusion-controlled limit (>10^9^ M^−1^ s^−1^) (Table 1 and Supplementary Fig. 20). Because of significant overlap in both the absorption and the emission spectra of the PSs and the Fe catalyst (Supplementary Fig. 21), the oxidative quenching rate constants could not be obtained accurately. Based on the UV–vis and ^1^H NMR spectra (Supplementary Figs. 22–29), there is no reaction between the PS and quenchers (BIH and FeTDHPP) at their ground states, which rules out a static quenching pathway. These results along with the fact that a much higher concentration of BIH (>3000 times) than that of the FeTDHPP in CO_2_ reduction, suggests the system proceeds with a reductive quenching pathway (Fig. 4). However, since the triplet quantum yields of PSs **1** and **3** have been reported to be 3% and <0.1% in methanol respectively^55^, it should be noted that reductive quenching occurring through ^3^PS^*^ is another plausible photochemical pathway, especially for the heavy-atom containing compound **5**. In fact, reductive quenching has been reported as the most common mechanism in other organic dye-containing systems^28,29,31,34,37^. Thus, the reductive rate and the reduction power of the reduced PS are two critical factors influencing the light-driven electron-transfer process in such photocatalytic reactions.

**Fig. 5:**
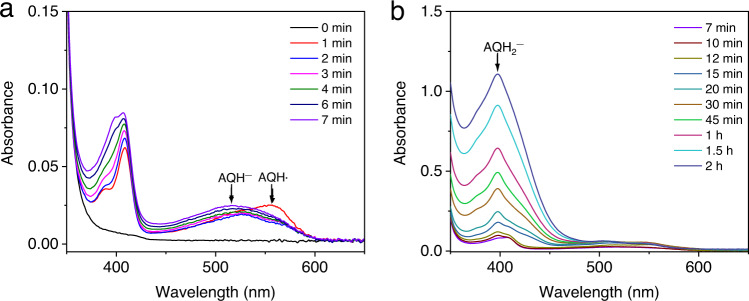
UV–vis absorption spectra of AQ. A system containing 0.02 mM AQ, 3.0 mM BIH, and 2 mL DMF in a quartz cuvette (10-mm path length) under N_2_ upon irradiation with white LED light (*λ* > 400 nm, 100 mW/cm^2^) at 20 °C. Irradiation time ranges from 0 to 7 min (**a**), and from 7 min to 2 h (**b**). Source data are provided as a Source Data file.

The light-driven redox process of the AQ-based dyes has been a subject of great interest in photochemistry^56–60^, however, due to the various intermediates in reductions and protonations, the reaction mechanism is still under debate. Because AQ generates a similar intermediate at ~400 nm as PSs **1**–**6** during photolysis (Supplementary Figs. 30–36), the light-driven process of AQ was investigated to gain useful mechanistic information. UV–vis spectra reveal that photolysis of AQ in the presence of BIH in DMF quickly generates a species at 560 nm in 1 min (Fig. 5a). Since it is clear neither the 1 electron reduced AQ (545 nm) nor the 2 electron reduction product AQ^2−^ (622 nm) gives a good match^56^, this species is assigned to an e^−^/H^+^ product AQH•. In fact, a semiquinone at 570 nm has been detected in an osmium triad^61,62^. An AQ^−^ species can be observed unambiguously from a reaction with NaBH_4_ (Supplementary Fig. 37). It is not surprising that the AQ^−^ ^56^ undergoes a fast protonation by BIH^+^ to produce the AQH•. Upon continued irradiation, an intermediate at ~520 nm is observed (Fig. 5a), which is consistent with the generation of an AQH^−^^56^. A further protonation of the AQH^−^ species to generate a 10-hydroxyanthrone (AQH_2_) is expected to take place based on ^1^H NMR spectra (Supplementary Fig. 38). The UV–vis spectra also shows another absorption peak at ~407 nm (Fig. 5a), which is similar to a proposed AQH_2_ intermediate at 407 nm from an AQ-containing pentad complex reported by Wenger et al.^59^.

Robert et al. have shown that CO_2_ reduction by FeTDHPP occurs at an Fe(0) oxidation state at −1.55 V vs. SCE^16,63,64^. However, our electrochemical studies reveal much positive reduction potentials for the PS^−^ and PS^2−^ species (Table 1), indicating that electron-transfer from these reduced species including their protonated forms PSH and PSH_2_ (presumably with more positive potentials)^65,66^ to the catalyst is unfavorable. The generation of the Fe(0) must arise from a more reduced PS. Indeed, the AQH_2_ species generated from subsequent reductions and protonations gives a relatively long-lived (17.3 ns) fluorescence (Supplementary Figs. 39 and 40), which can be quickly quenched by BIH (Supplementary Fig. 41). Moreover, the AQH_2_ moiety in a pentad system has been reported to be highly fluorescent with a long lifetime of 4.7 μs^59^. Thus, a plausible photochemical pathway involves reduction of the Fe catalyst by a PSH_2_^−^ photoproduct, which can be generated from excitation of the PSH_2_ followed by reductive quenching (Fig. 4).

The intermediate generated from AQ at ~400 nm starts to appear at 15 min and continue to increase in 2 h during photolysis (Fig. 5b), which is commonly observed with PSs **1**–**6** during CO_2_ reduction (Supplementary Figs. 31–36). Although this intermediate exhibits a similar absorption feature as the AQH_2_, a much slower generation of the species than that for the AQH• suggests it is not an AQH_2_. Furthermore, we observed fast generation of CO and an Fe(I) species by adding 0.25 equiv of FeTDHPP (with respect to PS) to the 406 nm species generated from light irradiation of a mixture of **5** and BIH (Supplementary Fig. 42). In the experiments, an average of 0.34 equiv of CO (vs. PS) was obtained, which is close to the theoretical maximum yield (0.33 equiv) based on the proposed mechanism in Fig. 4. In a control experiment before generating the 406 nm species, no CO was detected (Supplementary Fig. 43). Based on these results, this photoproduct at ~400 nm is tentatively assigned to a PSH_2_^−^.

To examine the reduction power of PSH_2_^−^, in situ electrochemical measurements were conducted for the light-driven systems. SWV experiments with the photochemically generated species at ~400 nm show the appearance of new reduction waves at potentials more negative than −1.90 V vs. SCE for PSs **1**–**6** (Supplementary Fig. 44). Hence, electron-transfer from the PSH_2_^−^ to the FeTDHPP that leads to production of the required Fe(0) intermediate for CO_2_ reduction is thermodynamically feasible.

**Fig. 6:**
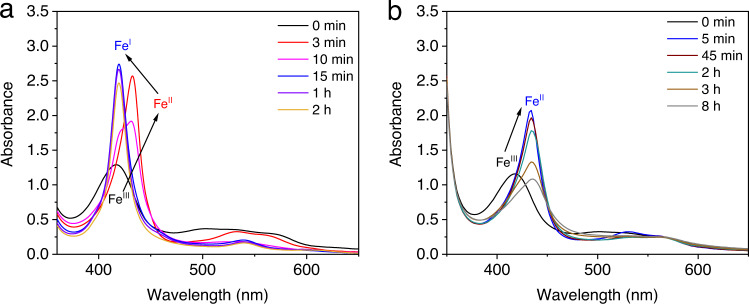
UV–vis spectra investigating the reduction of FeTDHPP. Systems containing 30 mM BIH, 20 μM FeTDHPP, 20 μM **5**, and 2 mL DMF in a quartz cuvette (10-mm path length) under N_2_ at 20 °C, upon irradiation with (**a**) white LED light (*λ* > 400 nm, 100 mW/cm^2^), or with (**b**) a 300 W xenon light source equipped with a 550 nm cut-off filter (*λ* > 550 nm) for 8 h. Source data are provided as a Source Data file.

Additional experiments were conducted to investigate reductions of the Fe catalyst. In the photocatalytic experiments with white LED (*λ* > 400 nm), UV–vis spectra suggest that the Fe(III) compound (416 nm) is completely converted to an Fe(II) species (432 nm) within 3 min and then to an Fe(I) species (420 nm) which continues to decrease during CO_2_ reduction (Fig. 6a and Supplementary Fig. 45). This observation is consistent with a previously reported mechanism by Robert et al.^4,67^. Because both the PSH_2_^−^ and the BI• (−1.60 V vs. SCE in DMF)^22^ are potential reductants in generating the Fe(I) and Fe(0) intermediates, it is crucial to understand the role of BIH in the system. In a photocatalytic experiment with 10 mM [BIH], the total amount of CO generated is near the theoretical maximum yield of BIH (Supplementary Fig. 46), which indicates BIH donates two electrons in CO_2_ reduction. The first electron-transfer process is usually from BIH to the excited state of PS, which has been well-studied^68^. However, the actual mechanism by which the second electron of the sacrificial donor transfers from the significantly more reducing BI• to either the excited PS, or an Fe(II), or an Fe(I), remains uncertain.

To study this further, photocatalytic CO_2_ reduction was conducted under a xenon light source equipped with a 550 nm cut-off filter to shut down the pathway involving the PSH_2_^−^. In this case, there is no PSH_2_^−^ observed from the UV–vis spectra (Supplementary Fig. 47), and CO production is considerably slower (TON = 6 in 5 h). The major catalytic species detected corresponds to the Fe(II), while the Fe(I) intermediate is present in a notably less amount compared with that generated under the *λ* > 400 nm light (Fig. 6b and Supplementary Figs. 47 and 48). Similar results are observed in experiments performed under 450 nm and 525 nm LED light with similar photon numbers (Supplementary Figs. 49 and 50). Meanwhile, the production of CO under 525 nm LED light (TON = 1.4 in 8 h) is also lower than that under the 450 nm LED light (TON = 344 in 8 h). Furthermore, irradiating a mixture of BIH and FeTDHPP gave almost no Fe(I) and only the Fe(II) was detected (Supplementary Fig. 51). These results are all consistent with the fact that although the BI• can reduce Fe(II) and Fe(I) to the Fe(0) intermediate, these are much slower processes compared with the ones using PSH_2_^−^ as the reductant. Thus, BI• is likely responsible for the reduction of PSH_2_* in a photochemical step, while the resulting PSH_2_^−^ proceeds in reducing the Fe(II) or Fe(I) (Fig. 4).

**Fig. 7:**
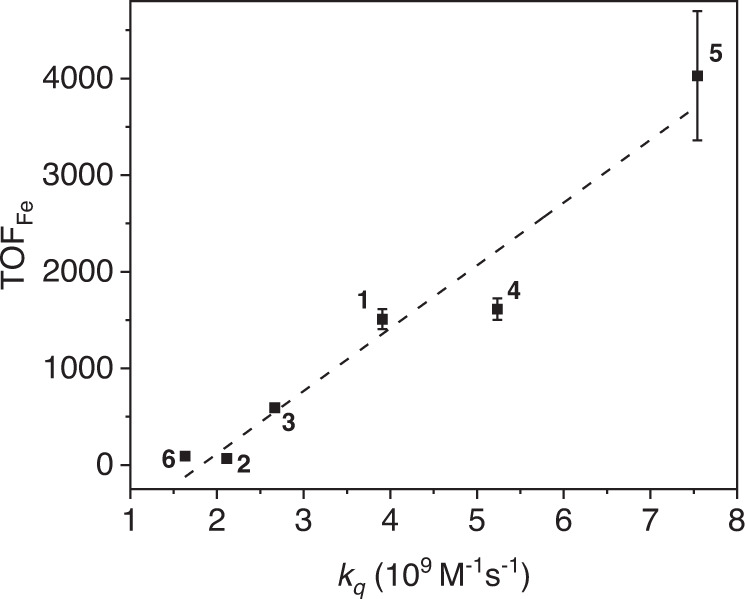
Relationship of TOF and *k*_q_. Plot of the initial rates of CO generation with respect to the reductive quenching rate constants of PSs **1**–**6**. The dotted line is shown for viewing convenience. Error bars denote standard deviations, based on at least three separated runs. Source data are provided as a Source Data file.

The observation of the PSH_2_^−^ species by UV–vis during CO production suggests that the formation of PSH_2_^−^ may be rate-limiting in catalysis. Interestingly, we found that the TOF of CO production and the first reductive quenching *k*_q_ of PSs **1**–**6** follows a generally linear trend, in which a faster quenching rate is observed with a higher TOF (Fig. 7). However, further evidence is necessary to identify the substituent effect on the photo-conversion of PSH_2_ to PSH_2_^−^ in CO_2_ reduction. In addition, no CO was detected from a system using AQ as the PS although an AQH_2_^−^ species was observed (Supplementary Fig. 30), which indicates the functional groups of **1**–**6** play important roles in promoting the generation of the PSH_2_^−^. Based on the electrochemical data of **1**–**6** and 1-amino-2-methylanthraquinone (Table 1 and Supplementary Figs. 9 and 10), the −NH_2_ group acts as an electron-donating group while the −OH group is electron-withdrawing on AQ. These distinct electronic groups may essentially create an internal donor-acceptor property by de-symmetrizing the organic molecule, which facilitates electron-transfer of the dye. Consistent with the proposal, introducing –NH_2_ to the 1–position of AQ (PS **1**) greatly promotes photocatalytic CO_2_ reduction, while having an additional –NH_2_ group at the 4–position (PS **3**) results in a decrease of activity. Furthermore, the higher activity of **4** and **5** may be attributed to the reverse electronic effects of the –OH and –Br groups as compared to the –NH_2_. For PS **6**, although sulfonyl is also electron-withdrawing, its activity of CO_2_ reduction is considerably lower than that of **1**–**5**. This is presumably due to that the acidic sulfonyl group promotes the transfer of proton to the catalyst thus enhancing H_2_ generation. Indeed, the selectivity for H_2_ of **6** is the highest in our study (Table 1). Although this selectivity (1.5%) is low compared with that of CO (98.5%), the intermediates generated from the H_2_ pathway may significantly alter the reaction mechanism by introducing extra energy barriers in CO_2_ reduction^4^.

In summary, this paper describes the application of a series of simple organic light absorbers, based on naturally abundant anthraquinone dyes, in promoting visible light-driven CO_2_ reduction. Unlike previously reported systems, high activity for both the PS and the catalyst has been demonstrated in our study. The mechanistic study suggests that the hydroxyanthrone forms of PS (PSH_2_ and PSH_2_^−^), generated from reductive quenching, are important intermediates in the light-driven catalytic steps. The most active PS was found by employing both electron-donating and withdrawing groups on the anthraquinone. Thus, this work presents a class of inexpensive dyes to access high activity in CO_2_ reduction and provides understanding for improving other light-driven and light-electricity-driven systems for practical applications, such as water splitting, solar cell, and organic synthesis.

## Methods

### Materials

Compound **1** (97%) and compound **3** (>97%) were purchased from Aladdin. Compound **2** was purchased from Sigma-Aldrich. Compound **4** (96%) was purchased from Alfa Aesar. Compound **5** (97%) was purchased from BIDE. Compound **6** (98%) was purchased from Macklin. 1-Amino-2-methylanthraquinone (>90%) was purchased from Shanghai Xian Ding Biotechnology Co. Ltd. (Shanghai, China). Compound **1** was recrystallized twice from hot acetone. Compound **4** was recrystallized twice from a mixture of hot acetone and acetonitrile. 1-Amino-2-methylanthraquinone was recrystallized twice from hot ethanol until it is pure according to the ^1^H NMR spectrum. Other solvents and chemicals are commercially purchased and used as obtained without further purification. BIH was prepared based on a method from the literature^69^.

### Synthesis of FeTDHPP

FeTDHPP was prepared from a modified method of the literature^64^. A solution of 2′,6′-dimethoxybenzaldehyde (1.0 g, 6.02 mmol) and pyrrole (0.419 mL, 602 mmol) in CHCl_3_ (600 mL) was degassed by N_2_ for at least 20 min. BF_3_·OEt_2_ (0.228 mL, 0.87 mmol) was added drop by drop via a syringe. After the solution was stirred at room temperature under N_2_ in the dark for 1.5 h, 2,3-dichloro-5,6-dicyano-1,4-benzoquinone (DDQ) (1.02 g, 4.51 mmol) was added. The mixture was stirred for an additional 1.5 h at reflux. After cooling to room temperature, the mixture was added with 1 mL of triethylamine to neutralize the excessive acid. Then the solvent was removed, and the resulting black solid was purified by column chromatography (silica gel, CH_2_Cl_2_) affording 5, 10, 15, 20-tetrakis(2′, 6′-dimethoxyphenyl)-21H,23H-porphyrin as a purple powder (290 mg, 23%). ^1^H NMR (400 MHz, CDCl_3_): *δ* 8.67 (s, 8H), 7.68 (t, *J* = 8.4 Hz, 4H), 6.98 (d, *J* = 8.4 Hz, 8H), 3.50 (s, 24H), −2.50 (s, 2H). HRMS (*m*/*z*): [M + H]^+^ calcd for C_52_H_47_N_4_O_8_ 855.33884; found, 855.33582. To a solution of 5, 10, 15, 20-tetrakis(2′,6′-dimethoxyphenyl)-21H,23H-porphyrin (200 mg, 0.235 mmol) in dry CH_2_Cl_2_ (10 mL) was added with BBr_3_ (1.0 mL, 10.38 mmol) at 0 °C under N_2_. The resulting green solution was allowed to stir for 24 h at room temperature. Then 4.0 mL of water was added at 0 °C and the mixture was stirred for 40 min. A saturated NaHCO_3_ solution was added until the pH of the aqueous layer was around 7. Ethyl acetate (20 mL) was added to the suspension. The organic layer was separated, washed twice with water (20 mL), and then dried over anhydrous Na_2_SO_4_. The solvent was removed and the residue was purified by column chromatography (silica gel, 2:1 ethyl acetate/dichloromethane) to yield 5, 10, 15, 20-tetrakis(2′,6′-dihydroxyphenyl)-21H,23H-porphyrin as a purple powder (150 mg, 87%). ^1^H NMR (400 MHz, MeOD): *δ* 8.92 (s, 8H), 7.50 (t, *J* = 8.2 Hz, 4H), 6.84 (d, *J* = 8.2 Hz, 8H). HRMS (*m*/*z*): [M + H]^+^ calcd for C_44_H_31_N_4_O_8_ 743.21364; found, 743.21204. FeTDHPP was prepared by heating a dry methanol solution containing 5, 10, 15, 20-tetrakis(2′,6′-dihydroxyphenyl)-21H,23H-porphyrin (100 mg, 0.135 mmol), FeCl_2_·4H_2_O (270 mg, 1.35 mmol), and 2,6-lutidine (39 μL, 0.335 mmol) at 50 °C for 3 h under N_2_. After the solvent was removed, the resulting brown solid was dissolved in ethyl acetate (40 mL), added with 1.2 M HCl (40 mL), and stirred for 1 h. The organic layer was separated and washed several times with saturated NaCl solution until the pH was neutral. The organic solvent was removed and the crude product was purified by column chromatography (silica gel, ethyl acetate) to give FeTDHPP as a brown solid (100 mg, 89%). HRMS (*m*/*z*): [M-Cl]^+^ calcd for C_44_H_28_FeN_4_O_8_ 796.12511; found, 796.12343.

### Characterization

^1^H NMR and ^13^C NMR spectra were recorded at a Bruker advance III 400-MHz NMR instrument (Supplementary Figs. 52–65). UV–vis spectra were recorded on a Thermo Scientific GENESYS 50 UV–visible spectrophotometer. The FT–IR spectra were recorded using a Nicolet/Nexus-670 FT–IR spectrometer (ATR mode) (Supplementary Fig. 66). HRMS spectra were obtained on a Thermo Fisher Scientific Orbitrap Q Exactive ion trap mass and Thermo Fisher Scientific LTQ Orbitrap Elite (Supplementary Figs. 67–72). Dynamic light-scattering experiments were tested with a Brookhaven Elite Sizer zata-potential and a particle-size analyzer. GC/MS experiments were performed with an Agilent 7890A-5975C instrument.

### Fluorescence quenching rate constant determination

A PS in DMF was degassed by N_2_ or CO_2_ for 15 min in a sealed quartz cuvette with a septum cap. Different from other PSs, the absorption spectrum of PS **5** changes slightly under N_2_ when BIH was added (Supplementary Fig. 22). Therefore, the fluorescence quenching experiments for PS **5** were carried out under CO_2_ and those for the rest of the PSs were performed under N_2_. An identical excited-state lifetime for the PS was found either under N_2_ or CO_2_ (Supplementary Fig. 73). During the experiments, different concentrations of BIH were added to the solution of PS under N_2_ or CO_2_. The steady-state fluorescence for solution samples was measured by Duetta fluorescence and absorbance spectrometer. The excited-state lifetime of the photosensitizer was measured with an FLS 980 fluorescence spectrometer (Edinburgh instruments), in which a picosecond pulsed diode laser (*λ* = 472 and 406.2 nm) (Edinburgh instruments EPL-470) was used as the excitation source. The *λ*_max_ of emission for each photosensitizer is selected as the emission wavelength. The instrumental response function (IRF) of the instrument was measured using silicon oxide (30% in H_2_O) (Supplementary Fig. 74). The *k*_q_ was calculated by the Stern–Volmer equation:

1 $${I}_{0}/I\,{{{{{\rm{or}}}}}}\,{\tau }_{0}/\tau=1+{k}_{q}\times {\tau }_{0}\times [Q]$$

where *I*_*0*_ and *I* represent the fluorescence intensity of the photosensitizer in the absence and presence of a quencher; *τ*_*0*_ and *τ* is the lifetime of the photosensitizer in the absence and presence of the quencher; *k*_q_ is the quenching rate constant; [*Q*] is the concentration of the quencher BIH.

### Electrochemical measurements

Electrochemical studies were performed using a CHI-760E electrochemical analyzer using a single-compartment cell with a glassy carbon working electrode (3.0 mm in diameter), a platinum auxiliary electrode, and a SCE reference electrode. The electrolyte solution was 0.1 M tetrabutyl hexafluoroammonium phosphate in DMF. The solution was purged with N_2_ or CO_2_ for at least 30 min before measurement. All potentials reported in this study were referred to SCE.

### Photocatalytic CO_2_ reduction

Photocatalytic experiments were conducted in a closed scintillation vial with rubber plug and magnetic stirring. The headspace of the vial was 51.8 mL. A reaction mixture (5.0 mL) was bubbled with CO_2_ for 25 min and then irradiated with a LED light setup (*λ* > 400 nm, or *λ* = 450 nm, or *λ* = 525 nm, PCX-50 C, Beijing Perfectlight Technology Co., Ltd.) or a 300 W Xe lamp (PLS-SXE-300, Beijing Perfect light) equipped with a 550 nm cut-off filter. The gaseous products were analyzed by Shimadzu GC–2014 gas chromatography equipped with a Shimadzu Molecular Sieve 13 × 80/100 3.2 × 2.1 mm × 3.0 m and a Porapak N 3.2 × 2.1 mm × 2.0 m columns. A thermal conductivity detector (TCD) was used to detect H_2_ and a flame ionization detector (FID) with a methanizer was used to detect CO and other hydrocarbons. Nitrogen was used as the carrier gas. The oven temperature was kept at 60 °C. The TCD detector and injection port were kept at 100 °C and 200 °C, respectively. Specifically, systems containing 60 mM BIH (0.3 mmol, 67.2 mg), 0.6 μM FeTDHPP (0.003 μmol, 2.5 μg) and 20 μM PS (0.1 μmol, 22.3 μg **1**; 23.8 μg **2**; 23.8 μg **3**; 23.9 μg **4**; 31.8 μg **5**; and 38.2 μg **6**) were used for the calculation of TON_Fe_, TOF_Fe_ and Sel_CO_. Systems containing 60 mM BIH (0.3 mmol, 67.2 mg), 20 μM FeTDHPP (0.1 μmol, 83.2 μg) and 5 μM PS (0.025 μmol, 5.6 μg **1**; 6.0 μg **2**; 6.0 μg **3**; 6.0 μg **4**; 8.0 μg **5**; and 9.6 μg **6**) were used for the calculation of the yield of CO and TON_PS_.

### Quantum yield of CO production

The experiments were carried out under monochromic light of 450 nm obtained using a blue LED light setup (*λ* = 450 nm, PCX-50C, Beijing Perfectlight Technology Co., Ltd.). The blank was a DMF solution containing 60 mM BIH and 20 μM FeTDHPP. Systems containing 60 mM BIH (0.3 mmol, 67.2 mg), 20 μM FeTDHPP (0.1 μmol, 83.2 μg) and 20 μM PS (0.1 μmol, 22.3 μg **1**; 23.8 μg **2**; 23.8 μg **3**; 23.9 μg **4**; 31.8 μg **5**; and 38.2 μg **6**) were used for the calculation of the quantum yields. The difference between the power (*P*) of light passing through the blank and through the sample containing the photosensitizer was measured by a FZ-A Power meter (Beijing Normal University Optical Instrument Company). The quantum yield (*Φ*) was calculated after 1 h irradiation according to the following equation:

2 $$\varPhi=\frac{2\times n({{{{{\rm{CO}}}}}})\times {N}_{A}}{{PSt}\times \frac{\lambda }{hc}}$$

where *n* (CO) is the number of CO molecules produced, *N*_*A*_ is the Avogadro constant (6.02 × 10^23^ mol^−1^), *S* is the incident irradiation area (6.33 cm^2^), *t* is the irradiation time (in second), *λ* is the incident wavelength (450 nm), *h* is the Plank constant (6.626 × 10^−34^ J s), and *c* is the speed of light (3.0 × 10^8^ m s^−1^).

### Fluorescence quantum yield

Fluorescence quantum yields of PSs (listed in Table 1) were calculated according to a literature method^50^. A PS was dissolved in DMF and bubbled with N_2_ for at least 10 min in a quartz cuvette (10-mm path length). Rhodamine 6G (R6G) was used as a standard sample. UV–vis spectra (Supplementary Fig. 75) were measured with Thermo Scientific GENESYS 50 UV–visible spectrophotometer and emission spectra (Supplementary Fig. 76) were acquired on a Duetta fluorescence and absorbance spectrometer. The absorption of photosensitizers were adjusted to the same as that of R6G at *λ* = 480 nm. The fluorescence quantum efficiency was calculated as follow:

3 $${\varPhi }_{x}=\,{\varPhi }_{{{{{{{\rm{st}}}}}}}}\left(\frac{{{{{{{{\rm{Abs}}}}}}}}_{{{{{{{\rm{st}}}}}}}}}{{{{{{{{\rm{Abs}}}}}}}}_{x}}\right)\left(\frac{{\eta }_{x}^{2}}{{\eta }_{{{{{{{\rm{st}}}}}}}}^{2}}\right)\left(\frac{{{{{{{{\rm{Area}}}}}}}}_{{{{{{{\rm{Em}}}}}}}x}}{{{{{{{{\rm{Area}}}}}}}}_{{{{{{{\rm{Em}}}}}}}{{{{{{\rm{st}}}}}}}}}\right)$$

where ${\varPhi }_{{{{{{{\rm{st}}}}}}}}$ is the fluorescence quantum yield of R6G (${\varPhi }_{{{{{{{\rm{st}}}}}}}}$ = 0.95 in EtOH); *η* is the refractive index of solvent (*η*_EtOH_ = 1.3611, *η*_DMF_ = 1.4300); Area_Em_ is the emission integral area of the photosensitizer or R6G.

### Statistics and reproducibility

The statistical analysis is based on the original data without randomization and blind treatment. In order to ensure the reproducibility of the data, key experiments were conducted at least three separated runs with freshly prepared solutions on different days.

## Supplementary information

Supplementary Information

Peer Review File

## Data Availability

The data that support the findings of this study are available from the corresponding author on reasonable request. Source data are provided with this paper.

## Source data

Source Data
