# Supplementary material for: Photocatalytic CO2 reduction with aminoanthraquinone organic dyes
Source: Nat Commun. 2023 Feb 25;14:1087. doi: 10.1038/s41467-023-36784-7 (PMC9968311; doi:10.1038/s41467-023-36784-7)
Supplement: Supplementary file 1 — Supplementary Information [file 41467_2023_36784_MOESM1_ESM.pdf]

Supplementary Information

Photocatalytic CO_2 reduction with aminoanthraquinone organic dyes

Qinqin Lei[1,2], Huiqing Yuan[1,2], Jiehao Du[1], Mei Ming[1], Shuang Yang[1], Ya Chen[1], Jingxiang Lei[1], and Zhiji Han*[1]

[1] MOE Key Laboratory of Bioinorganic and Synthetic Chemistry, School of Chemistry, Sun Yat-sen University, Guangzhou, 510275, China.

[2] These authors contributed equally: Qinqin Lei, Huiqing Yuan.

* email: hanzhiji@mail.sysu.edu.cn.

Table of Contents

**Supplementary Figure 1-6**	UV-vis absorption spectra of **1-6**	**S3-5**
**Supplementary Figure 7**	UV-vis absorption and emission spectra of **1-6**	**S6**
**Supplementary Figure 8-9**	Cyclic voltammograms of **1-6**	**S7-8**
**Supplementary Figure 10**	Square wave voltammetry of **1-6**	**S9**
**Supplementary Figure 11**	Photocatalytic CO_2 reduction by varying [BIH]	**S10**
**Supplementary Figure 12**	Photocatalytic CO_2 reduction by varying [FeTDHPP]	**S10**
**Supplementary Figure 13**	Photocatalytic CO_2 reduction by varying [**5**]	**S11**
**Supplementary Figure 14**	Stability tests of photocatalytic systems	**S12**
**Supplementary Figure 15**	Dynamic light scattering (DLS) measurements	**S13**
**Supplementary Figure 16**	Mercury poisoning experiments	**S13**
**Supplementary Figure 17**	GC/MS chromatograms of CO	**S14**
**Supplementary Figure 18-19**	Fluorescence emission quenching of **4-5**	**S15**
**Supplementary Figure 20**	Fluorescence lifetime quenching of **1-6**	**S16**
**Supplementary Figure 21**	UV-vis absorption spectra of **1-6** with addition of FeTDHPP	**S17**
**Supplementary Figure 22**	UV-vis absorption spectra of **1-6** with addition of BIH	**S18**
**Supplementary Figure 23-28**	^{1}H NMR spectra **of 1-6** with addition of BIH	**S19-21**
**Supplementary Figure 29**	UV-vis absorption spectra of **5** with addition of FeTDHPP	**S22**
**Supplementary Figure 30-36**	The change of UV-vis spectra of **1-6** during photolysis	**S22-24**
**Supplementary Figure 37**	UV-vis spectrum of AQ upon reduction by $NaBH_4$	**S25**
**Supplementary Figure 38**	^{1}H NMR studying the reduction of AQ by $NaBH_4$	**S25**
**Supplementary Figure 39-40**	Absorption, emission, and emission lifetime of AQH_2	**S26**
**Supplementary Figure 41**	Luminescence lifetime quenching of AQH_2	**S27**
**Supplementary Figure 42-43**	UV-vis spectra of photolyzed **5** with addition of FeTDHPP	**S27-28**
**Supplementary Figure 44**	SWVs of systems before and after irradiation	**S28**
**Supplementary Figure 45**	UV-vis studying the change of FeTDHPP during photolysis	**S29**
**Supplementary Figure 46**	Photocatalytic CO_2 reduction with low [BIH]	**S29**
**Supplementary Figure 47-50**	UV-vis spectral change at >550 nm and 400 nm	**S30-31**
**Supplementary Figure 51**	UV-vis studying a mixture of FeTDHPP and BIH during photolysis	**S32**
**Supplementary Figure 52-72**	^{1}H NMR, ^{13}C NMR, FT-IR and HRMS spectra of PSs	**S33-44**
**Supplementary Figure 73**	Emission delay of **6** under N_2 and CO_2	**S45**
**Supplementary Figure 74**	Emission delay of the picosecond pulsed diode laser	**S45**
**Supplementary Figure 75-76**	Fluorescence quantum yield experiments	**S46-47**
**Supplementary Table 1**	Thermodynamic driving force of PSs	**S48**
**Supplementary Table 2-3**	Data for photocatalytic CO_2 reduction	**S48**
**Supplementary Table 4**	Summary of systems with organic photosensitizers	**S49-50**
**Supplementary Table 5**	Data for photocatalytic CO_2 reduction with **5**	**S51**
**Reference**		**S52**

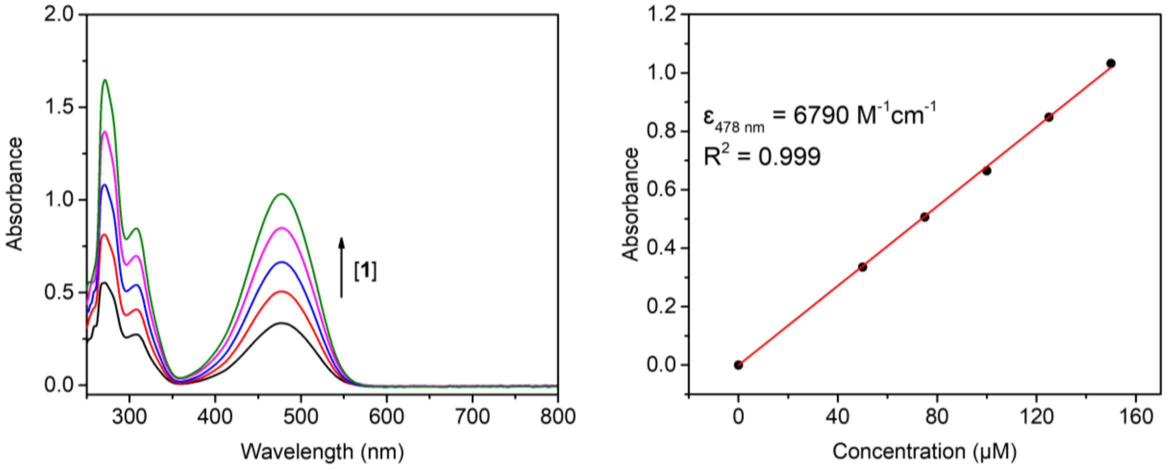

Supplementary Figure 1. UV-vis spectra. UV-vis spectra of PS **1** at different concentrations (50 to 150 μM) in DMF in a quartz cuvette (10–mm path length) at 298 K (left); a linear plot of absorbance at 478 nm (right). Source data are provided as a Source Data file.

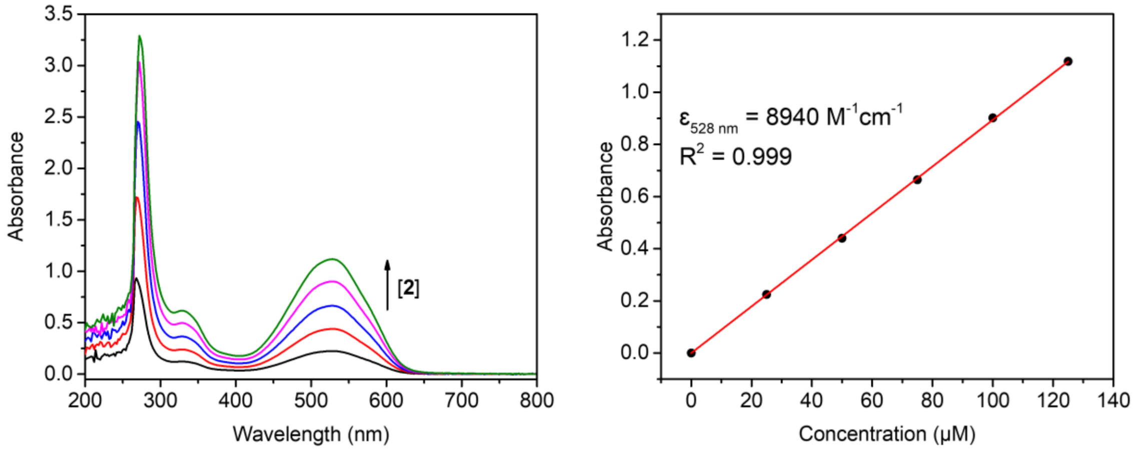

Supplementary Figure 2. UV-vis spectra. UV-vis spectra of PS **2** at different concentrations (25 to 125 μM) in DMF in a quartz cuvette (10–mm path length) at 298 K (left); a linear plot of absorbance at 528 nm (right). Source data are provided as a Source Data file.

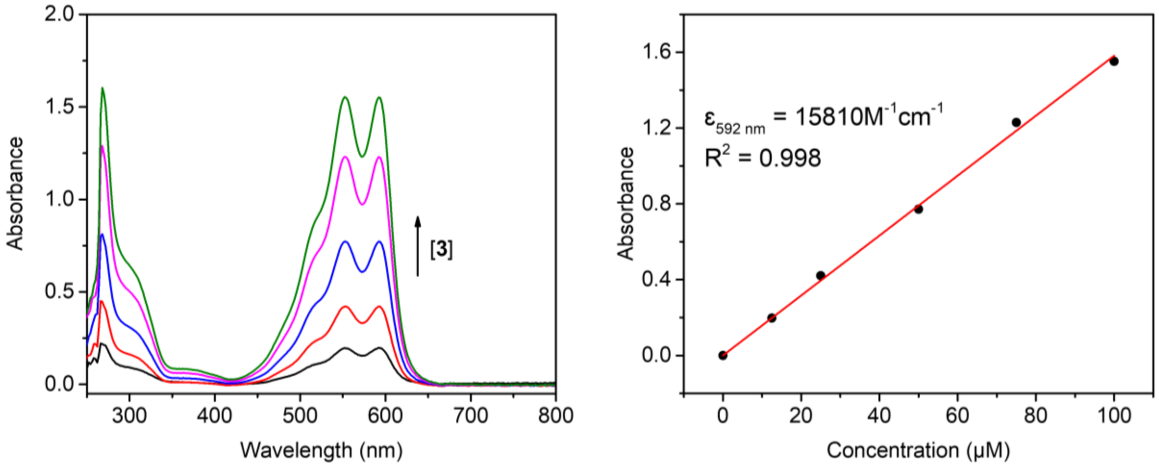

Supplementary Figure 3. UV-vis spectra. UV-vis spectra of PS **3** at different concentrations (12.5 to 100 μM) in DMF in a quartz cuvette (10–mm path length) at 298 K (left); a linear plot of absorbance at 592 nm (right). Source data are provided as a Source Data file.

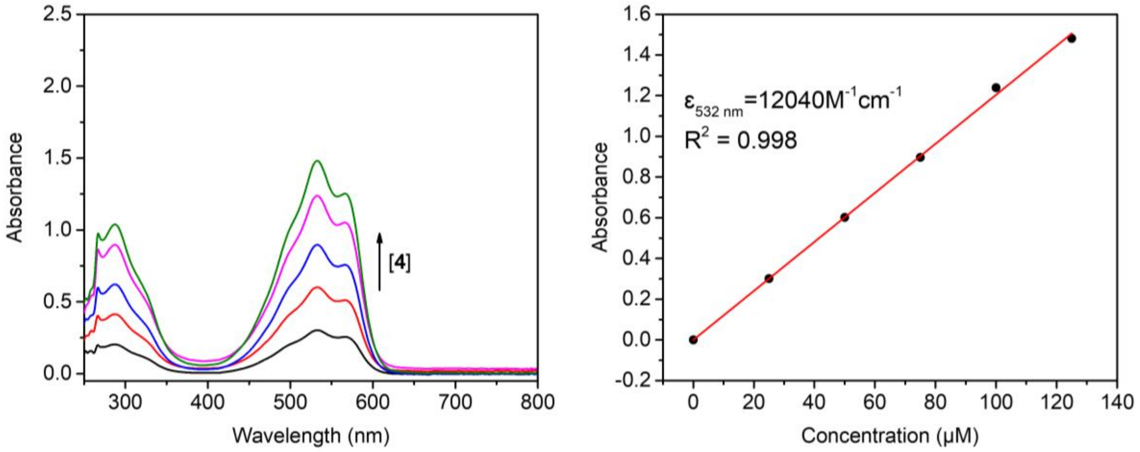

Supplementary Figure 4. UV-vis spectra. UV-vis spectra of PS **4** at different concentrations (25 to 125 μM) in DMF in a quartz cuvette (10–mm path length) at 298 K (left); a linear plot of absorbance at 532 nm (right). Source data are provided as a Source Data file.

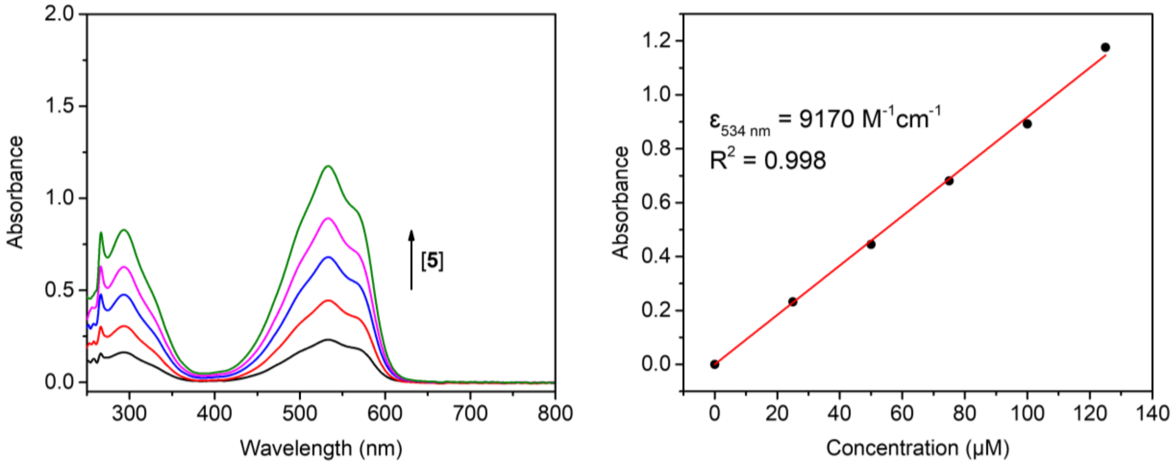

Supplementary Figure 5. UV-vis spectra. UV-vis spectra of PS **5** at different concentrations (25 to 125 μM) in DMF in a quartz cuvette (10–mm path length) at 298 K (left); a linear plot of absorbance at 534 nm (right). Source data are provided as a Source Data file.

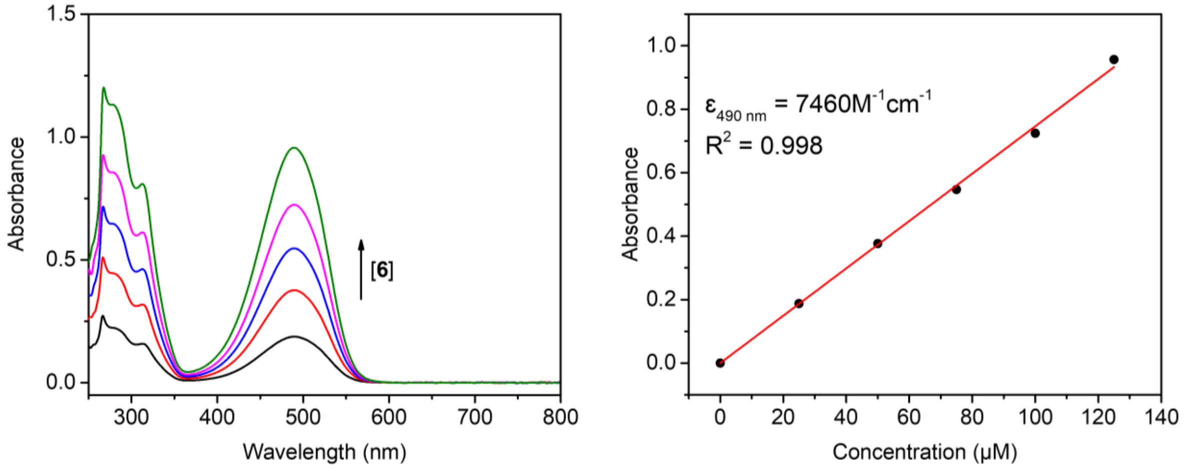

Supplementary Figure 6. UV-vis spectra. UV-vis spectra of PS **6** at different concentrations (25 to 125 μM) in DMF in a quartz cuvette (10–mm path length) at 298 K (left); a linear plot of absorbance at 490 nm (right). Source data are provided as a Source Data file.

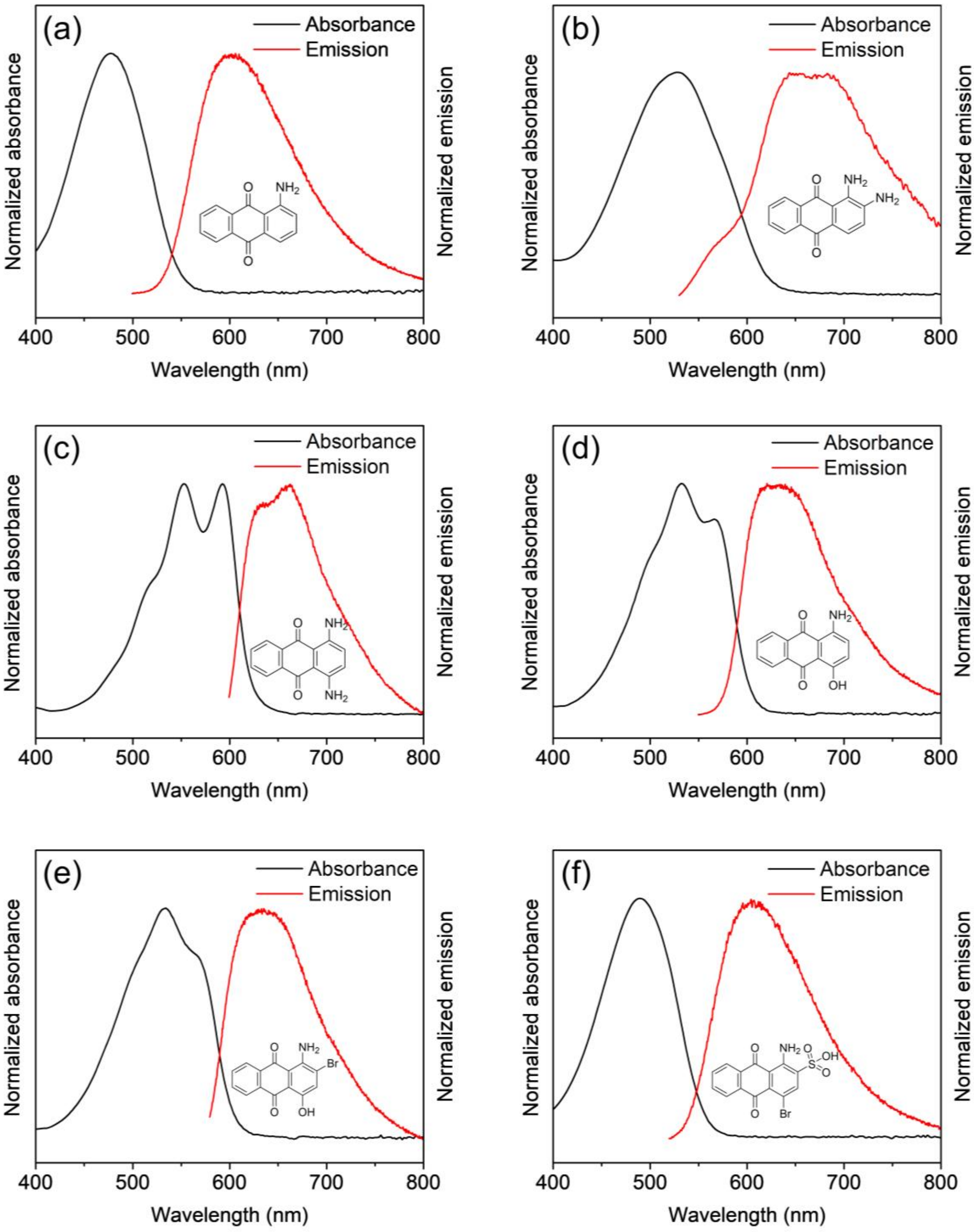

Supplementary Figure 7. UV-vis and emission spectra. Normalized absorption and emission spectra of (a) **1** (λ_{exc} = 470 nm); (b) **2** (λ_{exc} = 502 nm); (c) **3** (λ_{exc} = 540 nm); (d) **4** (λ_{exc} = 530 nm); (e) **5** (λ_{exc} = 530 nm); and (f) **6** (λ_{exc} = 490 nm) in DMF in a quartz cuvette (10–mm path length) at 298 K. Source data are provided as a Source Data file.

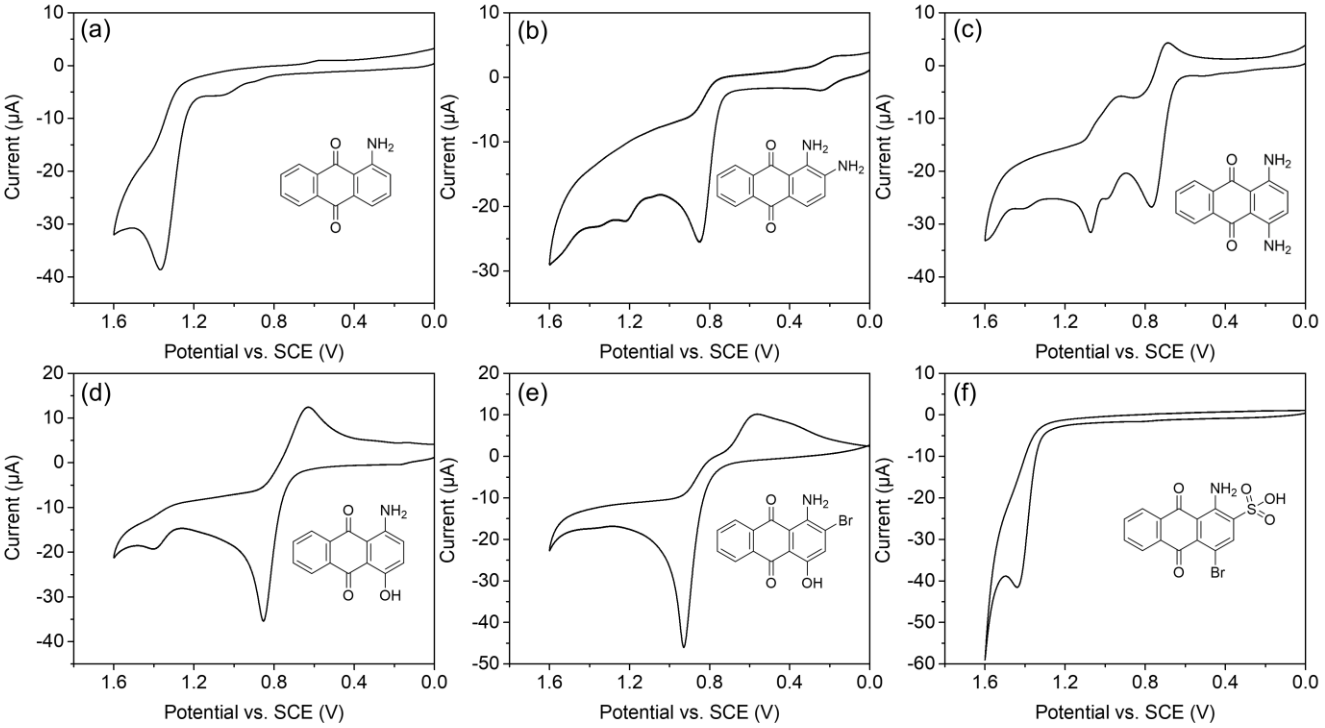

Supplementary Figure 8. Electrochemical study. Oxidative scans of cyclic voltammetry for 1.0 mM (a) **1**; (b) **2**; (c) **3**; (d) **4**; (e) **5**; and (f) **6** in 5 mL DMF containing 0.1 M $TBAPF_6$ under N_2. Experiments were conducted using a glassy carbon working electrode (3.0 mm in diameter), a Pt wire counter electrode, and a KCl-saturated calomel electrode at a scan rate of 100 $mV{\cdot}s^{-1}$. Source data are provided as a Source Data file.

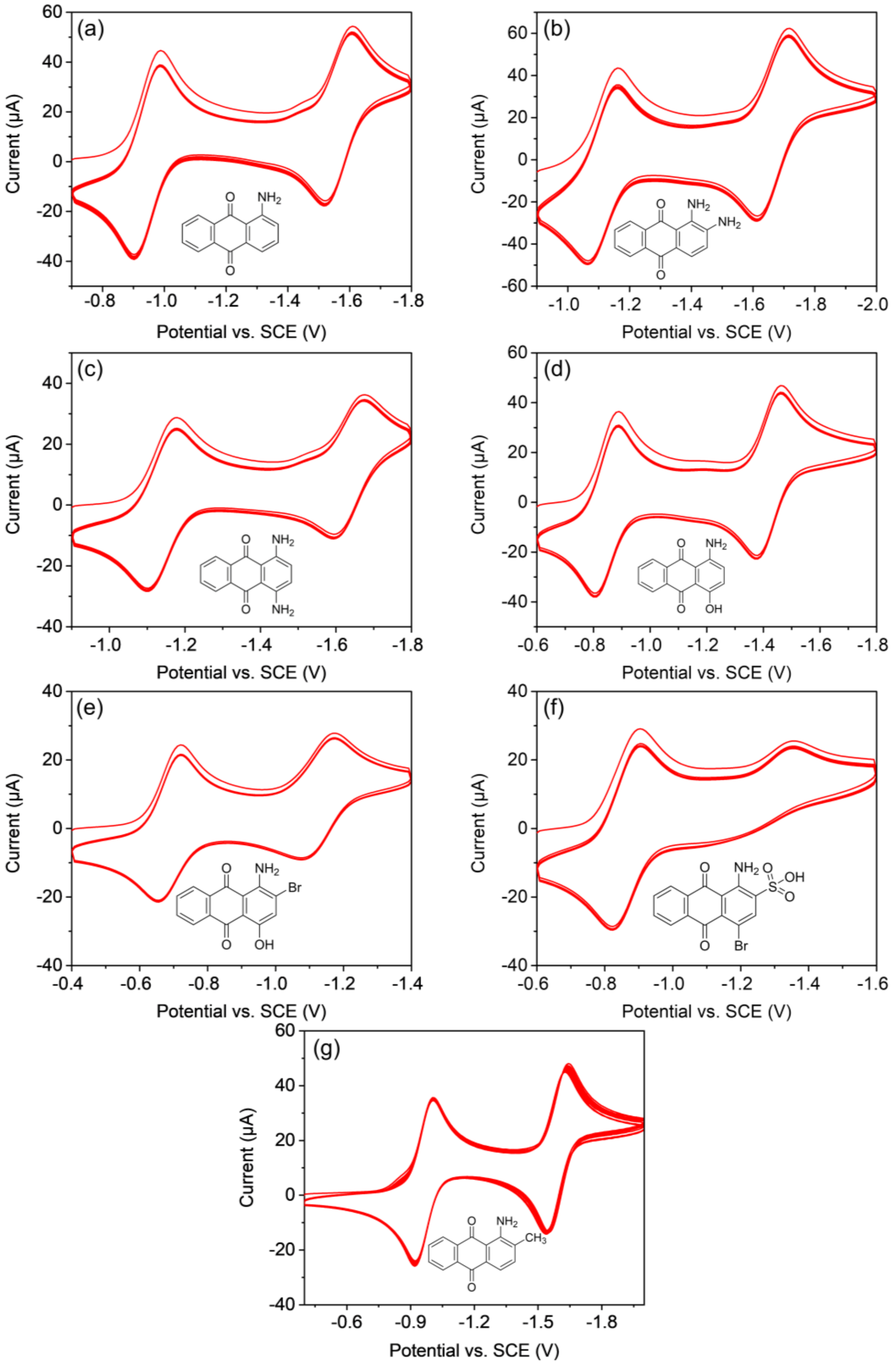

Supplementary Figure 9. Electrochemical study. Cyclic voltammograms of 1.0 mM (a) **1**; (b) **2**; (c) **3**; (d) **4**; (e) **5**; (f) **6**; and (g) 1-amino-2-methylanthraquinone in 5 mL DMF containing 0.1 M $TBAPF_6$ under N_2 over 10 cycles. Experiments were conducted using a glassy carbon working electrode (3.0 mm in diameter), a Pt wire counter electrode, and a KCl-saturated calomel electrode at a scan rate of 500 $mV{\cdot}s^{-1}$. Source data are provided as a Source Data file.

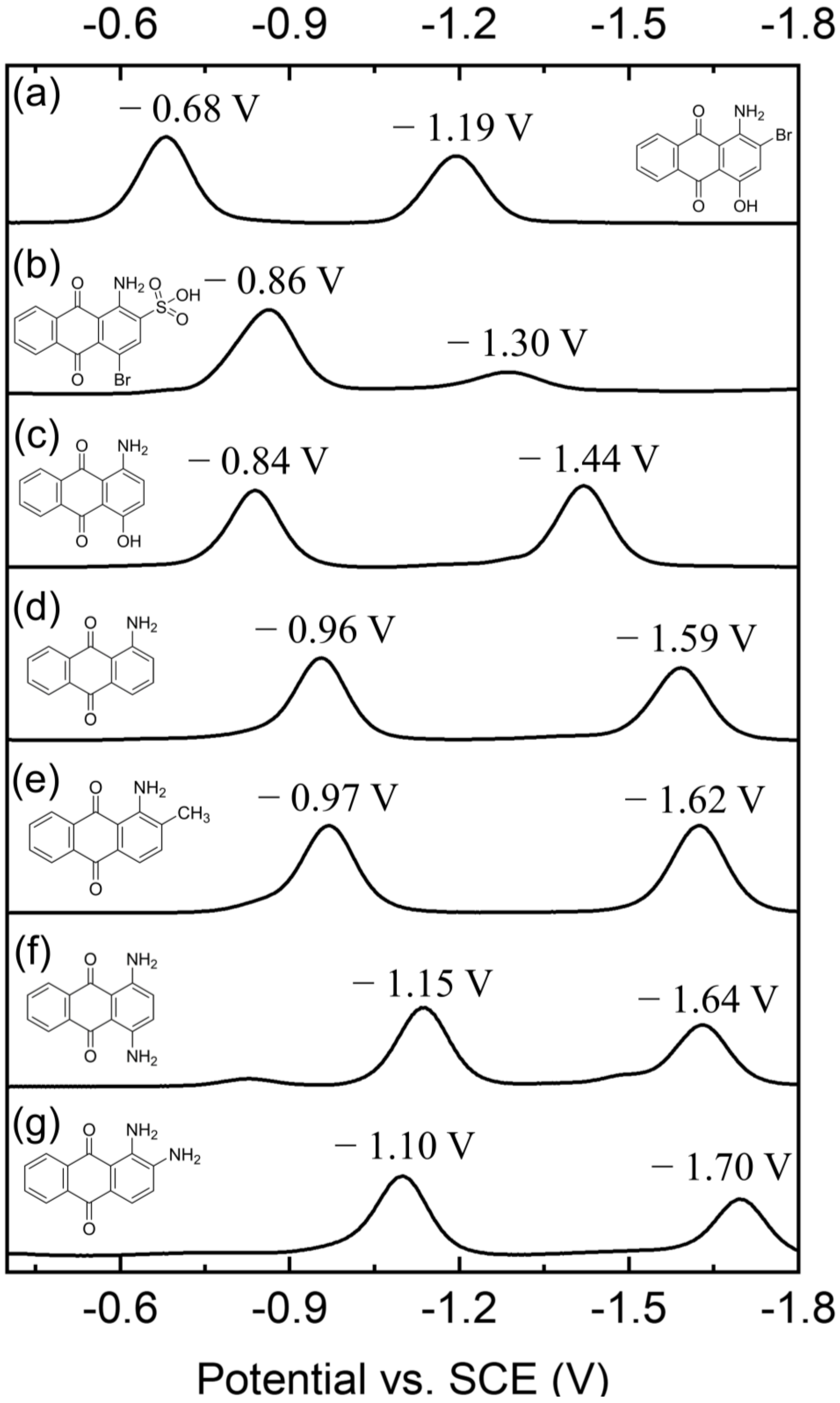

Supplementary Figure 10. Electrochemical study. Square wave voltammetry of 1.0 mM (a) **5**; (b) **6**; (c) **4**; (d) **1**; (e) 1-amino-2-methylanthraquinone; (f) **3**; and (g) **2** in 5 mL DMF containing 0.1 M $TBAPF_6$ under N_2. Experiments were conducted using a glassy carbon working electrode (3.0 mm in diameter), a Pt wire counter electrode, and a KCl-saturated calomel electrode at a scan rate of 100 $mV\cdot s^{-1}$. Source data are provided as a Source Data file.

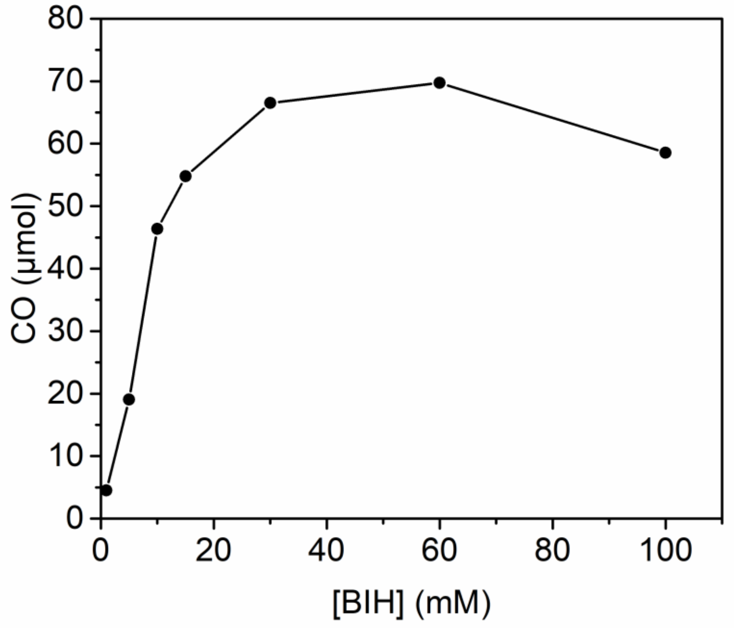

Supplementary Figure 11. Photocatalytic experiments. Amounts of CO generated from photocatalytic CO_2 reduction experiments in 5.0 mL CO_2-saturated DMF solution containing 20 μM **5** and 1.0 μM FeTDHPP with varying concentrations of BIH under white LED (λ > 400 nm, 100 mW/cm^2) at 298 K. Source data are provided as a Source Data file.

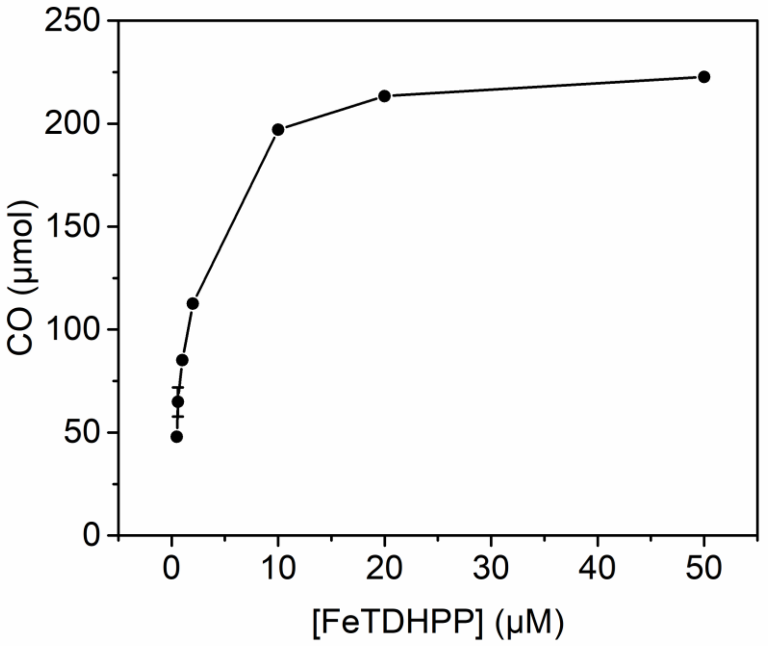

Supplementary Figure 12. Photocatalytic experiments. Amounts of CO generated from photocatalytic CO_2 reduction experiments in 5.0 mL CO_2-saturated DMF solution containing 20 μM **5** and 60 mM BIH with varying concentrations of FeTDHPP under white LED (λ > 400 nm, 100 mW/cm^2) at 298 K. The error bar denotes standard deviation, based on 3 separated runs. Source data are provided as a Source Data file.

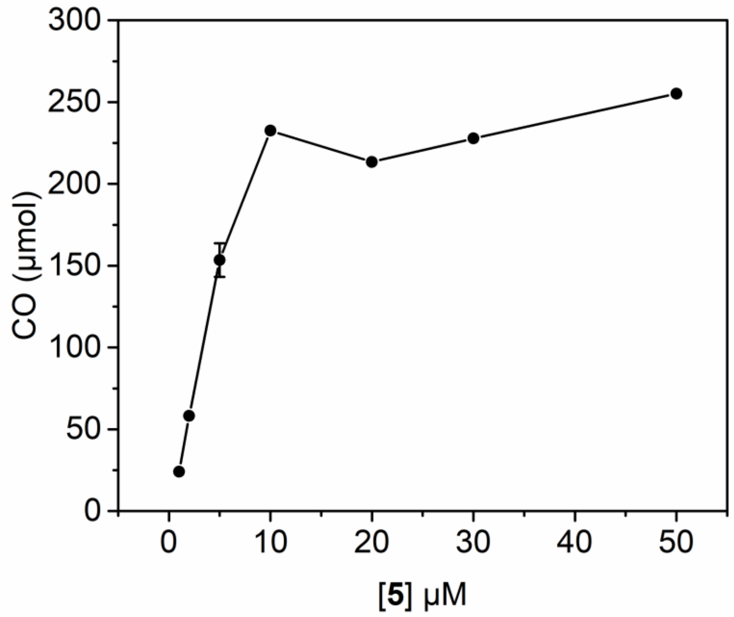

Supplementary Figure 13. Photocatalytic experiments. Amounts of CO generated from photocatalytic CO_2 reduction experiments in 5.0 mL CO_2-saturated DMF solution containing 20 μM FeTDHPP and 60 mM BIH with varying concentrations of **5** under white LED (λ > 400 nm, 100 mW/cm^2) at 298 K. The error bar denotes standard deviation, based on 3 separated runs. Source data are provided as a Source Data file.

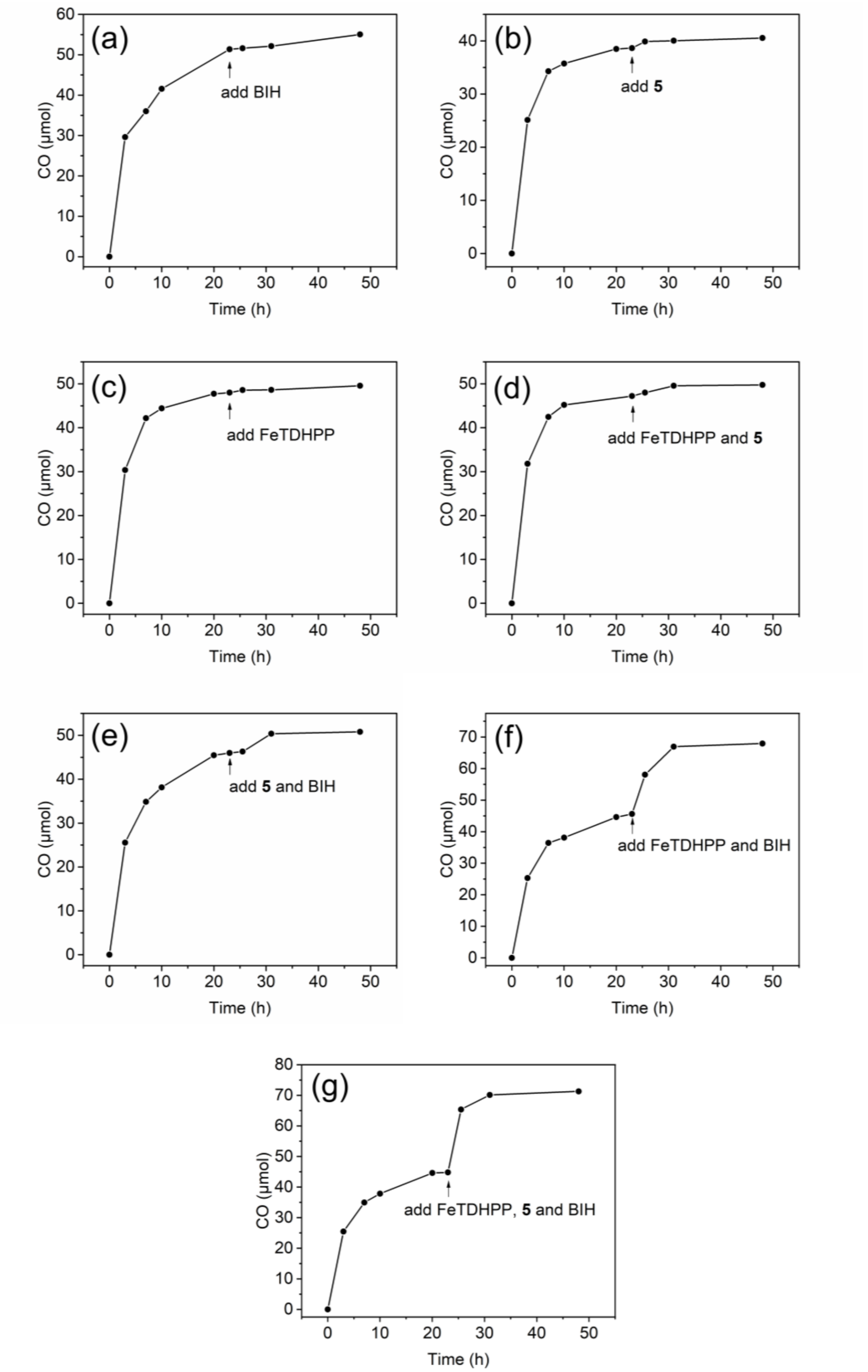

Supplementary Figure 14. Stability tests of photocatalytic systems. Initial photocatalytic systems containing 10 mM BIH, 1.0 μM FeTDHPP, and 0.02 mM **5** in CO_2-saturated DMF (5.0 mL) under white LED (λ > 400 nm, 100 mW/cm^2) at 298 K. Another equiv. of BIH (a), **5** (b), FeTDHPP (c), FeTDHPP/**5** (d), **5**/BIH (e), FeTDHPP/BIH (f) or FeTDHPP/**5**/BIH (g) in DMF (50 μL) was added to the reaction vails with a microsyringe at 23 h. The amount of the component added is the same as that in the initial system. Source data are provided as a Source Data file.

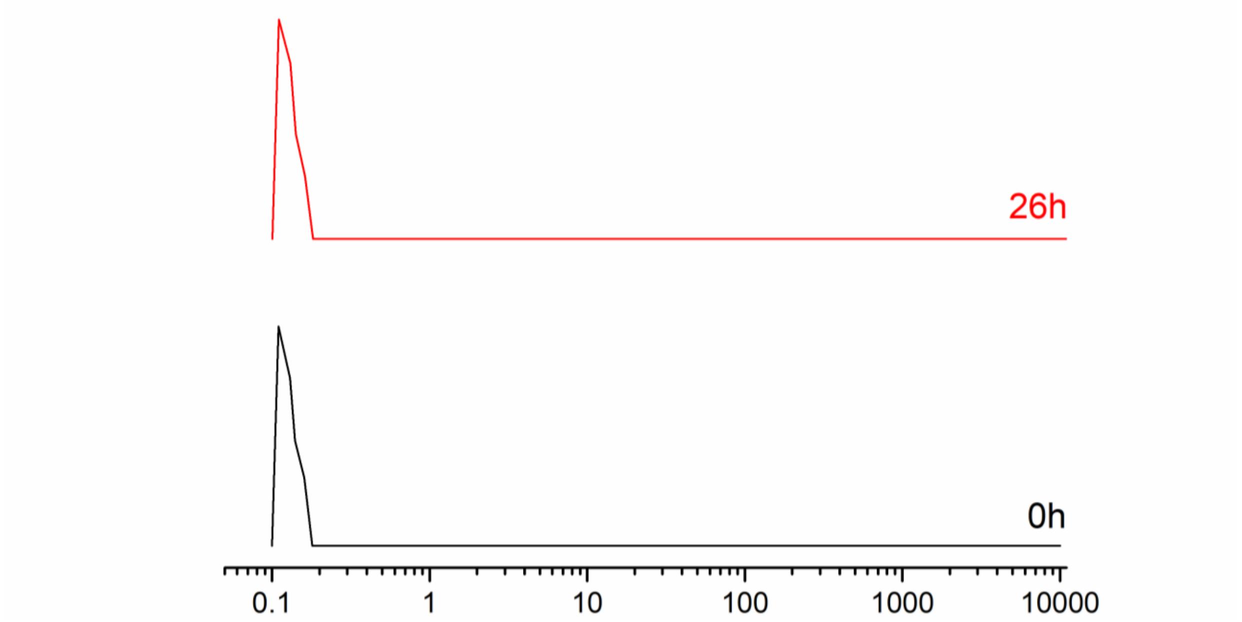

Supplementary Figure 15. Dynamic light scattering (DLS) measurements. Particle size distribution of a CO_2-saturated DMF solution containing 10 mM BIH, 1.0 μM FeTDHPP, and 0.02 mM **5** determined by DLS before and after irradiation under white LED (λ > 400 nm, 100 mW/cm^2) at 298 K for 26 h. Source data are provided as a Source Data file.

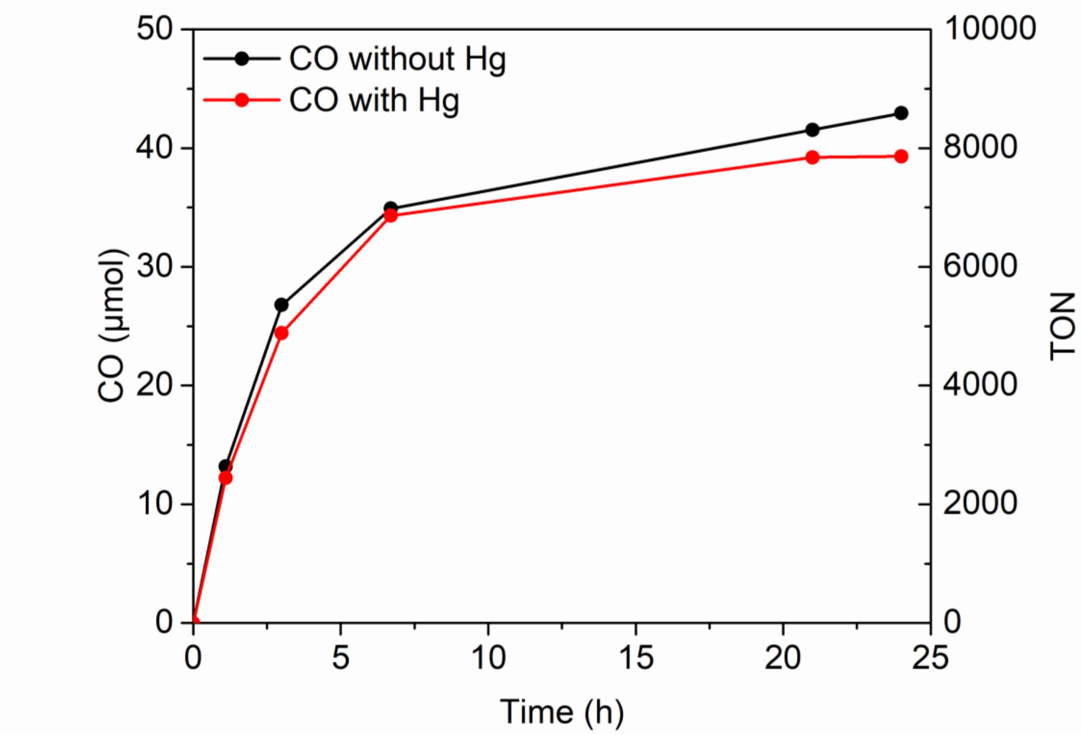

Supplementary Figure 16. Mercury poisoning experiments. CO generation in CO_2-saturated DMF solutions containing 10 mM BIH, 1.0 μM FeTDHPP, and 0.02 mM **5** in the presence and absence of Hg^0 (~0.02 mL) under white LED (λ > 400 nm, 100 mW/cm^2) at 298 K. Source data are provided as a Source Data file.

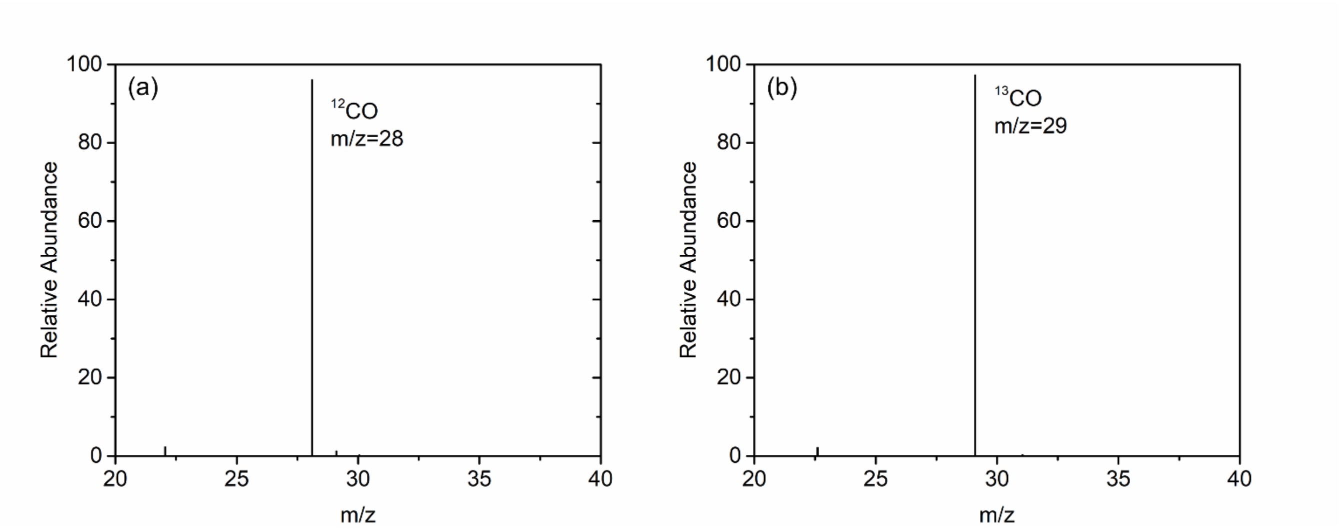

Supplementary Figure 17. GC/MS chromatograms of CO. Photocatalytic CO_2 reduction in (a) $^{12}CO_2$-saturated and (b) $^{13}CO_2$-saturated DMF solutions containing 2.0 μM FeTDHPP, 60 mM BIH, and 0.02 mM **5** under white LED (λ > 400 nm, 100 mW/cm^2) at 298 K. Source data are provided as a Source Data file.

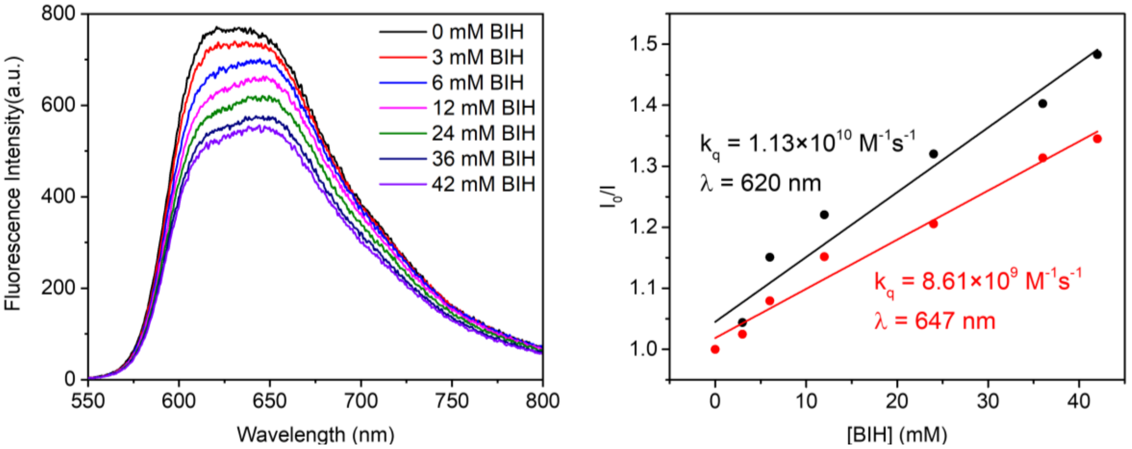

Supplementary Figure 18. Fluorescence emission quenching. Stern-Volmer plots at 620 nm and 647 nm (right) from fluorescence quenching (left) (λ_{exc} = 530 nm) of **4** (50 μM) by BIH in DMF in a quartz cuvette (10–mm path length) at 298 K under N_2. Source data are provided as a Source Data file.

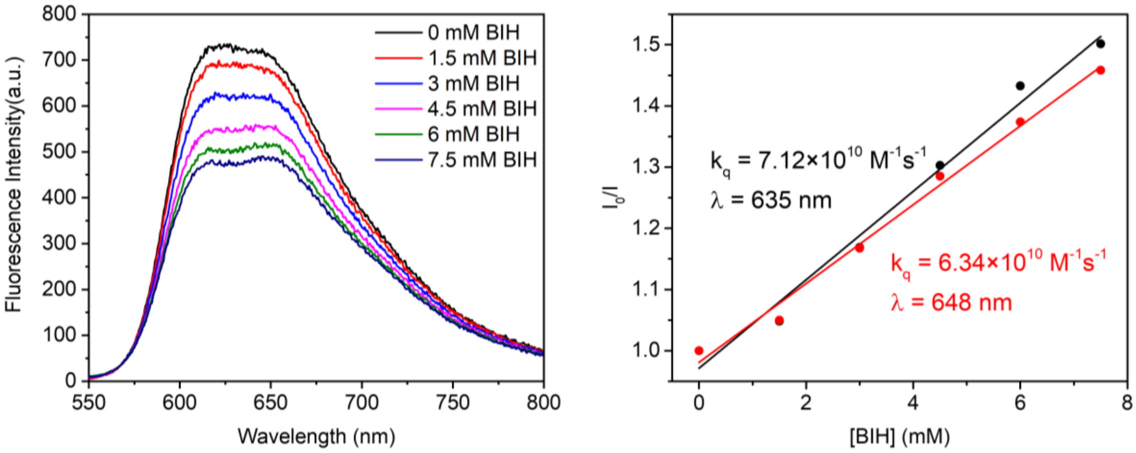

Supplementary Figure 19. Fluorescence emission quenching. Stern-Volmer plot at 635 nm and 648 nm (right) from fluorescence quenching (left) (λ_{exc}= 530 nm) of **5** (50 μM) by BIH in DMF in a quartz cuvette (10–mm path length) at 298 K under CO_2. Source data are provided as a Source Data file.

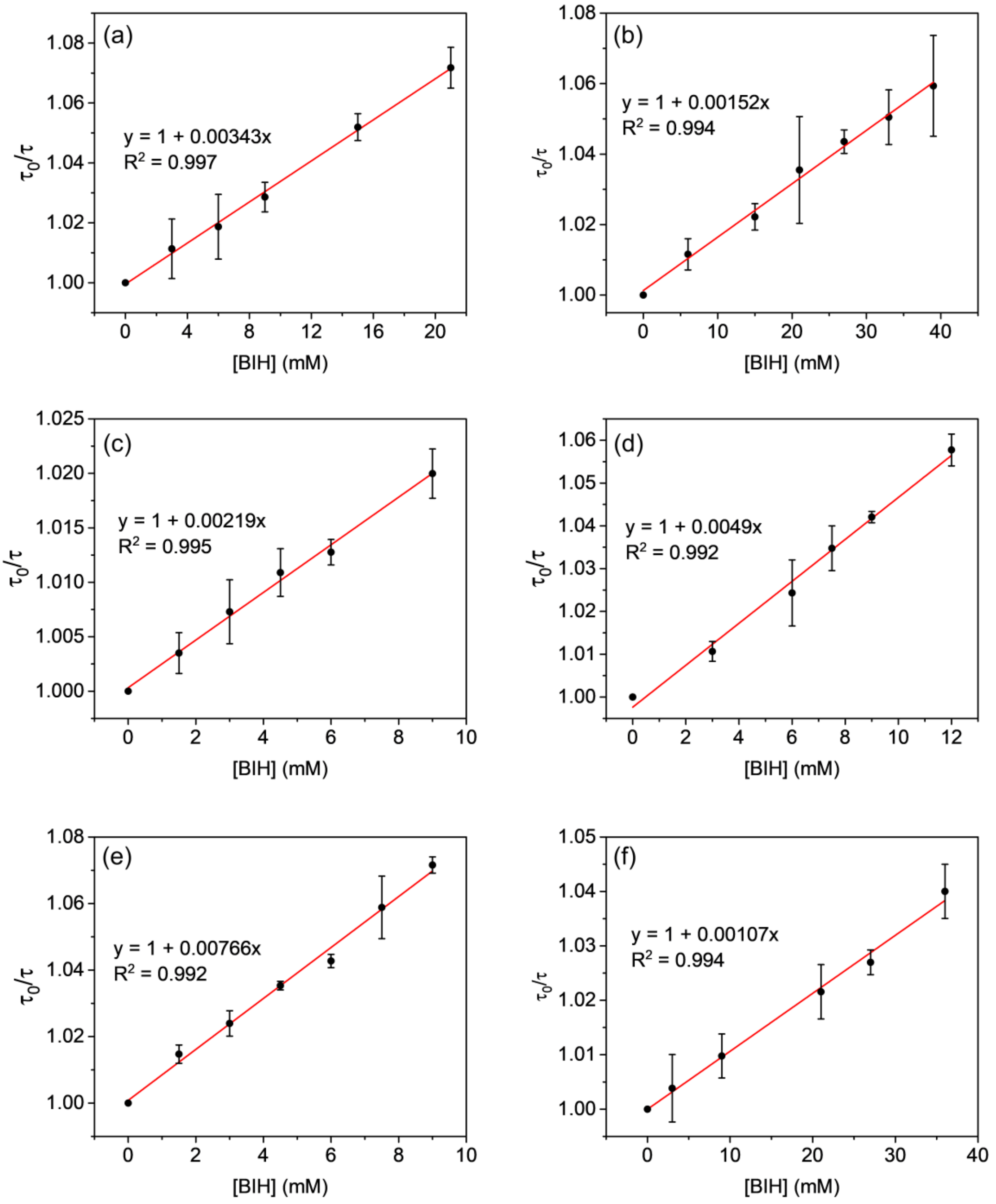

Supplementary Figure 20. Fluorescence lifetime quenching. Stern-Volmer plots of fluorescence lifetime quenching of 50 μM (a) **1** (λ_{em} = 600 nm); (b) **2** (λ_{em} = 650 nm); (c) **3** (λ_{em} = 662 nm); (d) **4** (λ_{em} = 620 nm); (e) **5** (λ_{em} = 635 nm); and (f) **6** (λ_{em} = 607 nm) in DMF in a quartz cuvette (10–mm path length) under N_2 (for **1**–**4** and **6**) or under CO_2 (for **5**) at 298 K. The error bars denote standard deviations, based on 3 separated runs. The excitation wavelength is 472 nm. Source data are provided as a Source Data file.

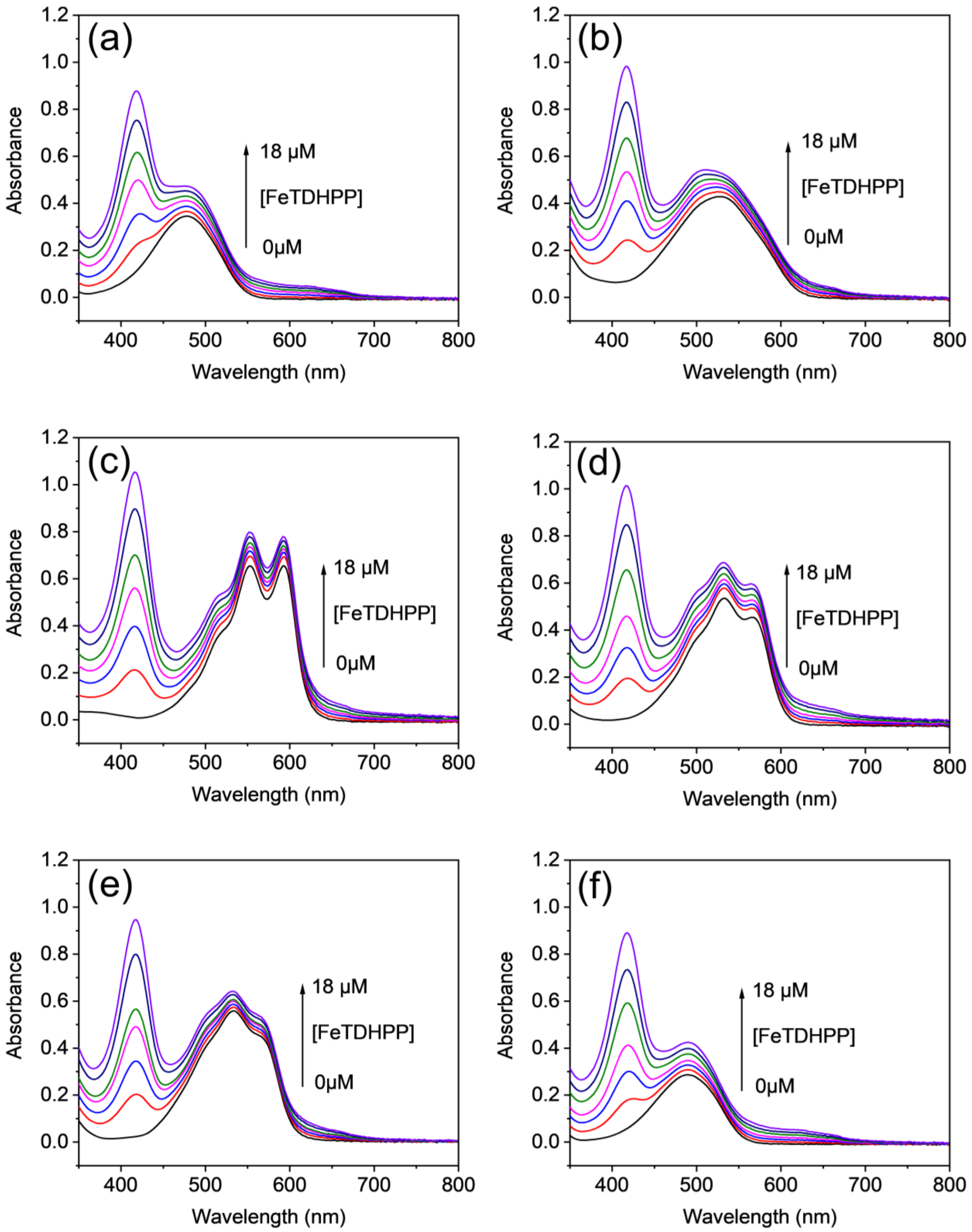

Supplementary Figure 21. UV–vis absorption spectra. UV–vis absorption spectra of 50 μM (a) **1**; (b) **2**; (c) **3**; (d) **4**; (e) **5**; and (f) **6** in DMF in a quartz cuvette (10–mm path length) at 298 K with addition of varying amounts of FeTDHPP. Source data are provided as a Source Data file.

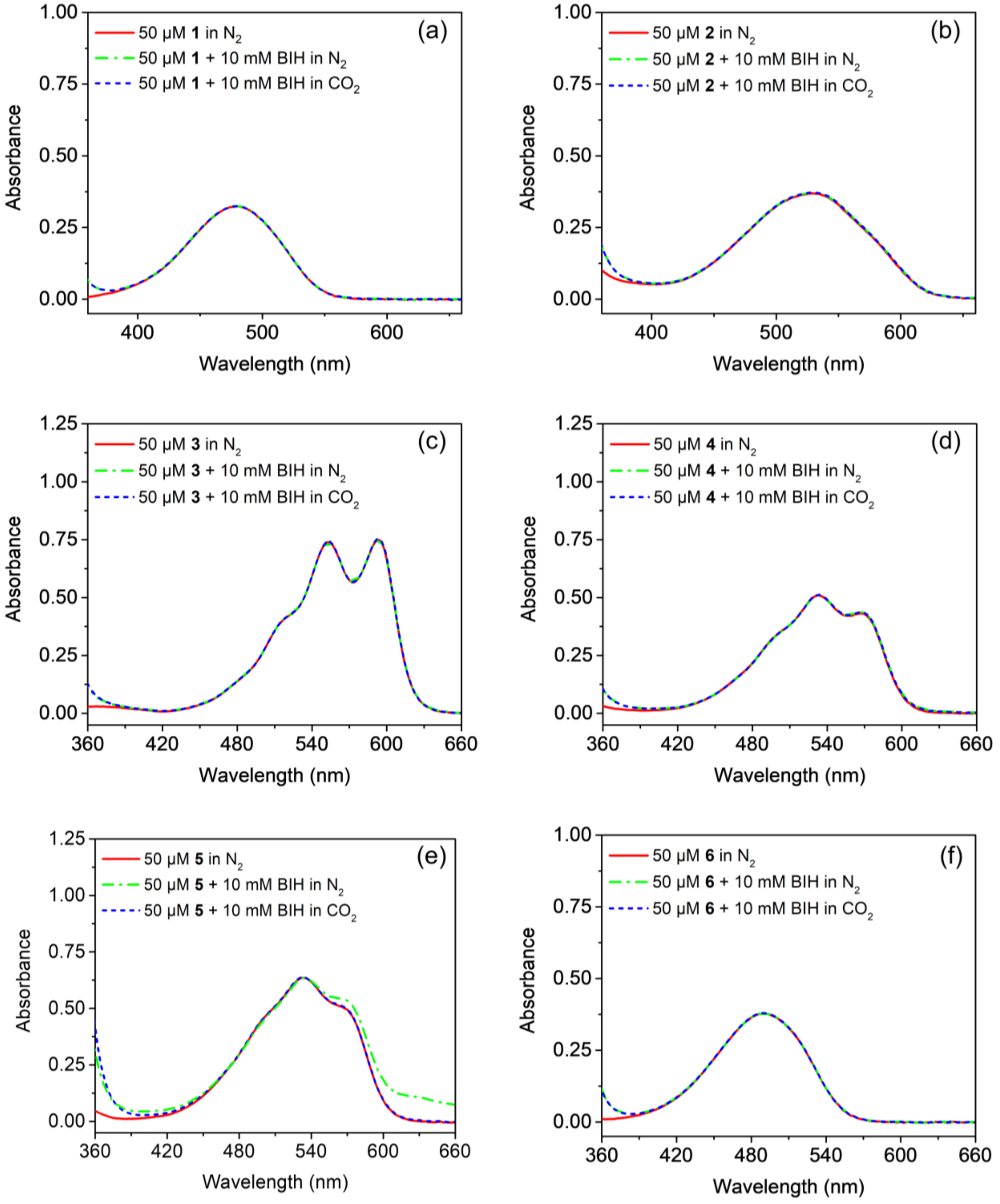

Supplementary Figure 22. UV-vis absorption spectra. UV-vis absorption spectra of 50 µM (a) **1**; (b) **2**; (c) **3**; (d) **4**; (e) **5**; and (f) **6**, with or without the presence of 10 mM BIH in DMF in a quartz cuvette (10–mm path length) under N_2 and CO_2 at 298 K. Source data are provided as a Source Data file.

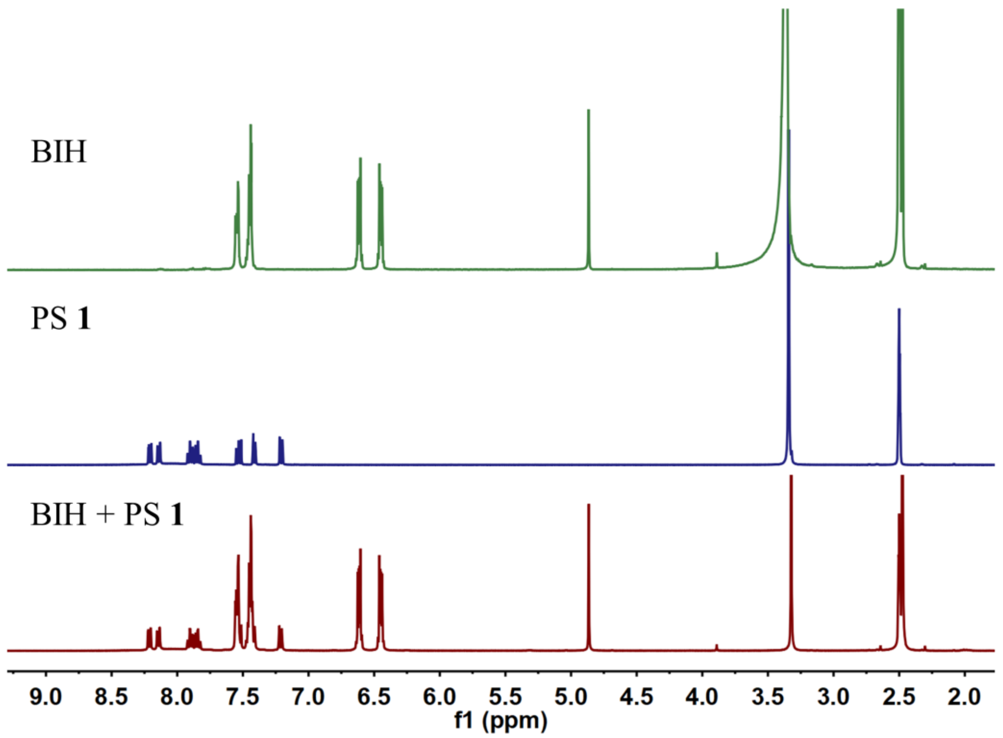

Supplementary Figure 23. ^{1}H NMR spectra. ^{1}H NMR (400 MHz, 298 K) spectra of BIH, PS **1**, and a mixture of BIH and PS **1** in d_6-DMSO in air.

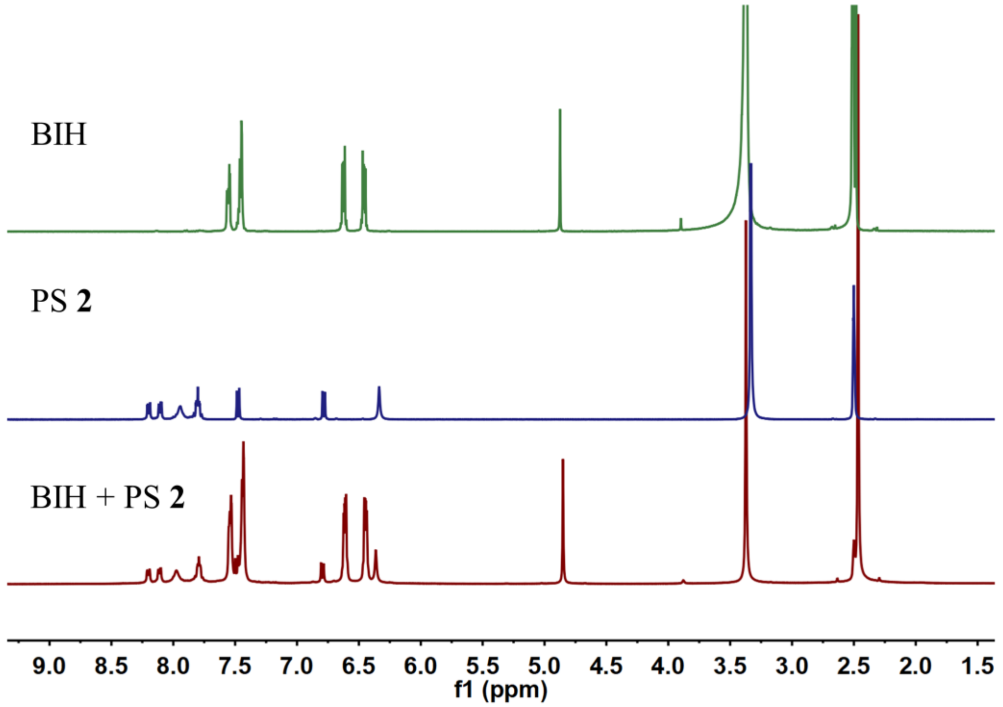

Supplementary Figure 24. ^{1}H NMR spectra. ^{1}H NMR (400 MHz, 298 K) spectra of BIH, PS **2**, and a mixture of BIH and PS **2** in d_6-DMSO in air.

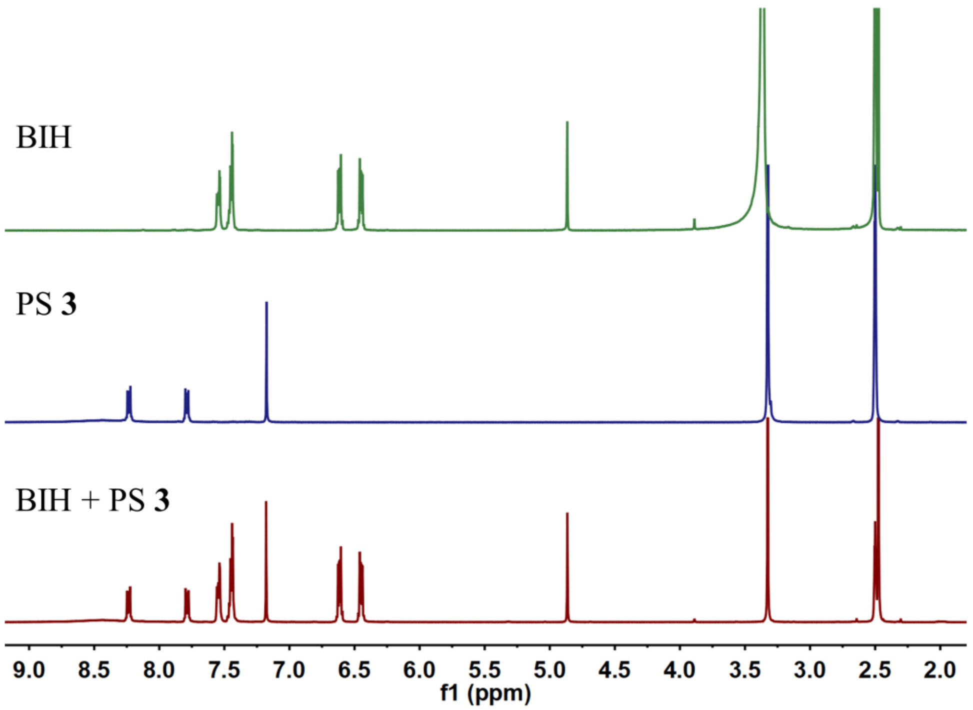

Supplementary Figure 25. ¹H NMR spectra. ¹H NMR (400 MHz, 298 K) spectra of BIH, PS **3**, and a mixture of BIH and PS **3** in d_6-DMSO in air.

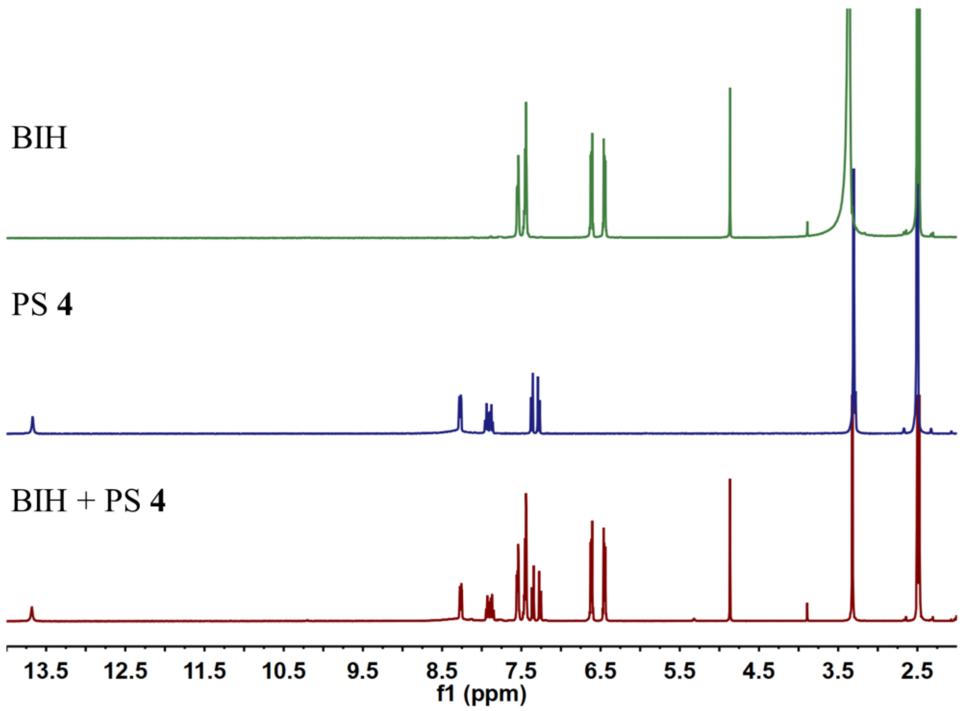

Supplementary Figure 26. ¹H NMR spectra. ¹H NMR (400 MHz, 298 K) spectra of BIH, PS **4**, and a mixture of BIH and PS **4** in d_6-DMSO in air.

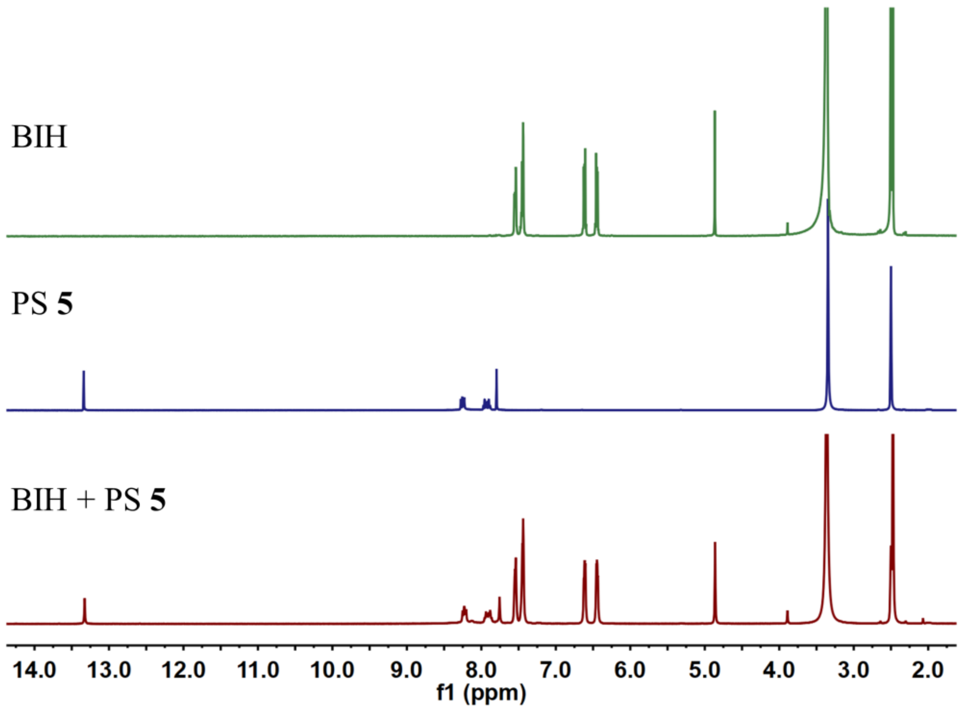

Supplementary Figure 27. ^{1}H NMR spectra. ^{1}H NMR (400 MHz, 298 K) spectra of BIH, PS **5**, and a mixture of BIH and PS **5** in d_6-DMSO under CO_2.

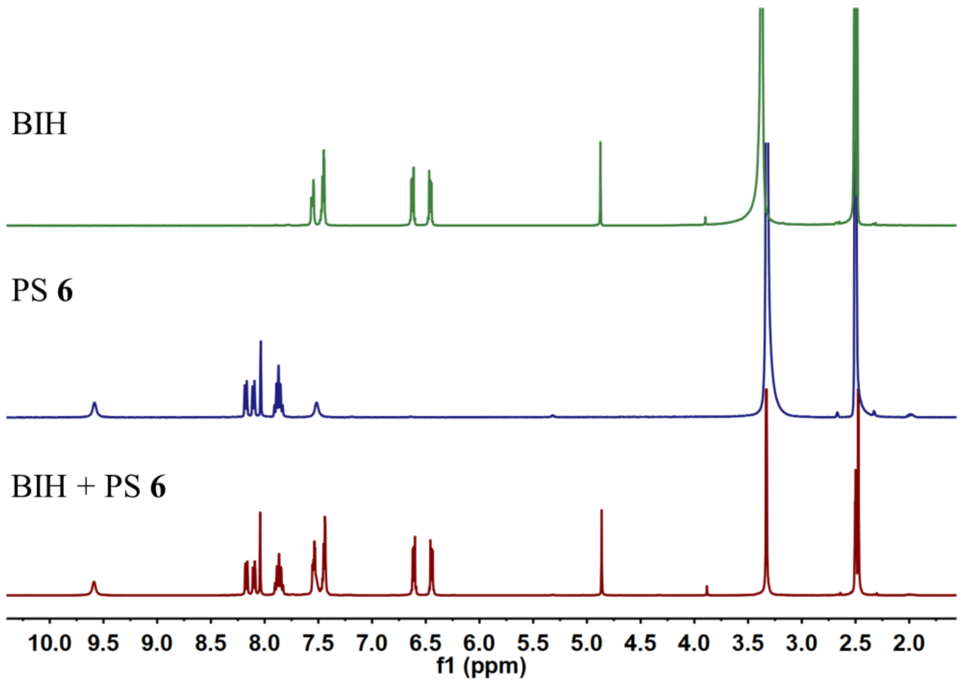

Supplementary Figure 28. ^{1}H NMR spectra. ^{1}H NMR (400 MHz, 298 K) spectra of BIH, PS **6**, and a mixture of BIH and PS **6** in d_6-DMSO in air.

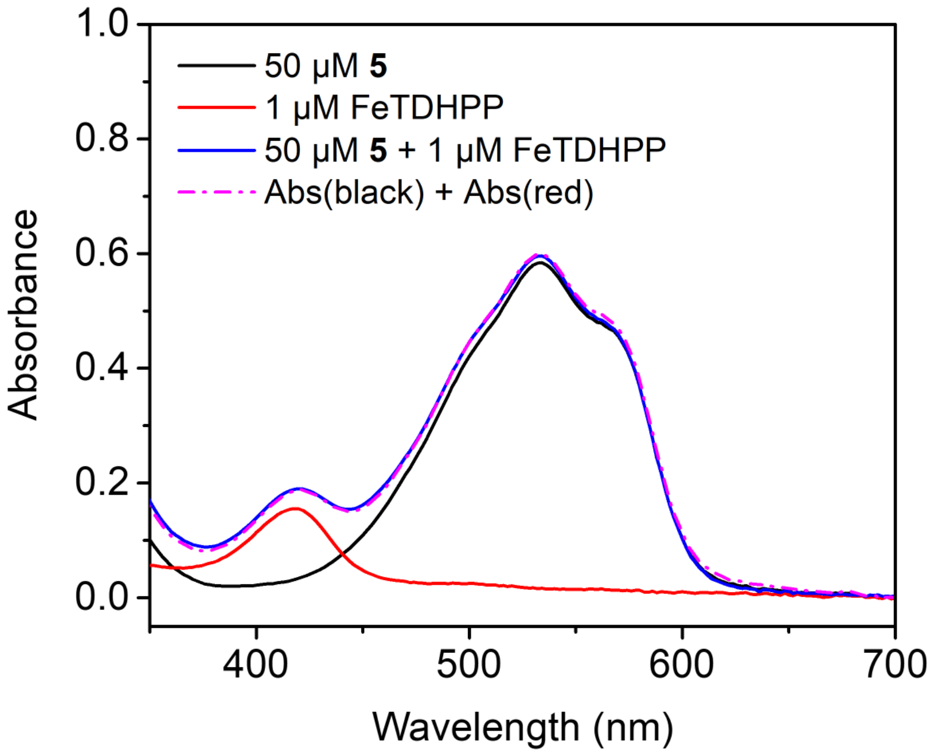

Supplementary Figure 29. UV-vis absorption spectra. UV-vis absorption spectra of 50 µM **5** (black), 1 µM FeTDHPP (red), and a mixture of 50 µM **5** and 1 µM FeTDHPP (blue) in DMF in a quartz cuvette (10–mm path length) under CO_2 at 298 K. Source data are provided as a Source Data file.

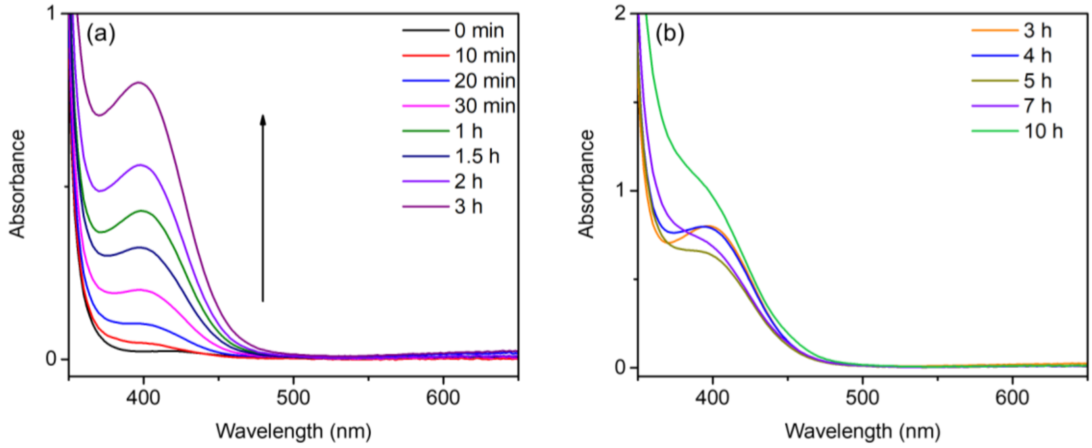

Supplementary Figure 30. UV-vis absorption spectra. UV-vis absorption spectra of systems containing 10 mM BIH, 1 µM FeTDHPP, and 20 µM AQ in CO_2-saturated DMF in a quartz cuvette (10–mm path length) at 298 K under irradiation with a white LED (λ > 400 nm, 100 mW/cm^2). Irradiation time ranging from 0 to 3 h (**a**), and from 3 h to 10 h (**b**).

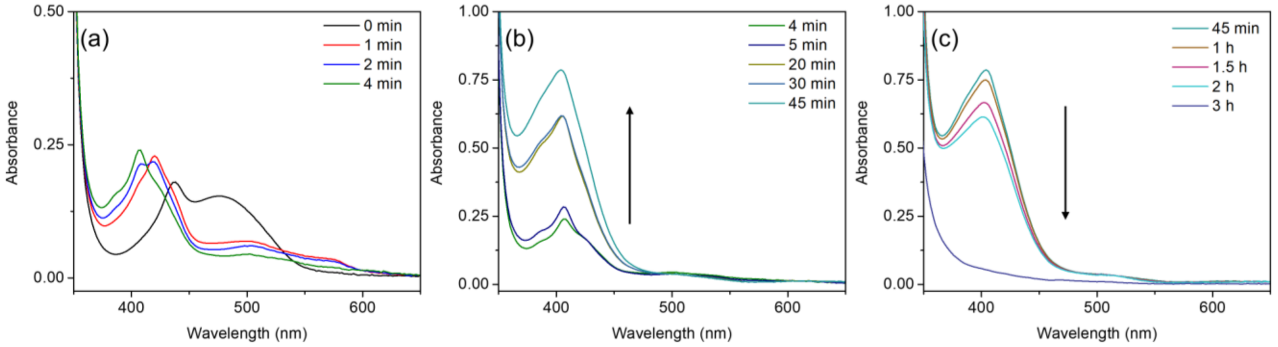

Supplementary Figure 31. UV-vis absorption spectra. UV-vis absorption spectra of systems containing 10 mM BIH, 1 μM FeTDHPP, and 20 μM **1** in CO_2-saturated DMF in a quartz cuvette (10–mm path length) at 298 K under irradiation with a white LED (λ > 400 nm, 100 mW/cm^2). Irradiation time ranging from 0 to 4 min (**a**), from 4 to 45 min (**b**), and from 45 min to 3 h (**c**).

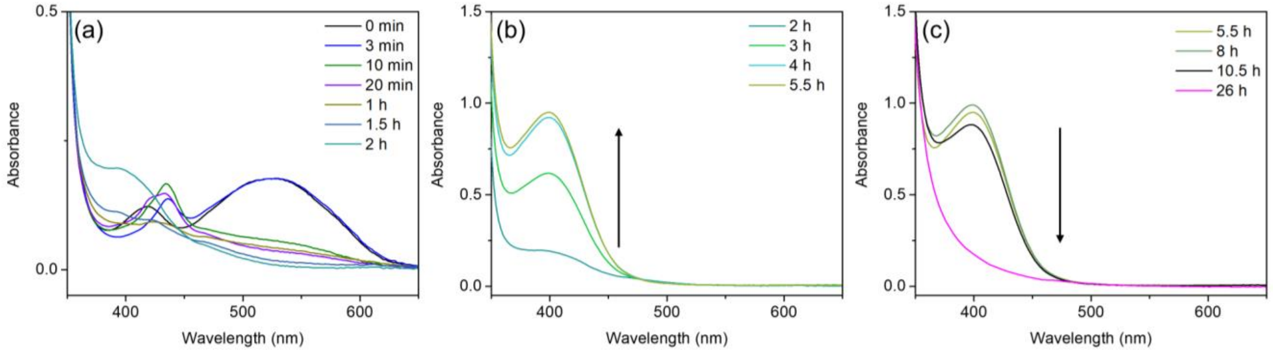

Supplementary Figure 32. UV-vis absorption spectra. UV-vis absorption spectra of systems containing 10 mM BIH, 1 μM FeTDHPP and 20 μM **2** in CO_2-saturated DMF in a quartz cuvette (10–mm path length) at 298 K under irradiation with a white LED (λ > 400 nm, 100 mW/cm^2). Irradiation time ranging from 0 to 2 h (**a**), from 2 to 5.5 h (**b**), and from 5.5 to 26 h (**c**).

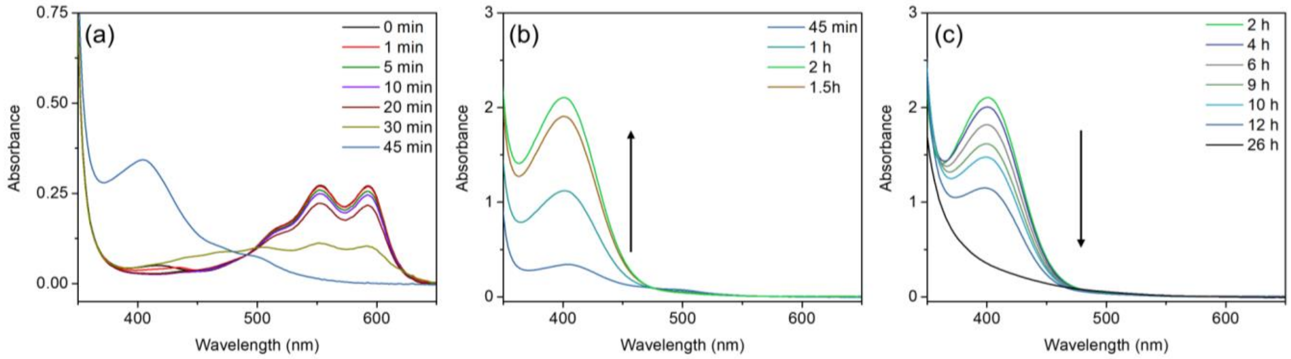

Supplementary Figure 33. UV-vis absorption spectra. UV-vis absorption spectra of systems containing 10 mM BIH, 1 μM FeTDHPP and 20 μM **3** in CO_2-saturated DMF in a quartz cuvette (10–mm path length) at 298 K under irradiation with a white LED (λ > 400 nm, 100 mW/cm^2). Irradiation time ranging from 0 to 45 min (**a**), from 45 min to 2 h (**b**), and from 2 to 26 h (**c**).

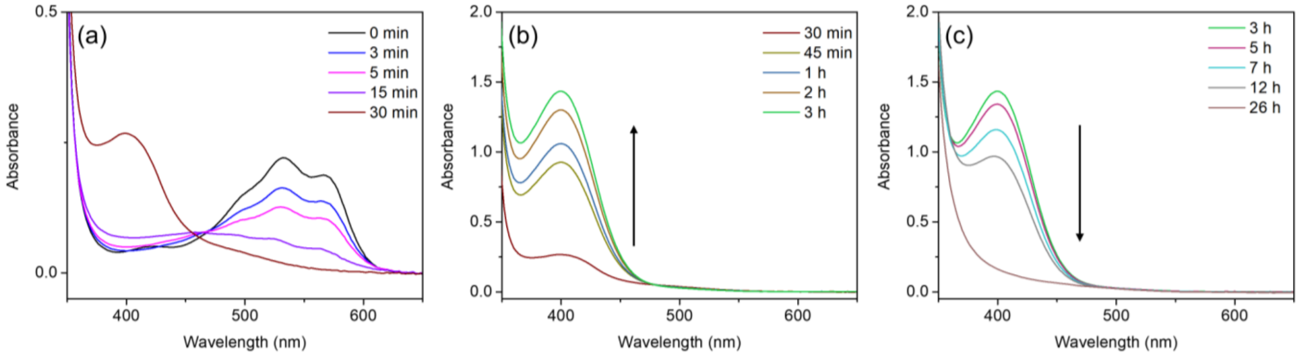

Supplementary Figure 34. UV-vis absorption spectra. UV-vis absorption spectra of systems containing 10 mM BIH, 1 μM FeTDHPP and 20 μM **4** in CO_2-saturated DMF in a quartz cuvette (10–mm path length) at 298 K under irradiation with a white LED (λ > 400 nm, 100 mW/cm^2). Irradiation time ranging from 0 to 30 min (**a**), from 30 min to 3 h (**b**), and from 3 to 26 h (**c**).

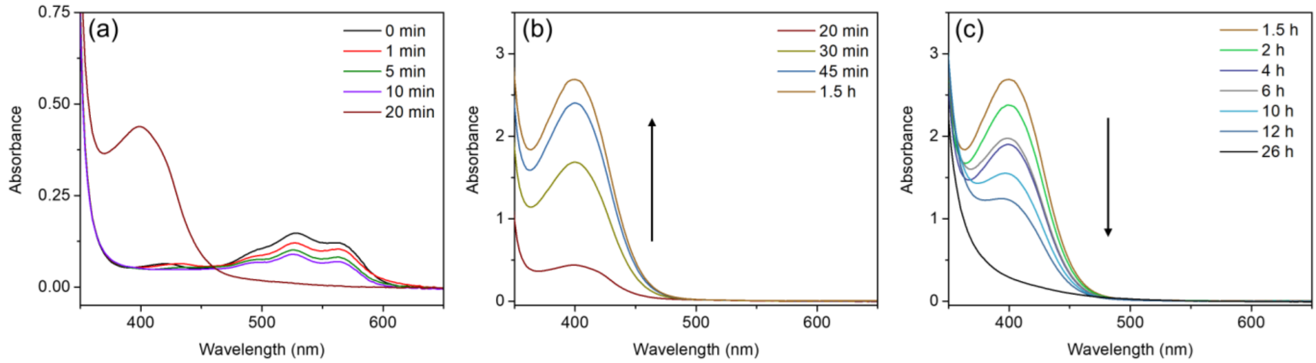

Supplementary Figure 35. UV-vis absorption spectra. UV-vis absorption spectra of systems containing 10 mM BIH, 1 μM FeTDHPP and 20 μM **5** in CO_2-saturated DMF in a quartz cuvette (10–mm path length) at 298 K under irradiation with a white LED (λ > 400 nm, 100 mW/cm^2). Irradiation time ranging from 0 to 20 min (**a**), from 20 min to 1.5 h (**b**), and from 1.5 to 26 h (**c**).

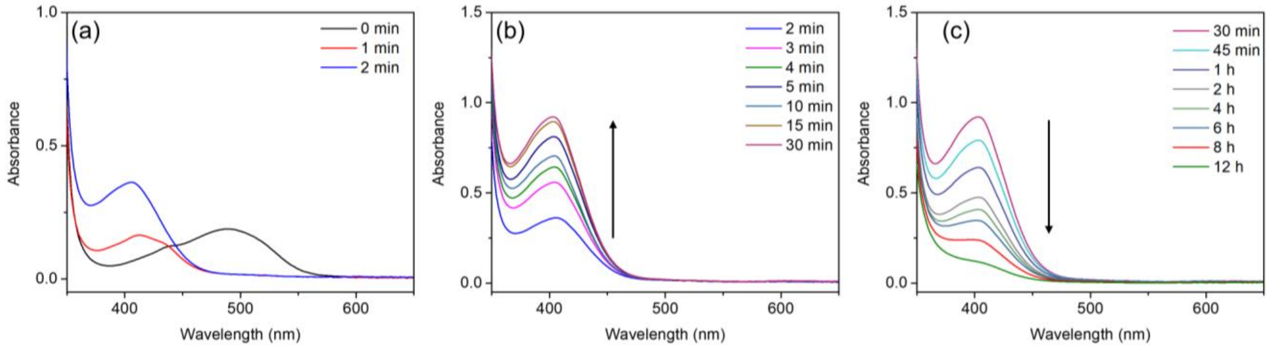

Supplementary Figure 36. UV-vis absorption spectra. UV-vis absorption spectra of systems containing 10 mM BIH, 1 μM FeTDHPP and 20 μM **6** in CO_2-saturated DMF in a quartz cuvette (10–mm path length) at 298 K under irradiation with a white LED (λ > 400 nm, 100 mW/cm^2). Irradiation time ranging from 0 to 2 min (**a**), from 2 to 30 min (**b**), and from 30 min to 12 h (**c**).

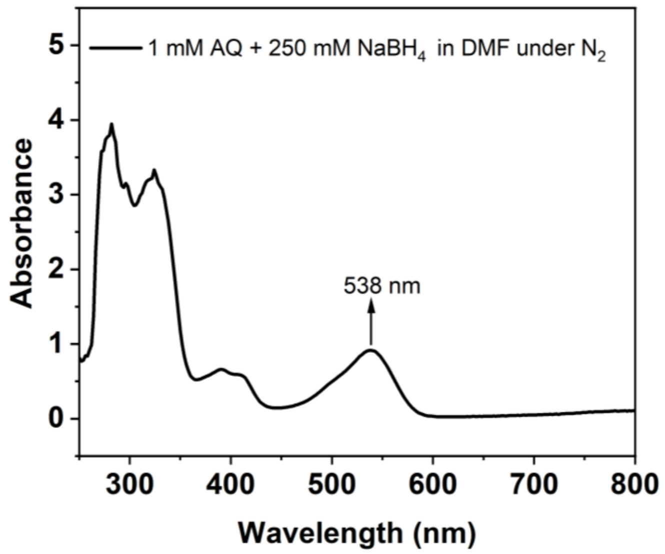

Supplementary Figure 37. UV-vis absorption spectrum. UV-vis absorption spectrum of a system containing AQ (1.0 mM) and $NaBH_4$ (250 mM) in DMF for 30 min under N_2 at 298 K.

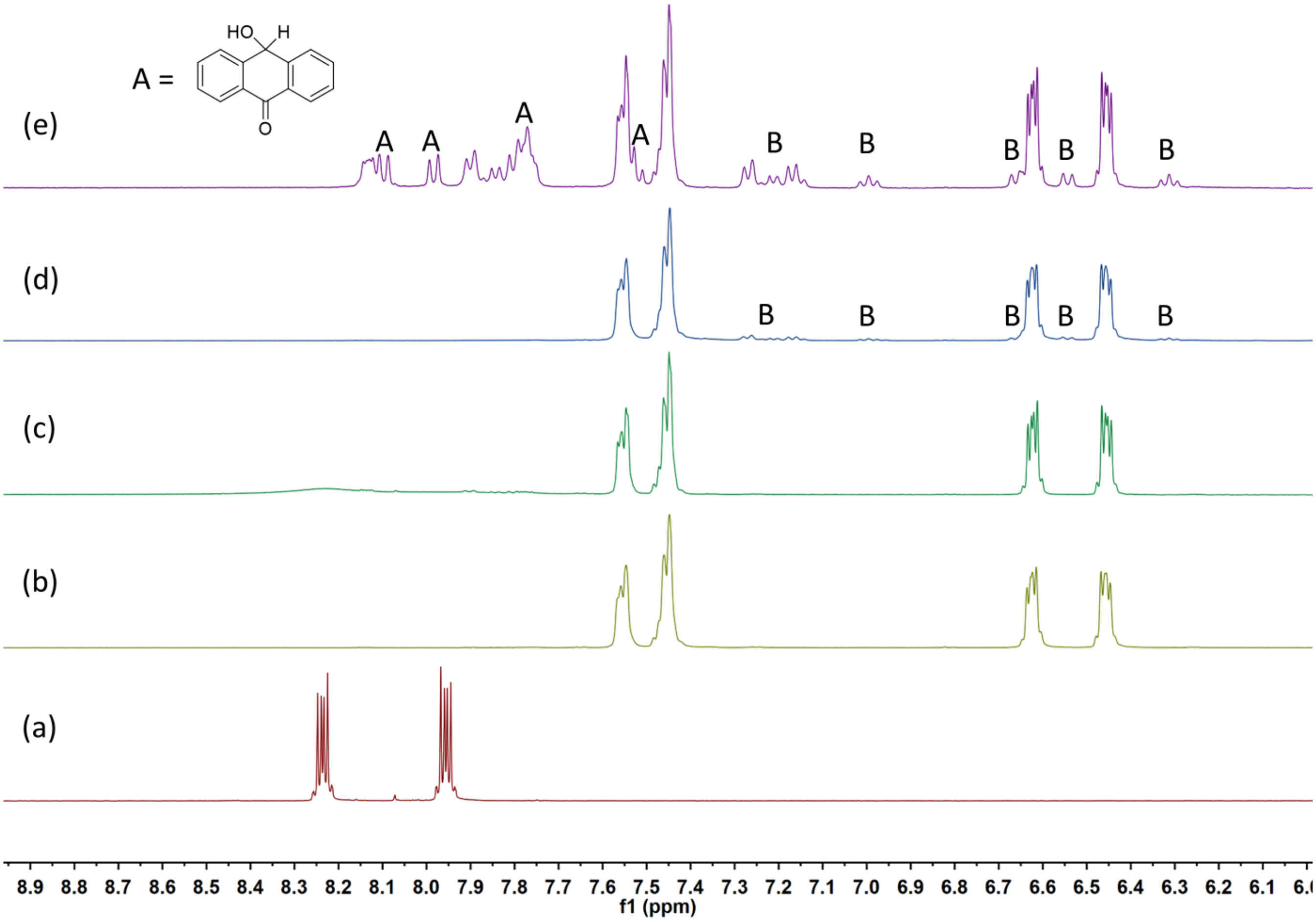

Supplementary Figure 38. ^{1}H NMR spectra in DMSO-d_6. (**a**) AQ. (**b**) BIH. (**c**) A mixture of AQ and 2.0 equiv of BIH. (**d**) BIH irradiated for 21 h with a white LED (λ > 400 nm, 100 mW/cm^2). (**e**) A mixture of AQ and 2.0 equiv of BIH irradiated for 22 h with a white LED (λ > 400 nm, 100 mW/cm^2); products: 10-hydroxyanthrone (**A**),[1] and species (**B**) attributed to the decomposition of BIH.

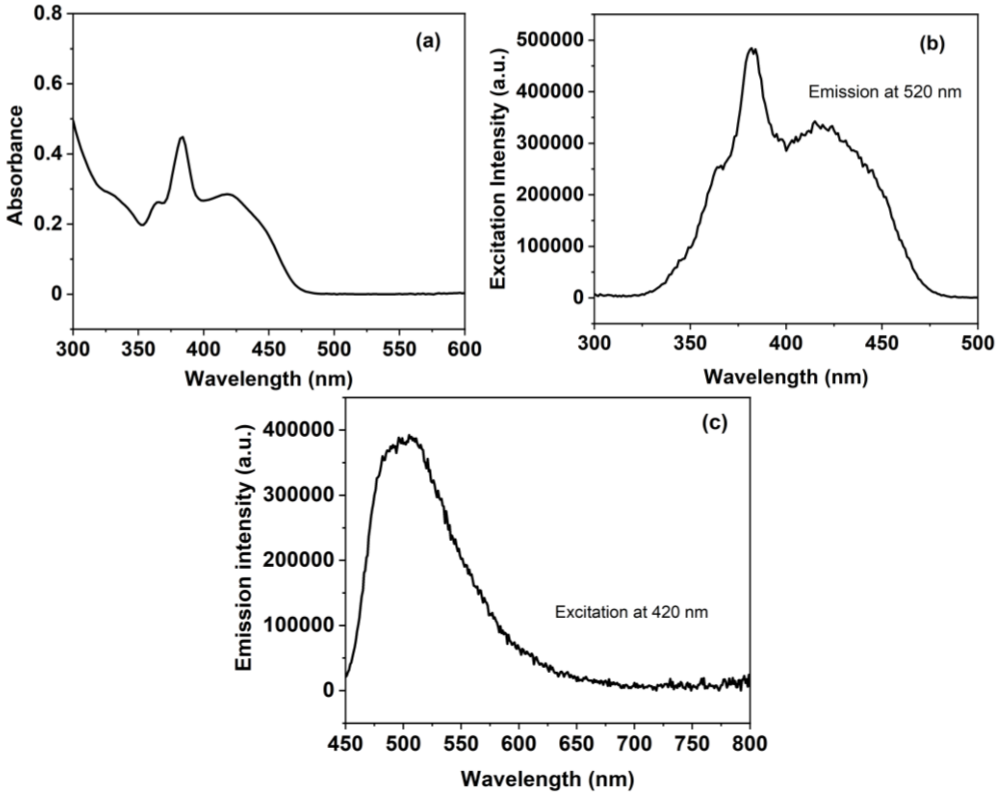

Supplementary Figure S39. Spectra of AQH_2. UV-vis spectrum (**a**), excitation spectrum (**b**) and emission spectrum (**c**) of AQH_2 in DMSO-d_6 in a quartz cuvette (10–mm path length). Similar results have been previously reported.[1] The AQH_2 species was generated from a procedure as follows: A DMSO-d_6 solution (1.0 mL) containing 0.1 mmol AQ was added to $NaBH_4$ (0.15 mmol) under N_2. The reaction mixture was allowed to stir for 6 h at room temperature under N_2. Then 1000 eq. CH_3COOH was added to generated the AQH_2. The spectra were recorded in a diluted solution (dilution factor of 2000) under N_2.

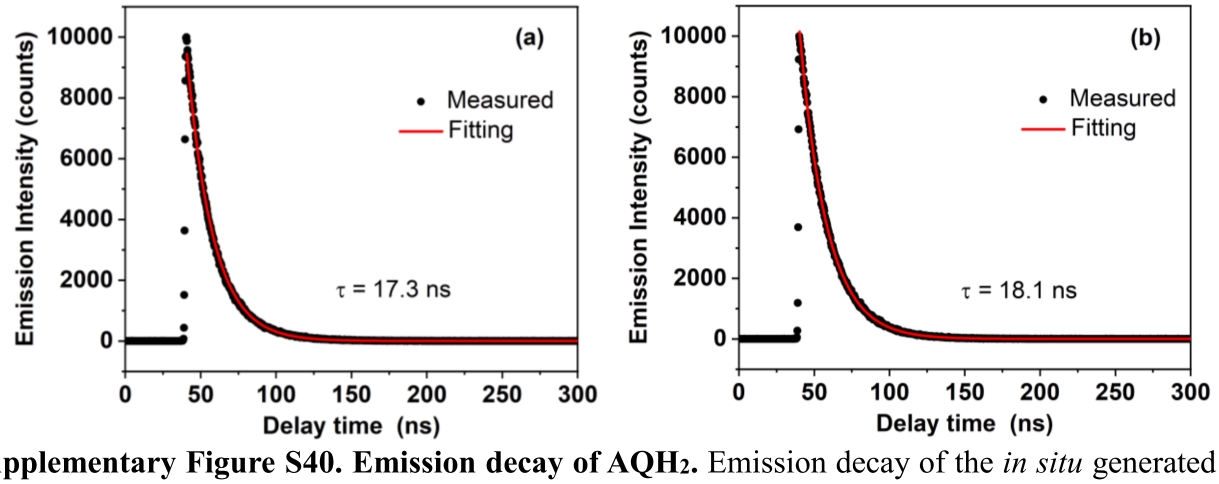

Supplementary Figure S40. Emission decay of AQH_2. Emission decay of the *in situ* generated AQH_2 in DMF (**a**) or DMSO-d_6 (**b**) in a quartz cuvette (10–mm path length) under N_2 at 298 K. The lines were fitted with a single exponential. The AQH_2 species was generated from a procedure as follows: A DMSO-d_6 solution (1.0 mL) containing 0.1 mmol AQ was added to $NaBH_4$ (0.15 mmol) under N_2. The reaction mixture was allowed to stir for 6 h at room temperature under N_2. Then 1000 eq. CH_3COOH was added to generated the AQH_2. The spectra were recorded in a diluted solution (dilution factor of 2000) under N_2.

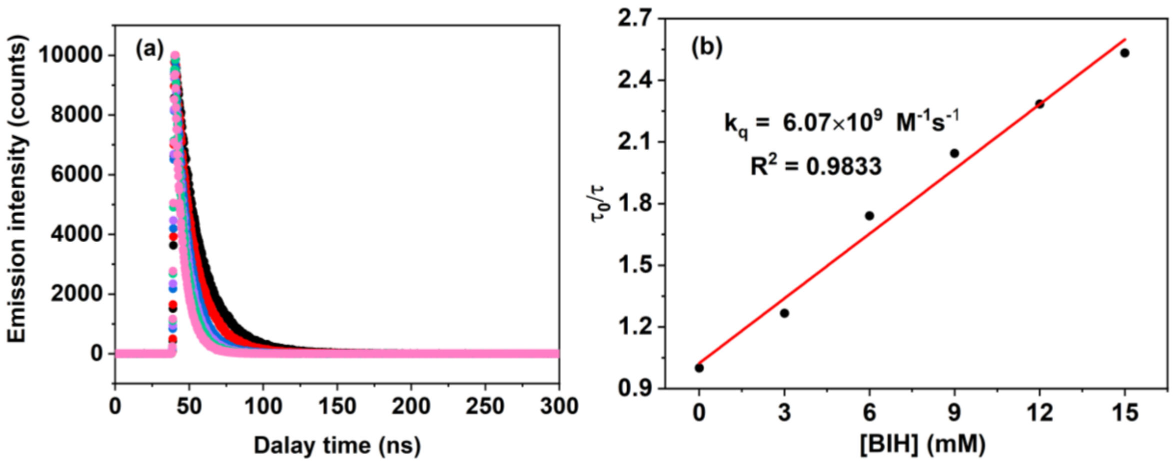

Supplementary Figure S41. Luminescence lifetime quenching of AQH₂. Emission decay of the *in situ* generated AQH_2 with addition of different concentrations of BIH in DMF in a quartz cuvette (10–mm path length) under N_2 at 298 K, with lines fitted with a single exponential (**a**). A Stern-Volmer plot showing a quenching rate constant of 6.07×10^9 $M^{-1} \cdot s^{-1}$ (**b**). The excitation wavelength was 406.5 nm. The AQH_2 species was generated from a procedure as follows: A DMSO-d_6 solution (1.0 mL) containing 0.1 mmol AQ was added to $NaBH_4$ (0.15 mmol) under N_2. The reaction mixture was allowed to stir for 6 h at room temperature under N_2. Then 1000 eq. CH_3COOH was added to generated the AQH_2. The spectra were recorded in a diluted solution (dilution factor of 2000) under N_2.

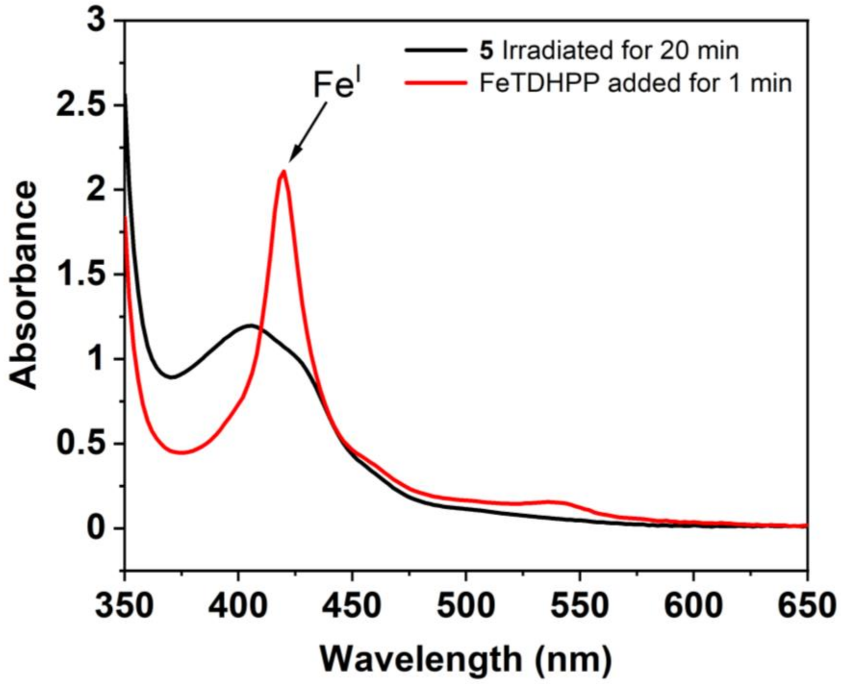

Supplementary Figure S42. UV-vis absorption spectra. UV-vis absorption spectra of systems containing PS **5** (40 μM) and BIH (20 mM) in DMF (2 mL) in a quartz cuvette (10–mm path length) under CO_2 upon irradiation with white LED light (λ > 400 nm, 100 mW/cm^2) for 20 min (black), and then added with FeTDHPP (0.25 equiv vs **5**) for 1 min (red). The amount of CO quantitated by GC was 0.027 ± 0.001 μmol after addition of FeTDHPP.

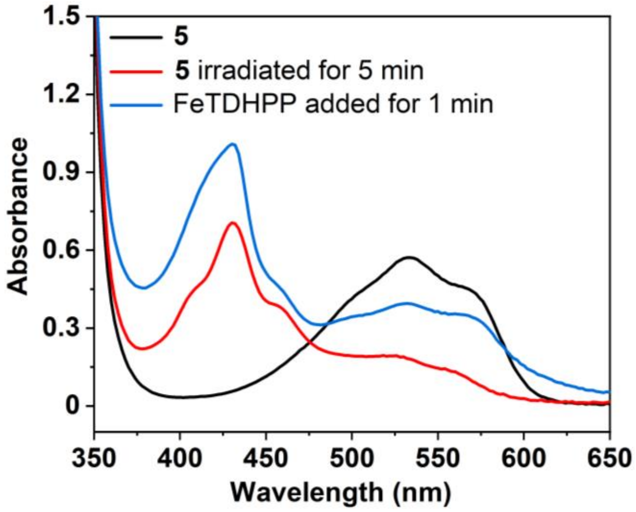

Supplementary Figure S43. UV-vis absorption spectra. UV-vis absorption spectra of systems containing PS **5** (40 μM) and BIH (20 mM) in DMF (2 mL) in a quartz cuvette (10–mm path length) under CO_2 before (black) and after irradiation with white LED light (λ > 400 nm, 100 mW/cm^2) for 5 minutes (red), and then added with FeTDHPP (0.25 equiv vs **5**) for 1 min (blue). No CO was detected after adding FeTDHPP by GC analysis.

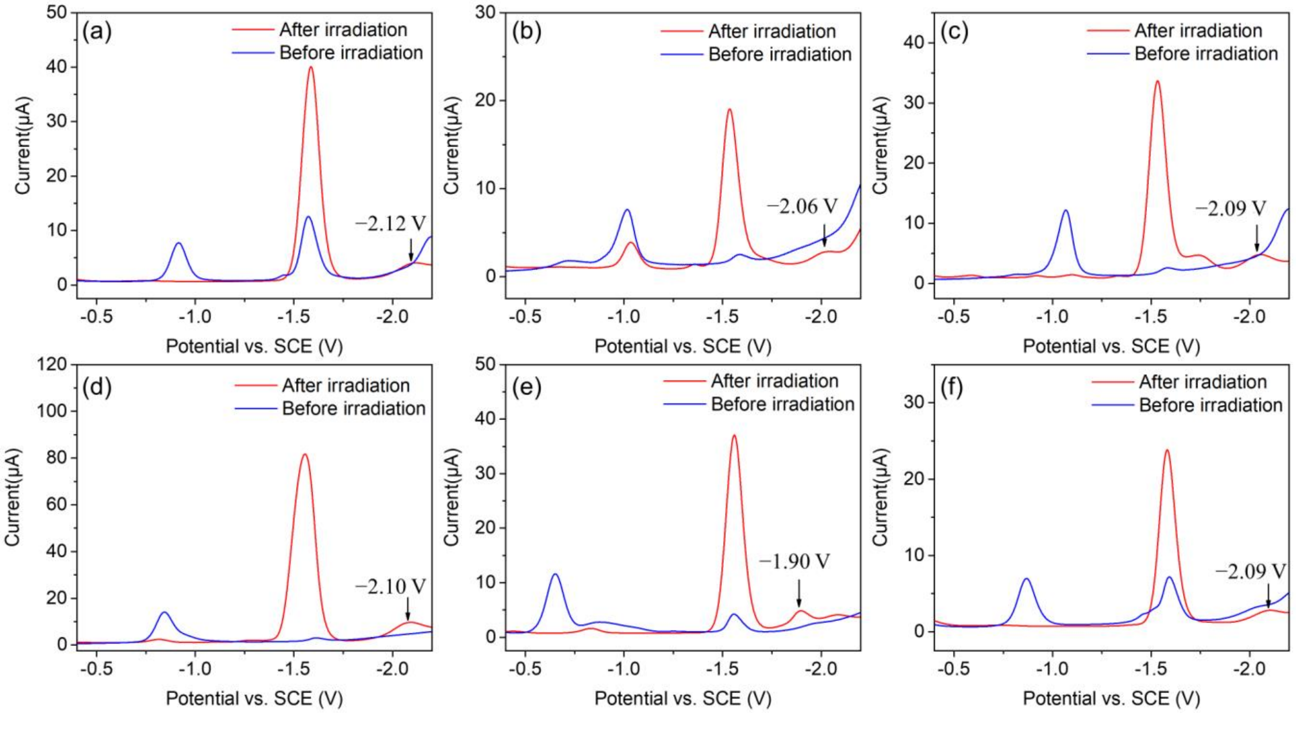

Supplementary Figure 44. Electrochemical study. SWVs of 0.3 mM (a) **1**; (b) **2**; (c) **3**; (d) **4**; (e) **5**; and (f) **6** with 10 mM BIH in CO_2-saturated 0.1 M $TBAPF_6$ DMF solutions before and after irradiation with a white LED (λ > 400 nm, 100 mW/cm^2) until the solution turning yellow. The peak at -1.6 V is consistent with a BI^+ species. The experiments were conducted using a glassy carbon working electrode (3.0 mm in diameter), a Pt wire counter electrode, and a saturated calomel electrode (SCE) at a scan rate of 100 mV·s^{-1}. Source data are provided as a Source Data file.

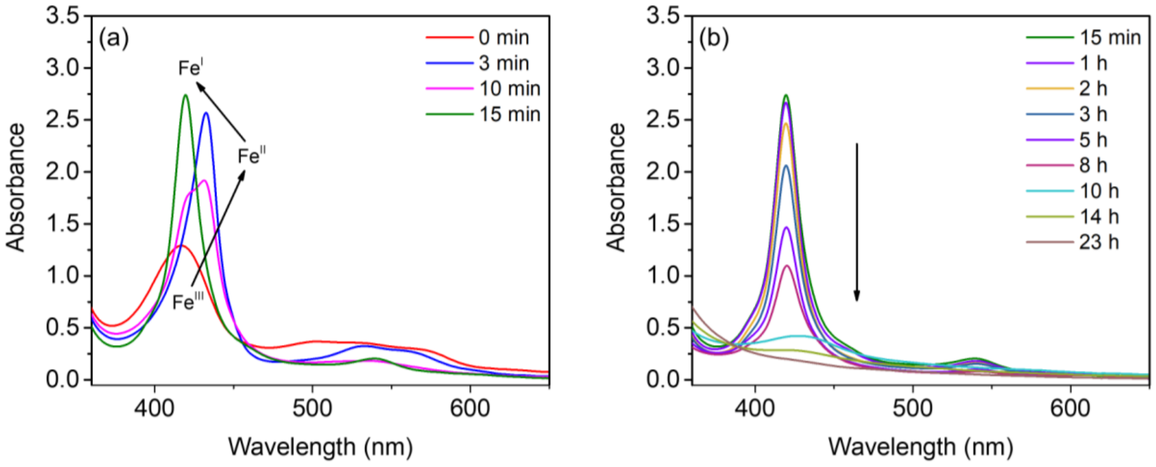

Supplementary Figure 45. UV-vis absorption spectra. UV-vis absorption spectra of systems containing 30 mM BIH, 20 μM FeTDHPP, and 20 μM **5** in CO_2-saturated DMF in a quartz cuvette (10–mm path length) at 298 K under irradiation with a white LED (λ > 400 nm, 100 mW/cm^2). Irradiation time ranging from 0 to 15 min (**a**), and from 15 min to 23 h (**b**).

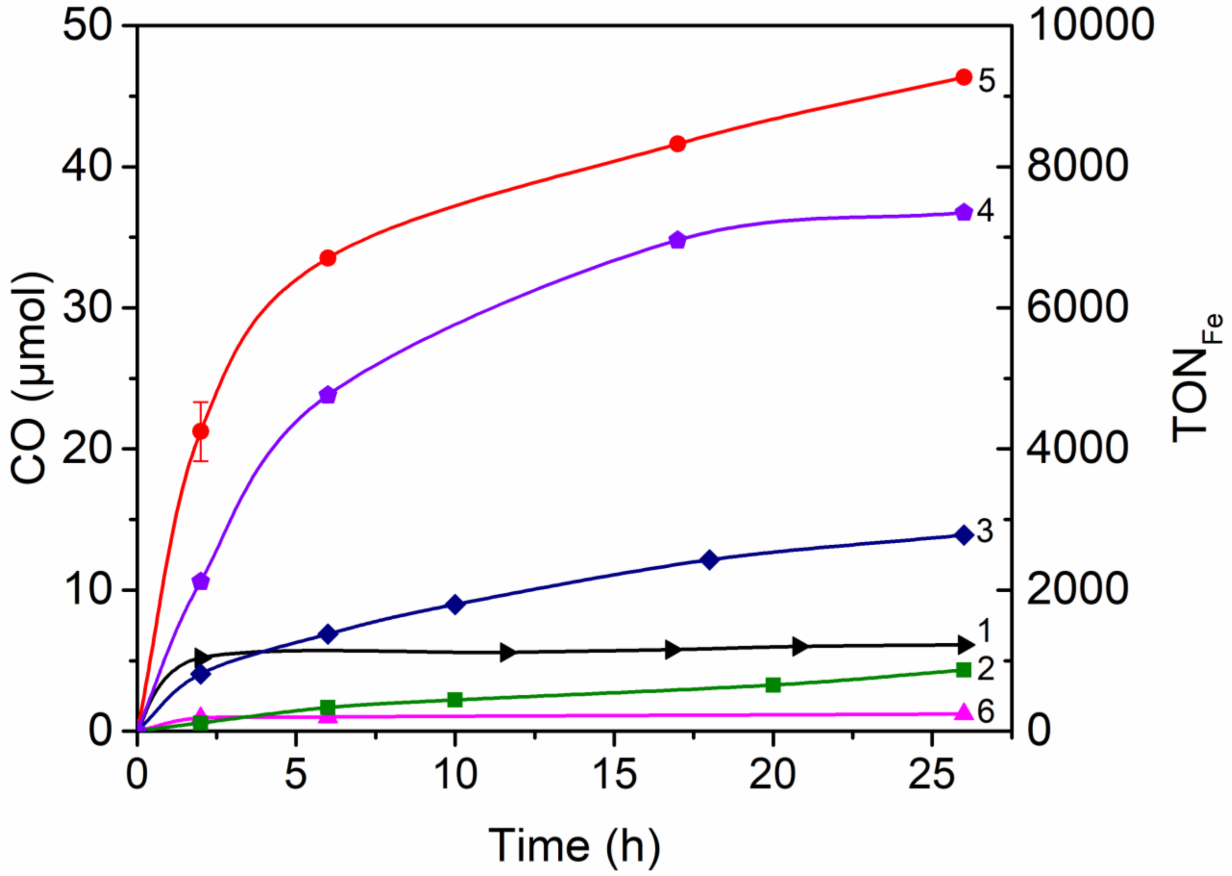

Supplementary Figure 46. Photocatalytic CO_2 reduction. Experiments were performed in 5.0 mLCO_2-saturated DMF solution containing 10 mM BIH, 1.0 μM FeTDHPP, and 20 μM PSs **1**–**6** under irradiation with white LED light (λ > 400 nm, 100 mW/cm^2) at 298 K. The error bar denotes standard deviation, based on 3 separated runs. Source data are provided as a Source Data file.

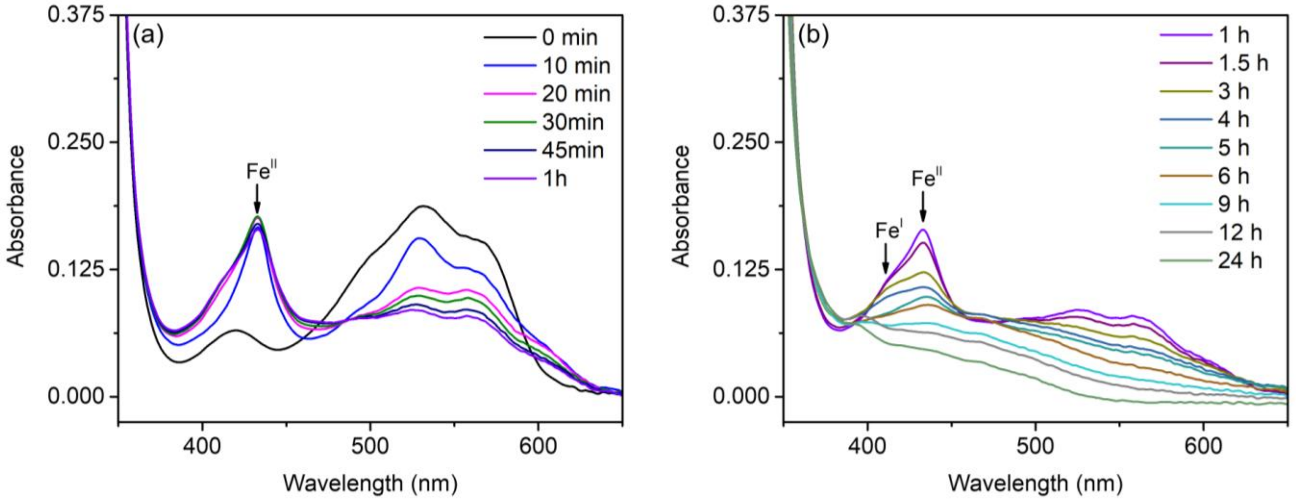

Supplementary Figure 47. UV-vis absorption spectra. UV-vis absorption spectra of systems containing 10 mM BIH, 1.0 μM FeTDHPP, and 20 μM **5** in CO_2-saturated DMF in a quartz cuvette (10–mm path length) at 298 K under irradiation with a 300 W Xe lamp equipped with a 550 nm cut-off filter (λ > 550 nm). Irradiation time ranging from 0 to 1 h (**a**), and from 1 to 24 h (**b**).

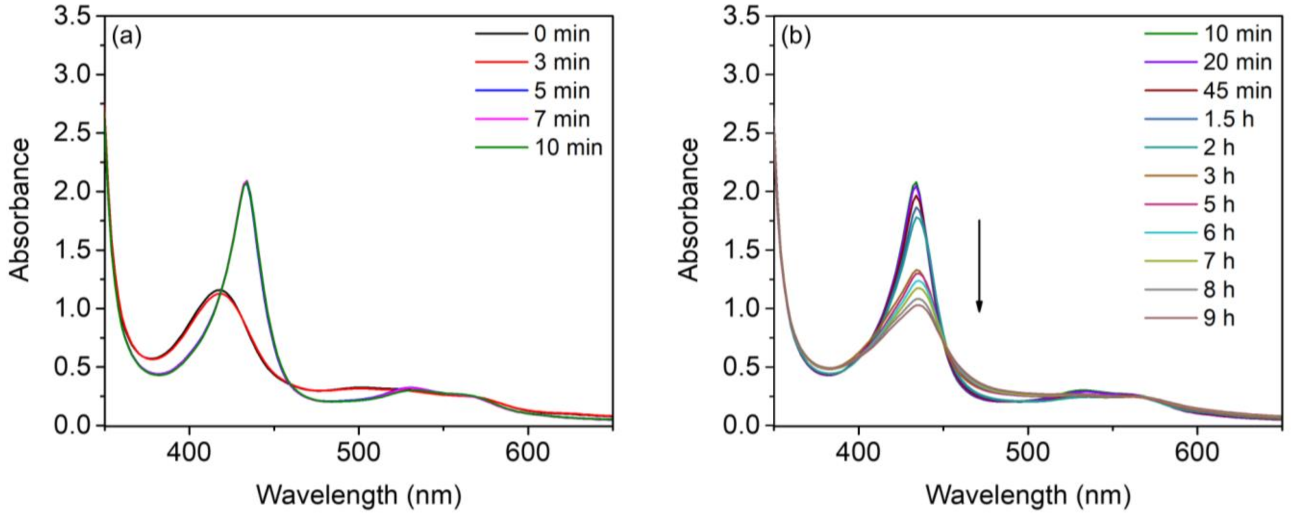

Supplementary Figure 48. UV-vis absorption spectra. UV-vis absorption spectra of systems containing 30 mM BIH, 20 μM FeTDHPP, and 20 μM **5** in CO_2-saturated DMF in a quartz cuvette (10–mm path length) at 298 K under irradiation with a 300 W Xe lamp equipped with a 550 nm cut-off filter (λ > 550 nm). Irradiation time ranging from 0 to 10 min (**a**), and from 10 min to 9 h (**b**).

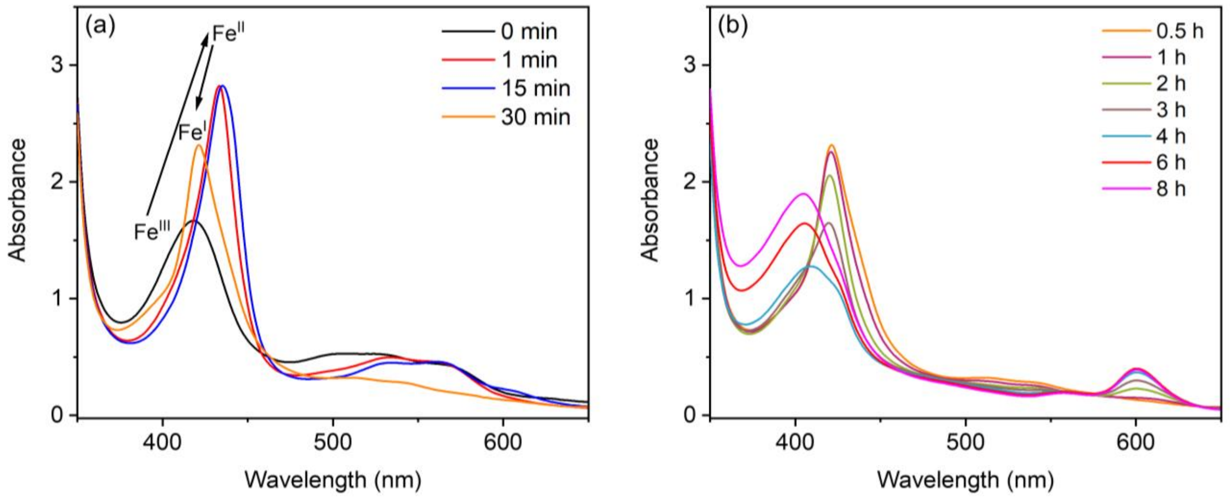

Supplementary Figure 49. UV-vis absorption spectra. Systems containing 30 mM BIH, 20 μM FeTDHPP, and 20 μM **5** in CO_2-saturated DMF in a quartz cuvette (10–mm path length) at 298 K under irradiation with a blue LED (λ = 450 nm, ΔP·λ = 12300 mW·nm/cm^2). Irradiation time ranging from 0 to 30 min (**a**), and from 30 min to 8 h (**b**).

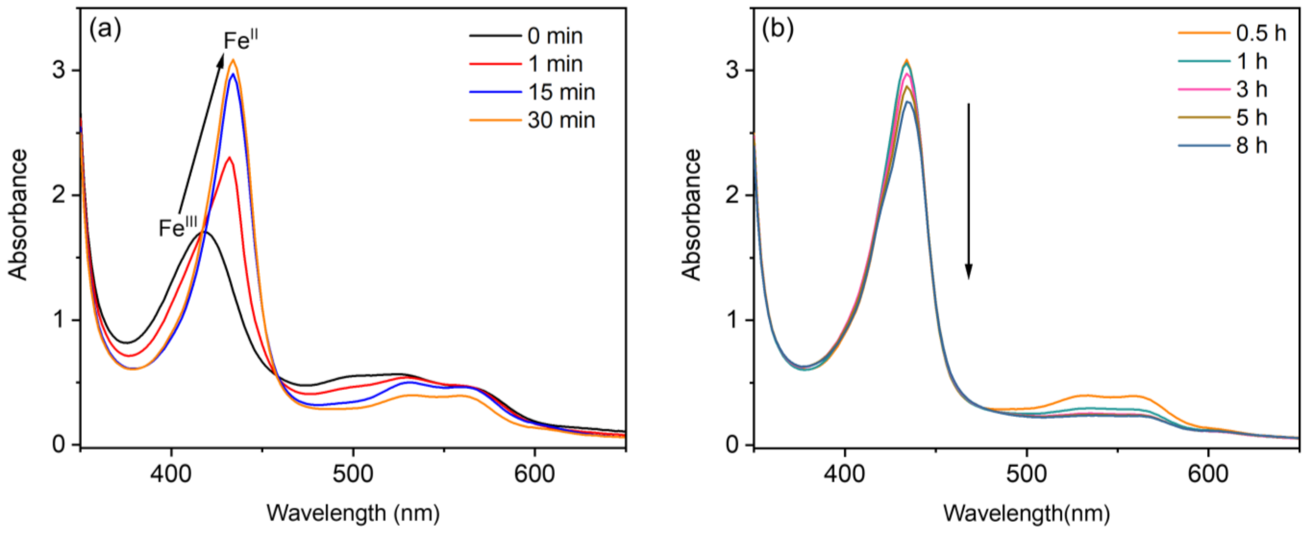

Supplementary Figure 50. UV-vis absorption spectra. Systems containing 30 mM BIH, 20 μM FeTDHPP, and 20 μM **5** in CO_2-saturated DMF in a quartz cuvette (10–mm path length) at 298 K under irradiation with a green LED (λ = 525 nm, ΔP·λ = 13203 mW·nm/cm^2). Irradiation time ranging from 0 to 30 min (**a**), and from 30 min to 8 h (**b**).

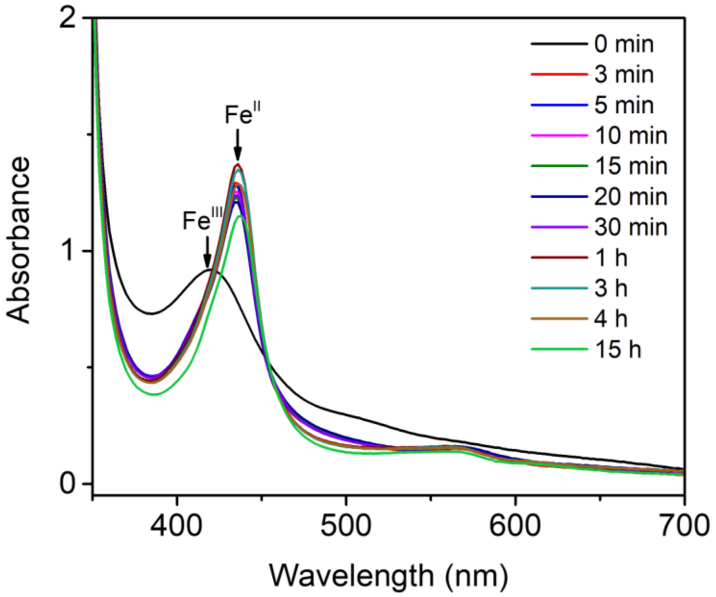

Supplementary Figure 51. UV-vis absorption spectra. UV-vis absorption spectra of systems containing 30 mM BIH and 20 μM FeTDHPP in DMF in a quartz cuvette (10–mm path length) at 298 K under irradiation with a white LED (λ > 400 nm, 100 mW/cm^2) for 15 h.

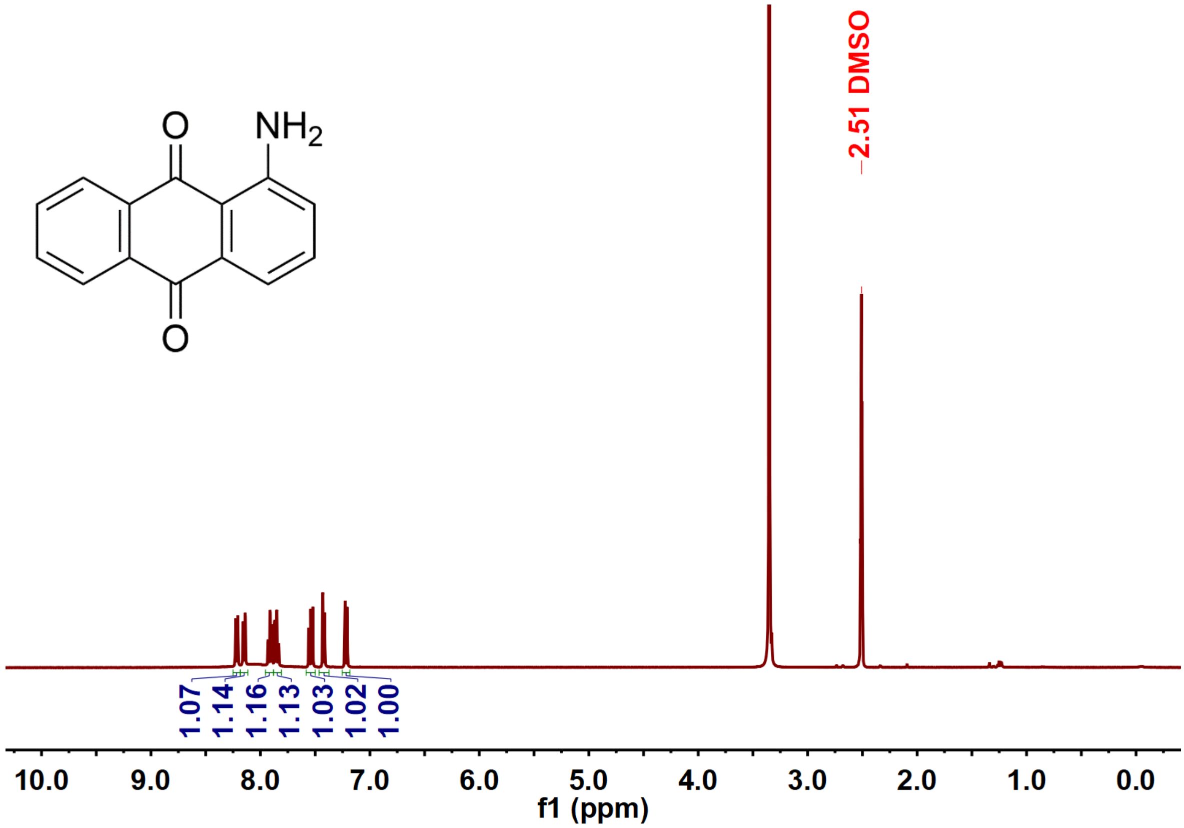

Supplementary Figure 52. ^{1}H NMR spectrum. ^{1}H NMR (400 MHz, 298 K) spectrum of PS **1** in d_6-DMSO.

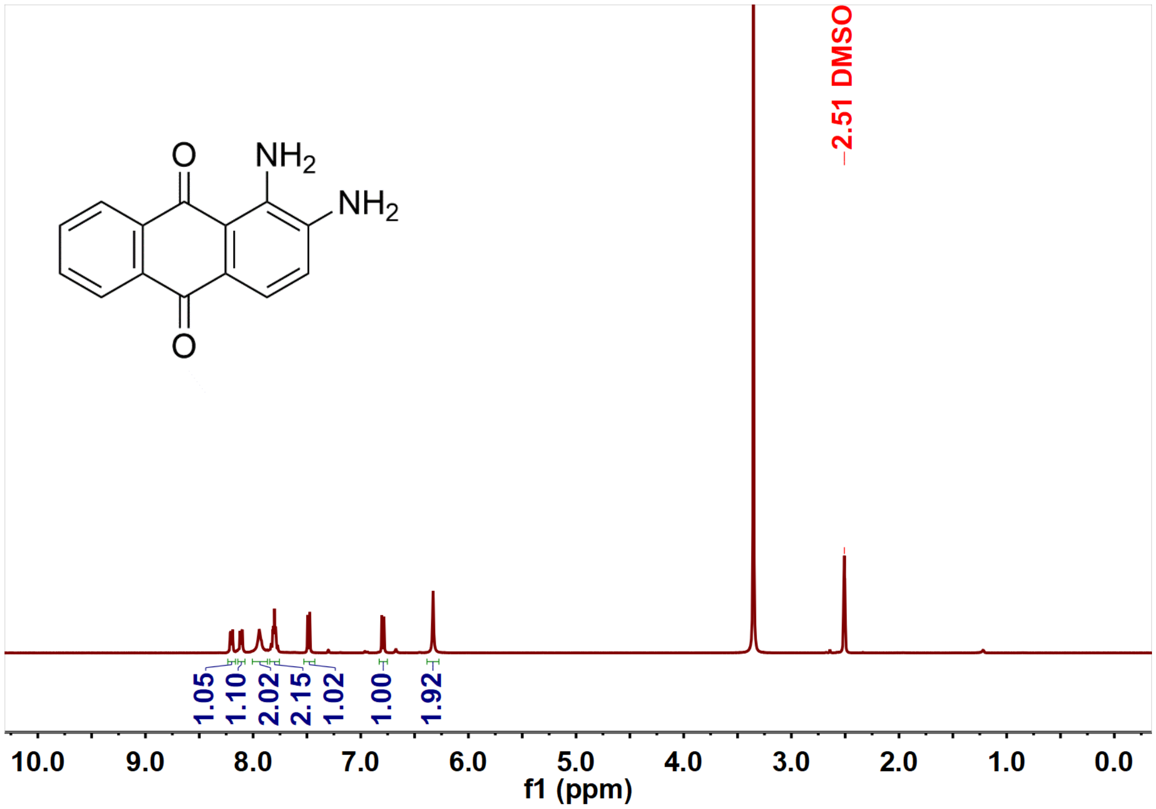

Supplementary Figure 53. ^{1}H NMR spectrum. ^{1}H NMR (400 MHz, 298 K) spectrum of PS **2** in d_6-DMSO.

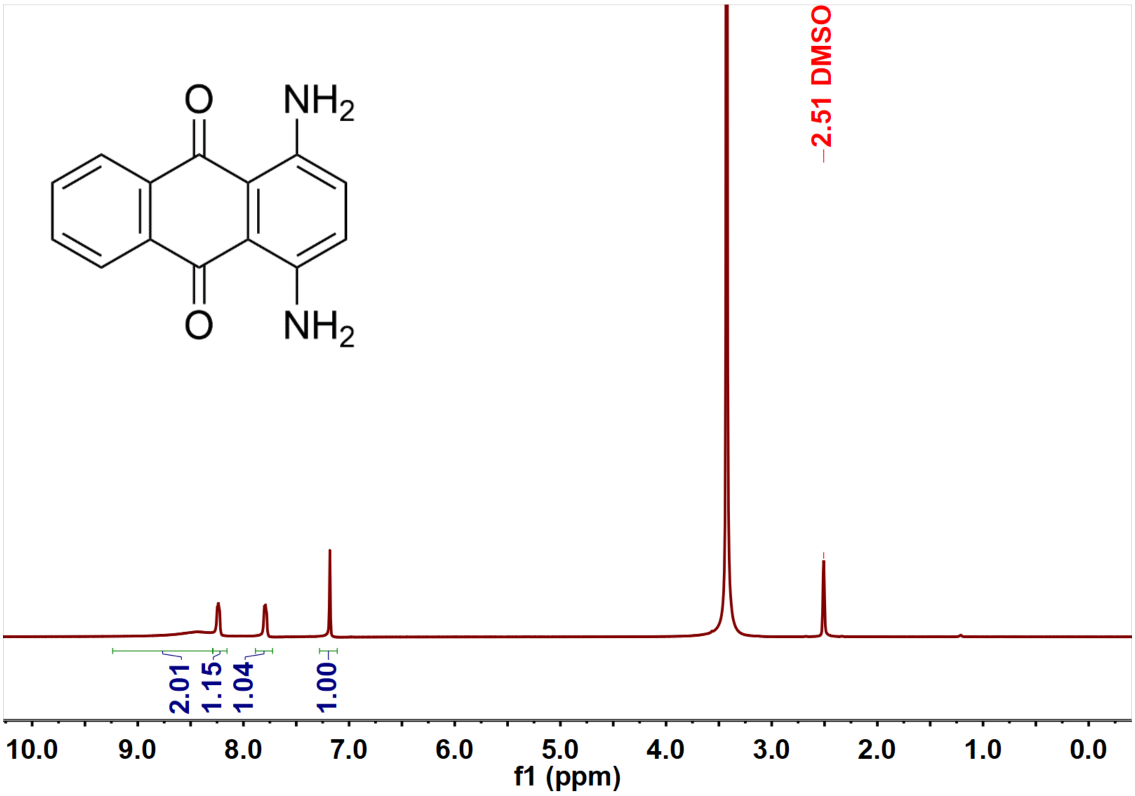

Supplementary Figure 54. ^{1}H NMR spectrum. ^{1}H NMR (400 MHz, 298 K) spectrum of PS **3** in d_6-DMSO.

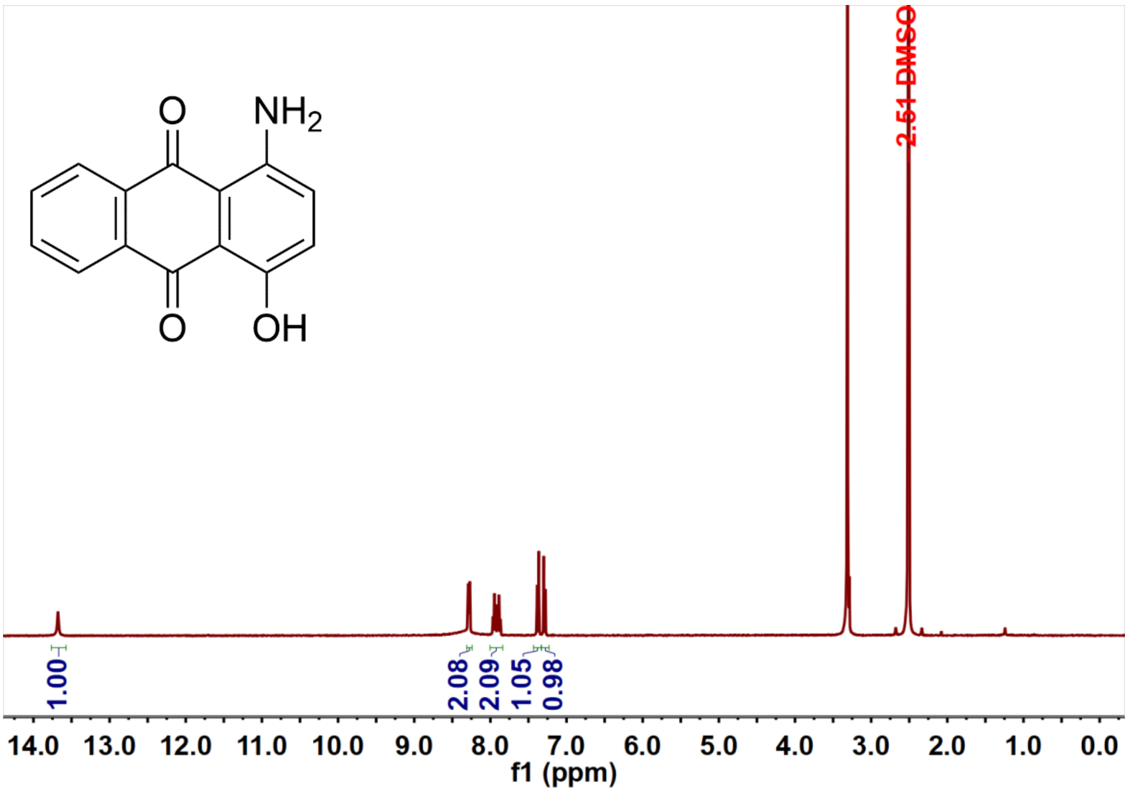

Supplementary Figure 55. ^{1}H NMR spectrum. ^{1}H NMR (400 MHz, 298 K) spectrum of PS **4** in d_6-DMSO.

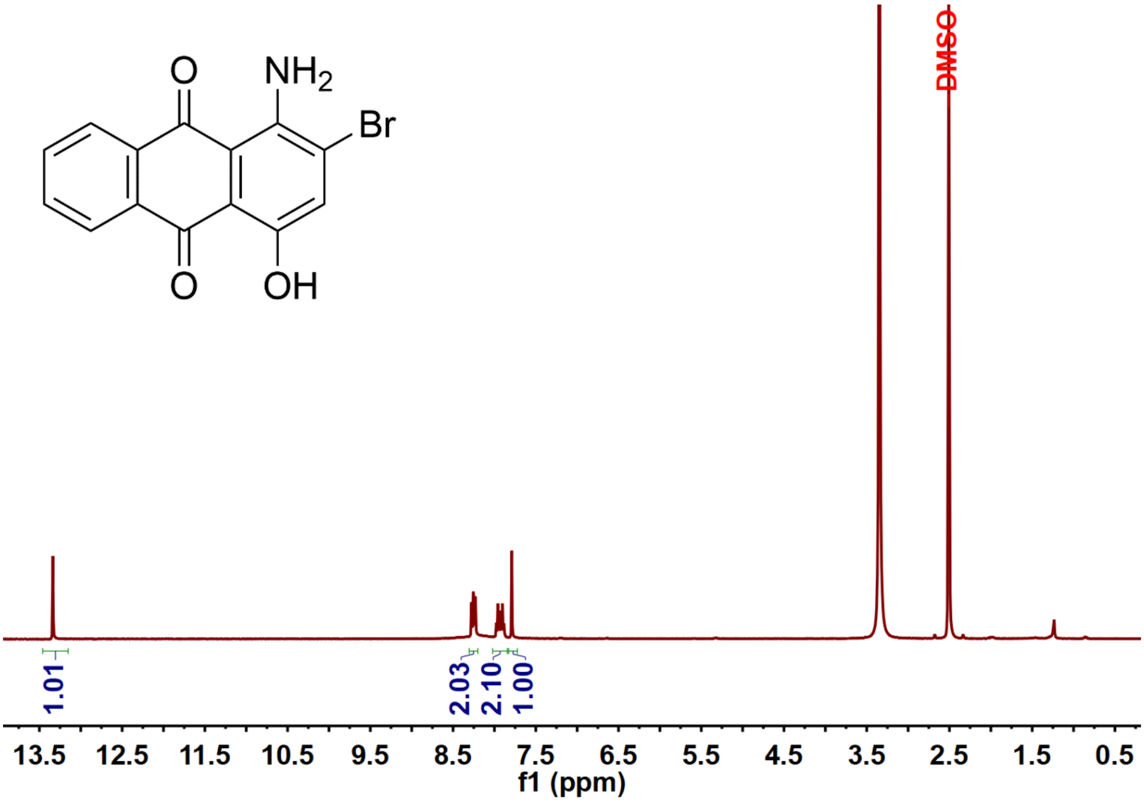

Supplementary Figure 56. ^{1}H NMR spectrum. ^{1}H NMR (400 MHz, 298 K) spectrum of PS **5** in d_6-DMSO.

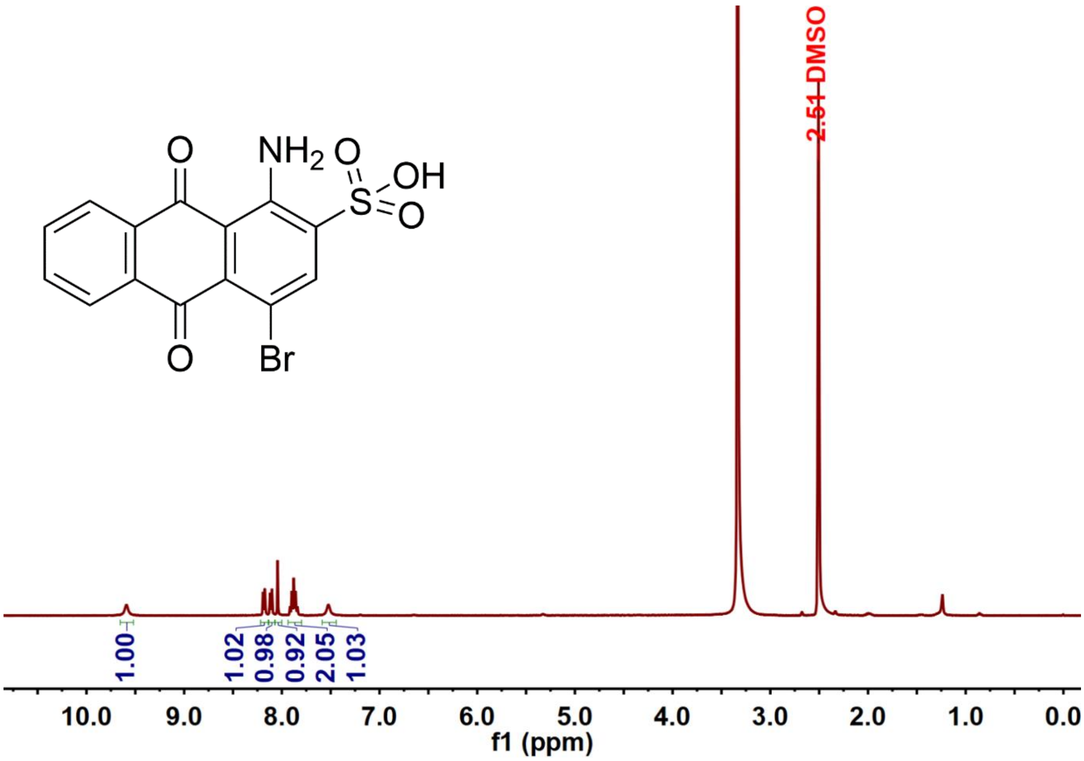

Supplementary Figure 57. ^{1}H NMR spectrum. ^{1}H NMR (400 MHz, 298 K) spectrum of PS **6** in d_6-DMSO.

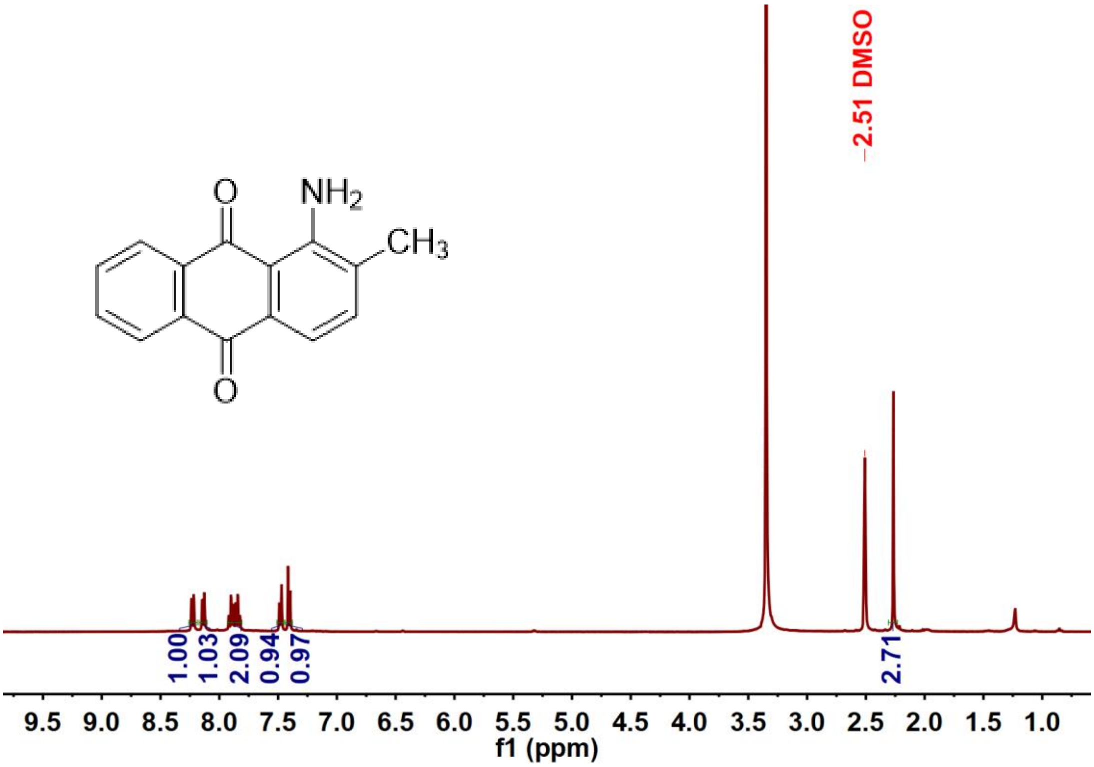

Supplementary Figure 58. ^{1}H NMR spectrum. ^{1}H NMR (400 MHz, 298 K) spectrum of 1-amino-2-methylanthraquinone in d_6-DMSO.

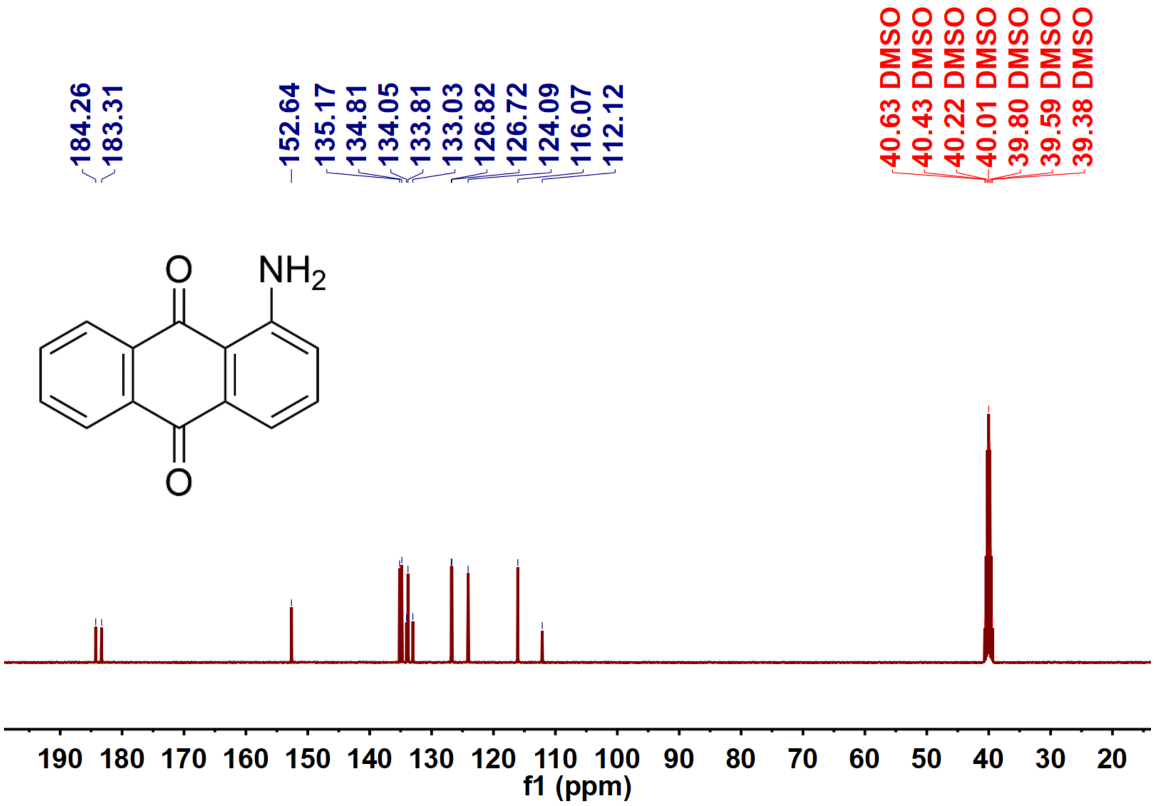

Supplementary Figure 59. ^{13}C NMR spectrum. ^{13}C NMR (100 MHz, 298 K) spectrum of PS **1** in d_6-DMSO.

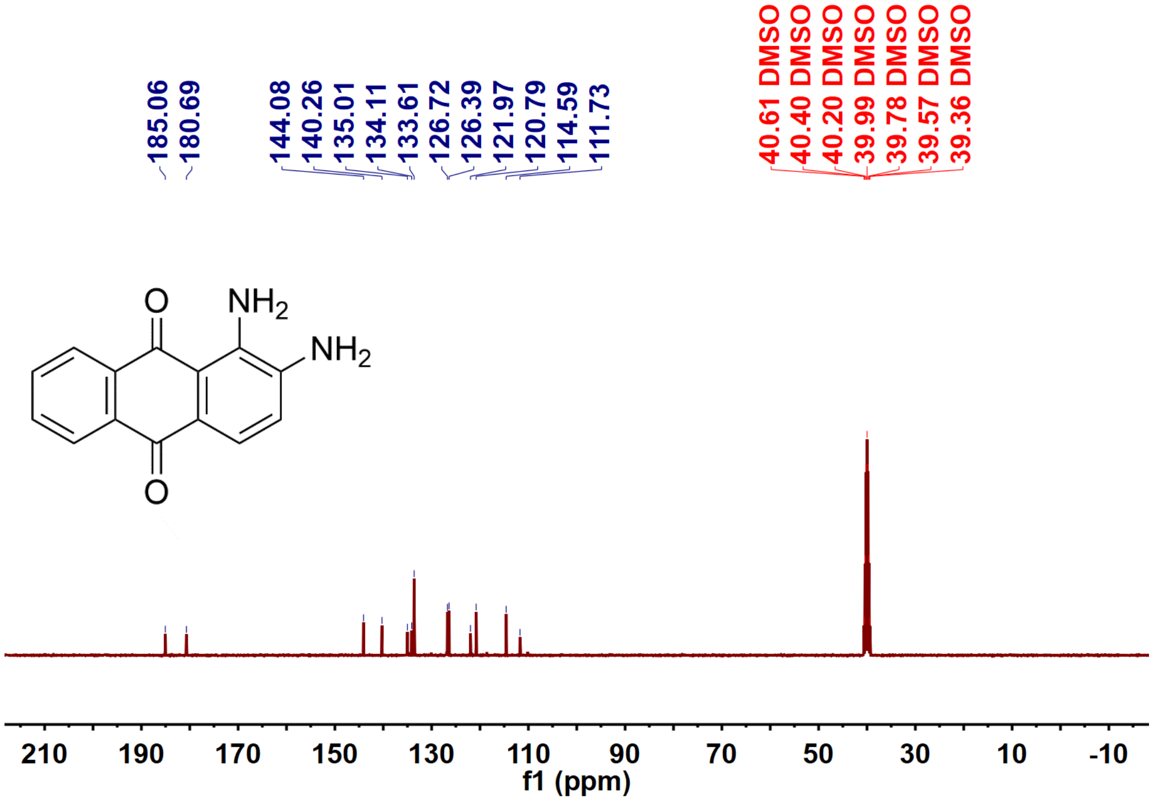

Supplementary Figure 60. ^{13}C NMR spectrum. ^{13}C NMR (100 MHz, 298 K) spectrum of PS **2** in d_6-DMSO.

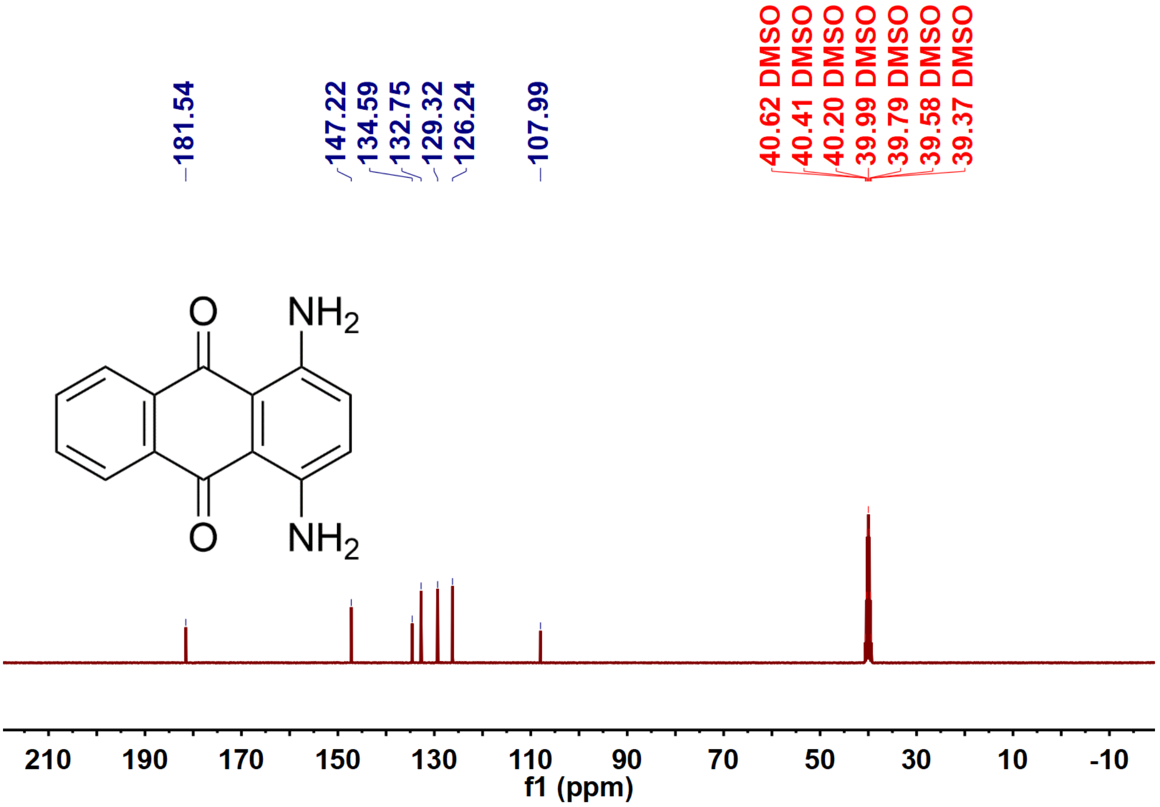

Supplementary Figure 61. ^{13}C NMR spectrum. ^{13}C NMR (100 MHz, 298 K) spectrum of PS **3** in d_6-DMSO.

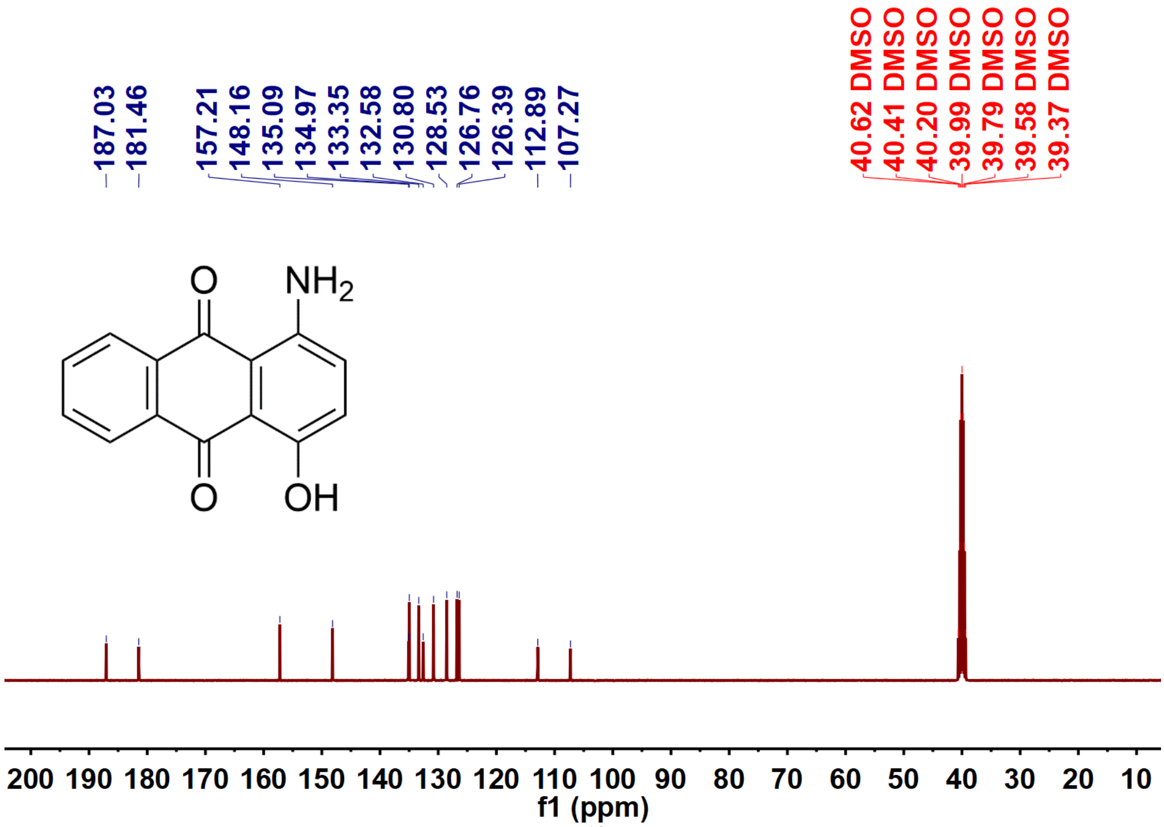

Supplementary Figure 62. ^{13}C NMR spectrum. ^{13}C NMR (100 MHz, 298 K) spectrum of PS **4** in d_6-DMSO.

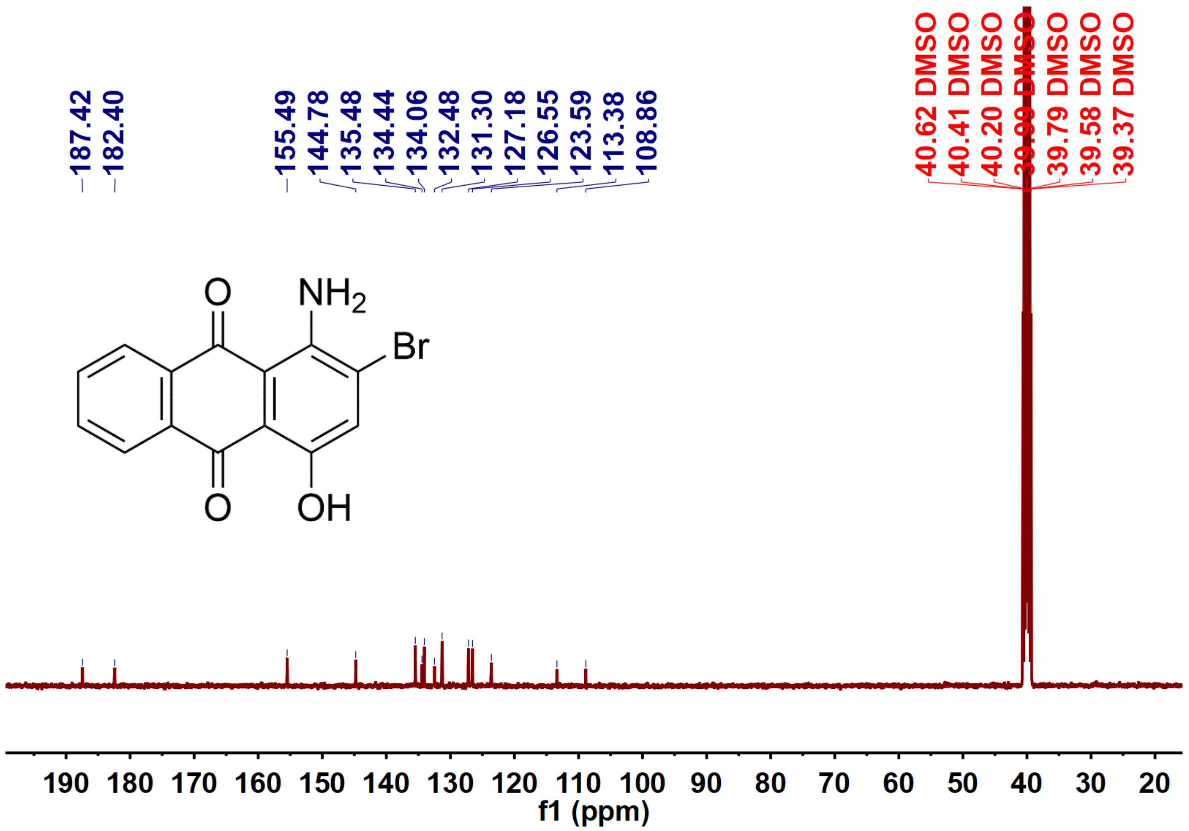

Supplementary Figure 63. ^{13}C NMR spectrum. ^{13}C NMR (100 MHz, 298 K) spectrum of PS **5** in d_6-DMSO.

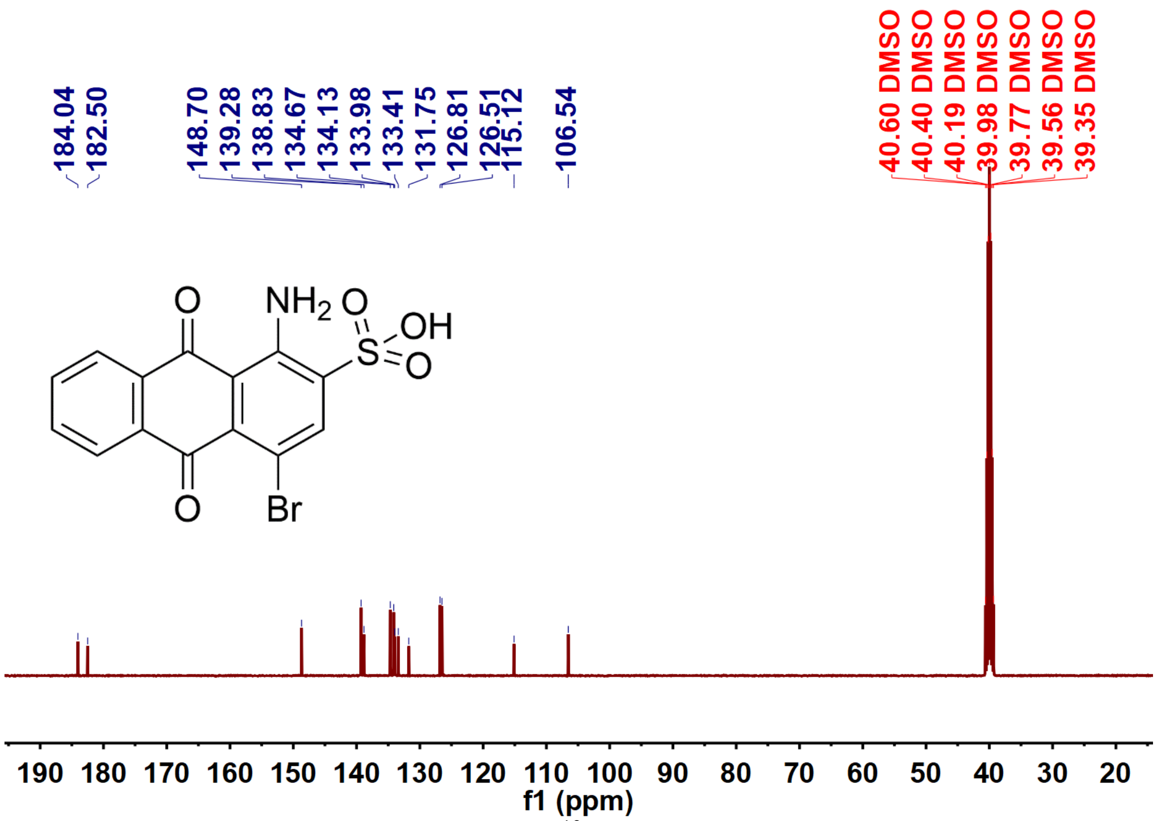

Supplementary Figure 64. ^{13}C NMR spectrum. ^{13}C NMR (100 MHz, 298 K) spectrum of PS **6** in d_6-DMSO.

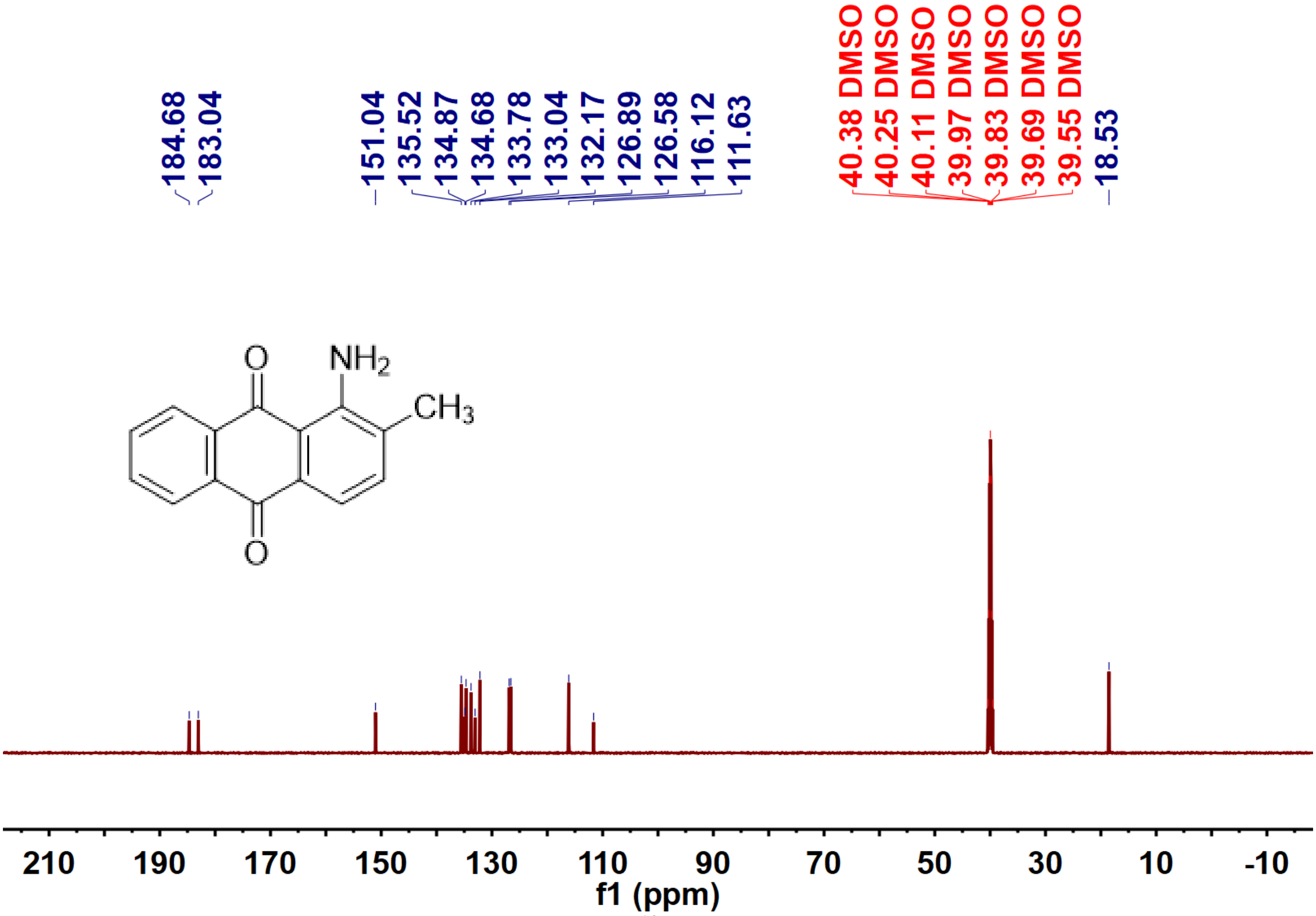

Supplementary Figure 65. ^{13}C NMR spectrum. ^{13}C NMR (100 MHz, 298 K) spectrum of 1-amino-2-methylanthraquinone in d_6-DMSO.

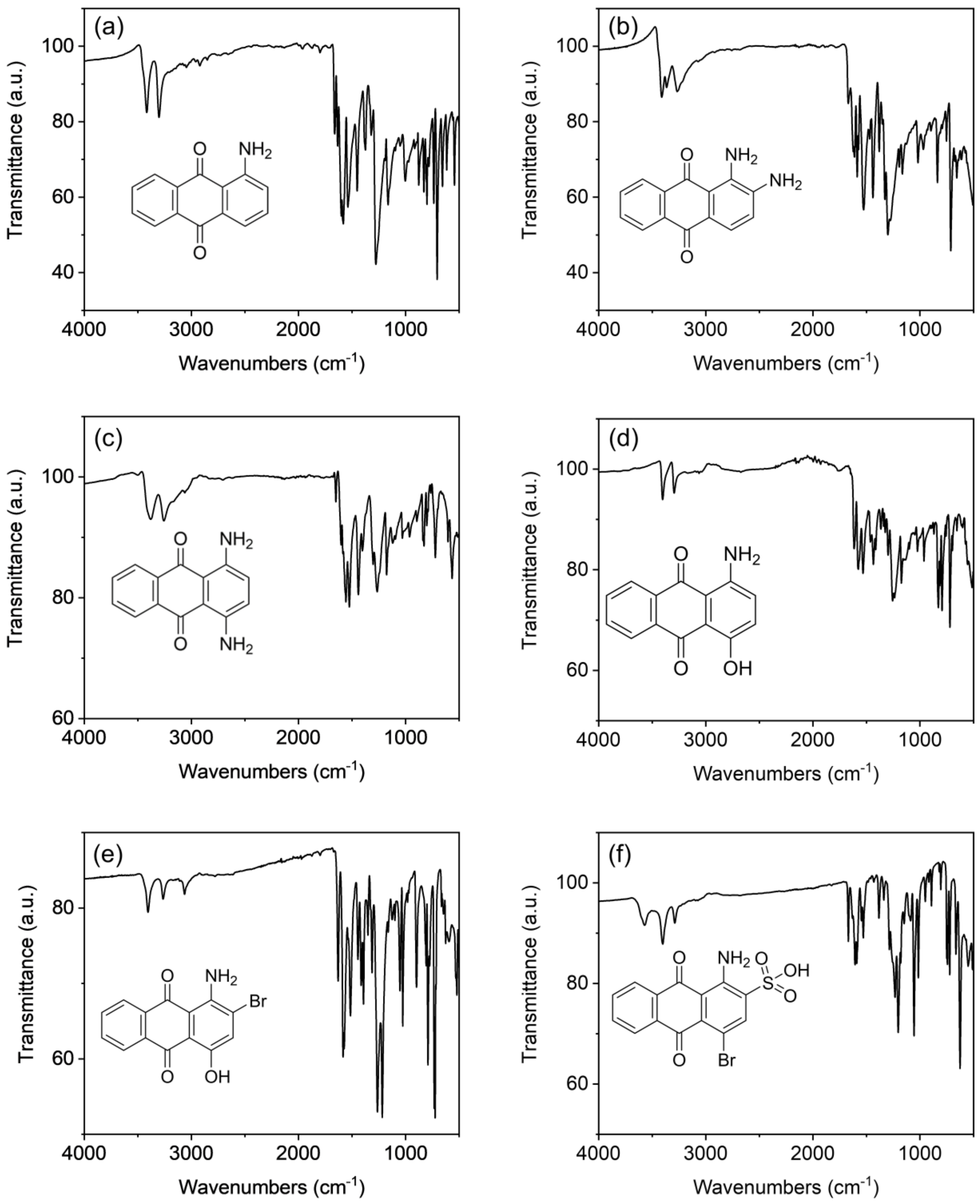

Supplementary Figure 66. FT-IR spectra. FT-IR spectra of (a) **1**; (b) **2**; (c) **3**; (d) **4**; (e) **5**; and (f) **6** at 298 K. Source data are provided as a Source Data file.

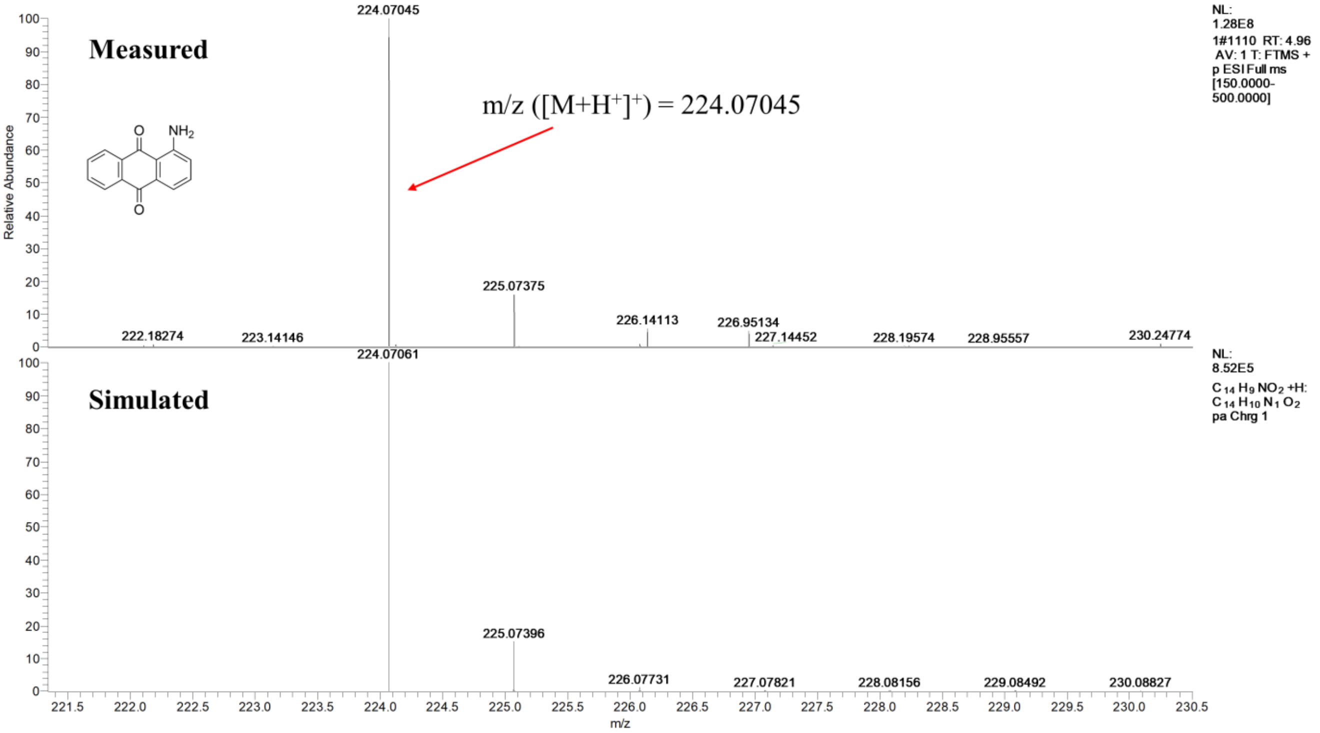

Supplementary Figure 67. HRMS spectrum. HRMS spectrum of PS **1** in CH_3OH (positive ion mode).

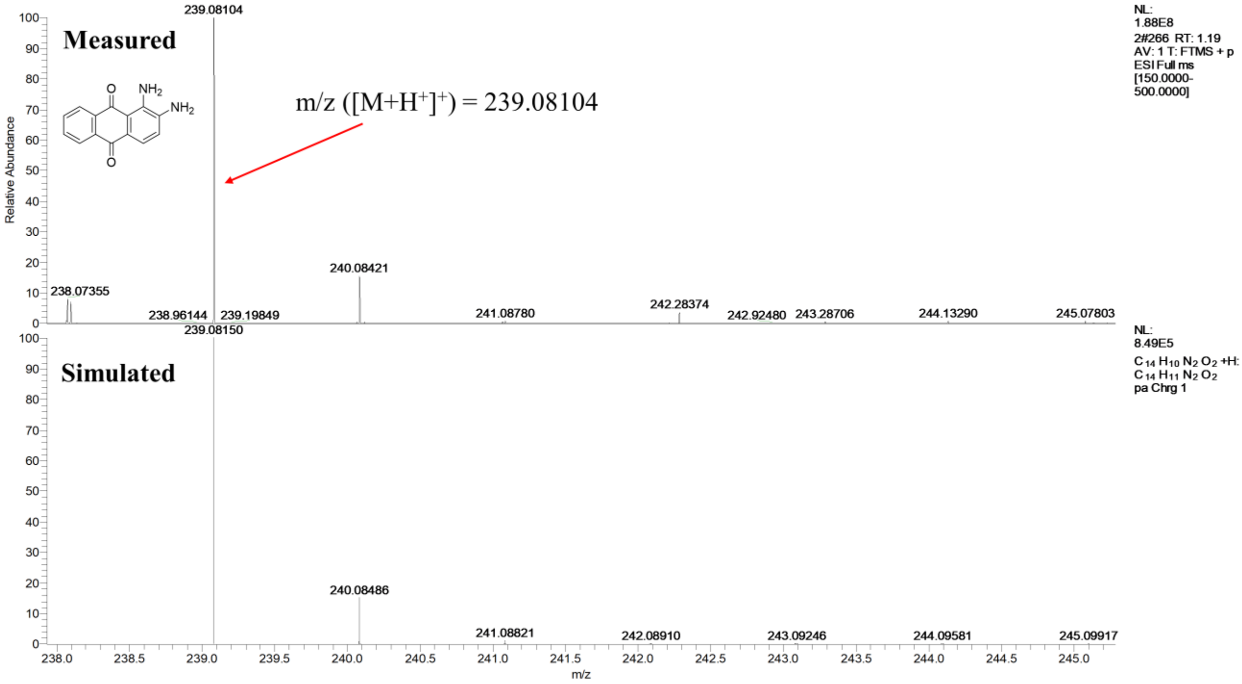

Supplementary Figure 68. HRMS spectrum. HRMS spectrum of PS **2** in CH_3OH (positive ion mode).

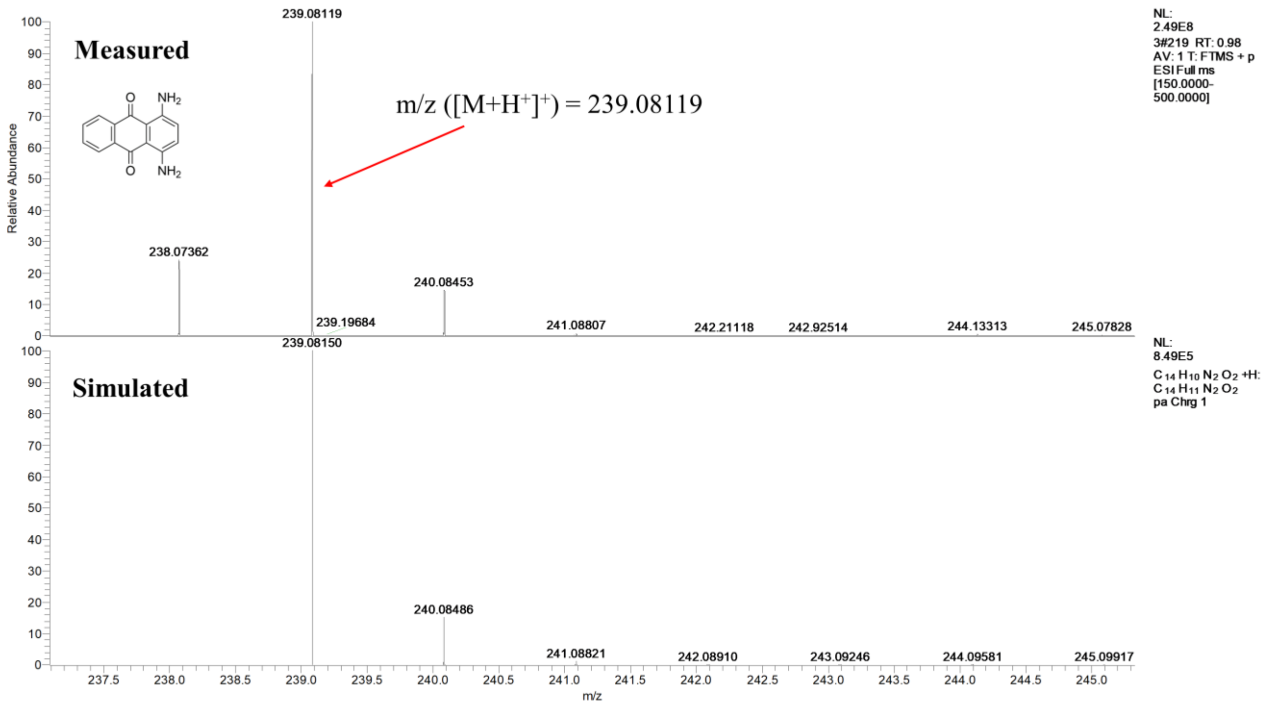

Supplementary Figure 69. HRMS spectrum. HRMS spectrum of PS **3** in CH_3OH (positive ion mode).

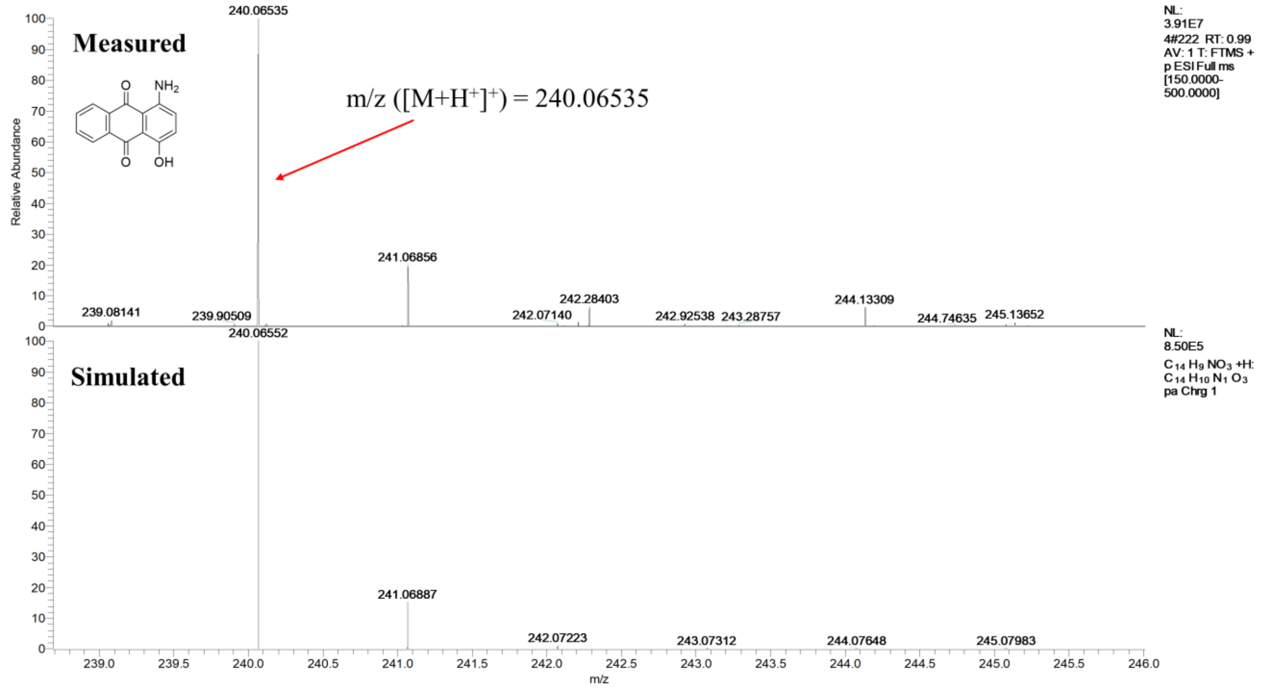

Supplementary Figure 70. HRMS spectrum. HRMS spectrum of PS **4** in CH_3OH (positive ion mode).

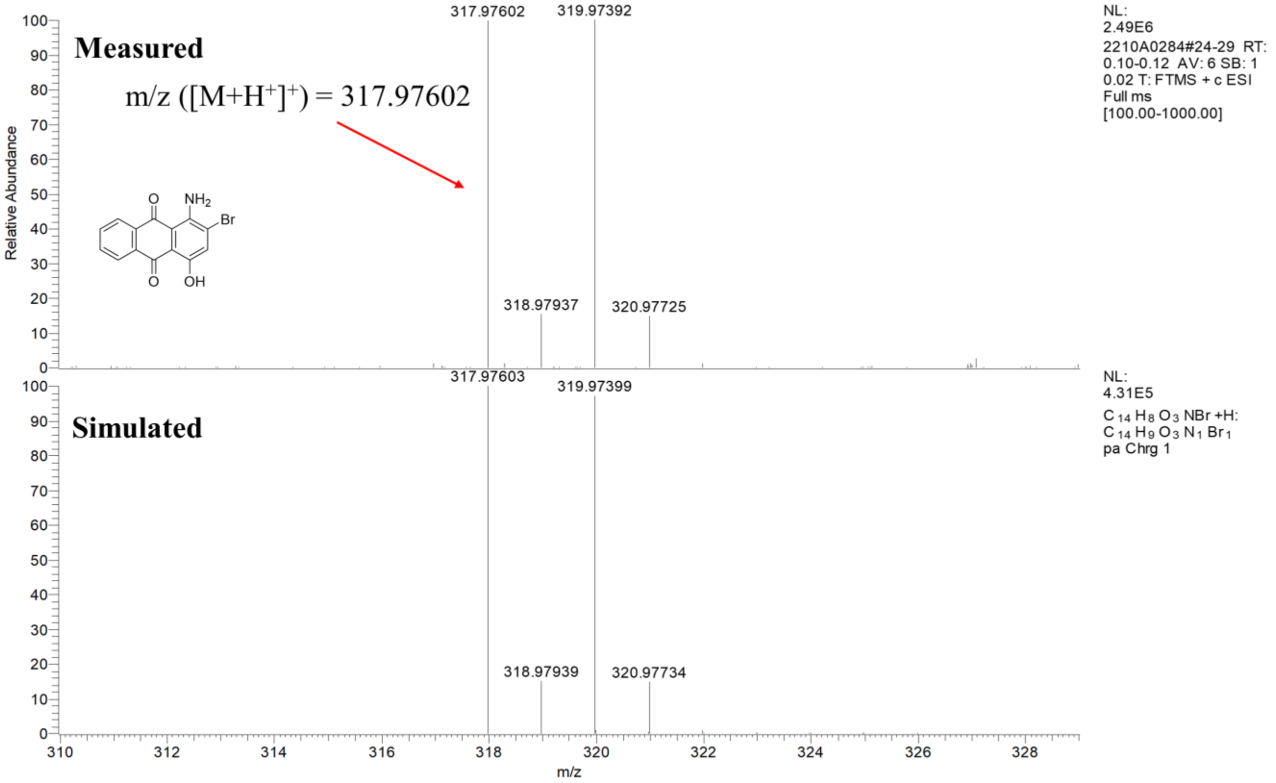

Supplementary Figure 71. HRMS spectrum. HRMS spectrum of PS **5** in CH_3OH (positive ion mode).

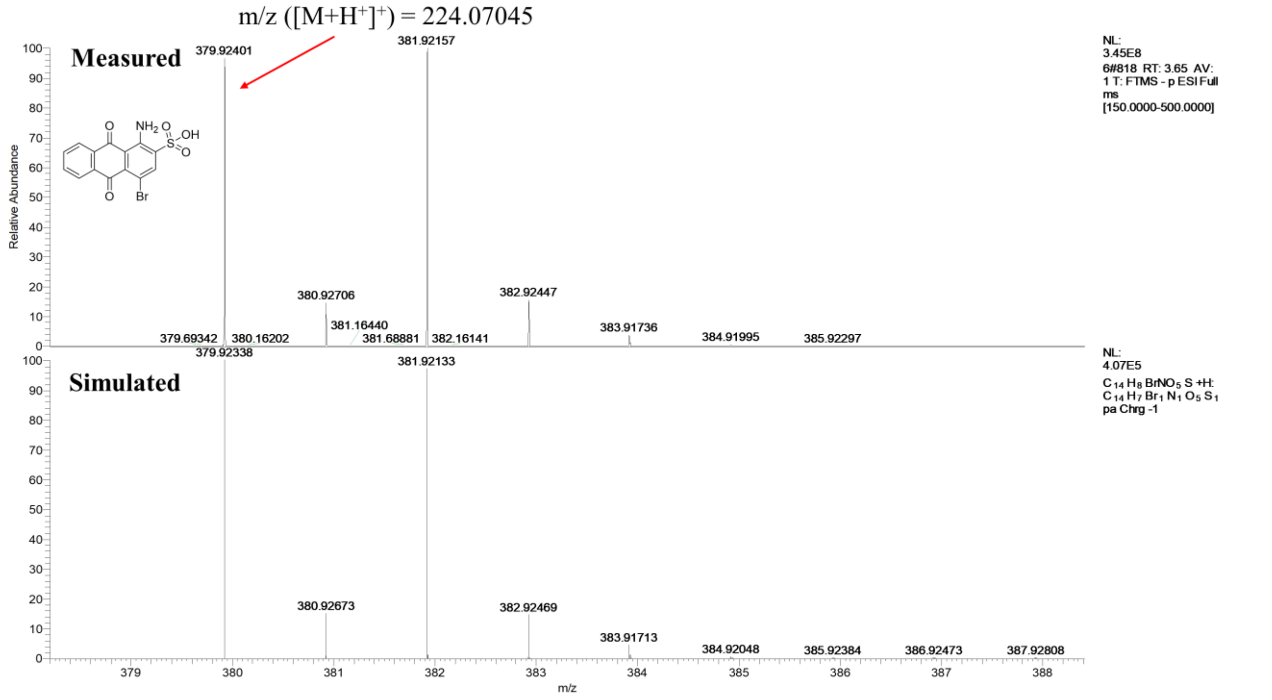

Supplementary Figure 72. HRMS spectrum. HRMS spectrum of PS **1** in CH_3OH (negative ion mode).

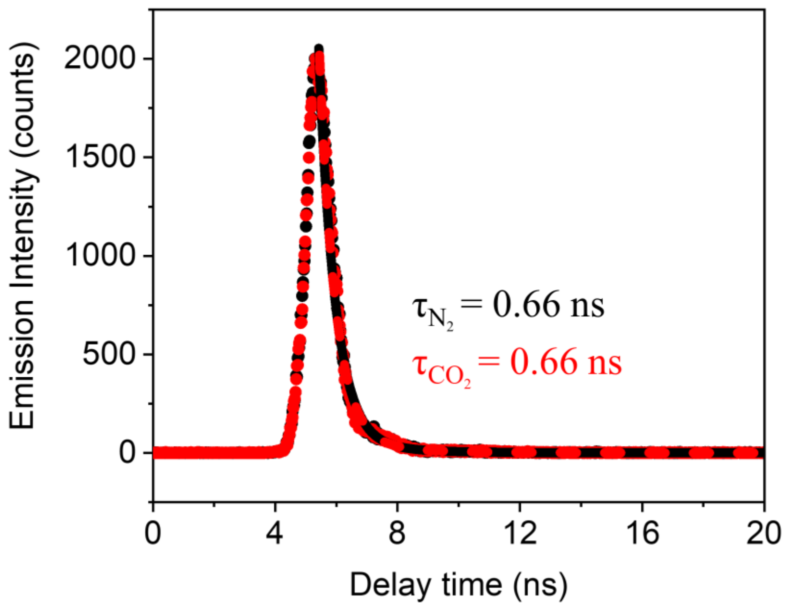

Supplementary Figure 73. Emission delay. Emission delay (λ_{exc} = 472 nm, λ_{em} = 607 nm) of 50 µM PS **6** in 2 mL DMF in a quartz cuvette (10–mm path length) at 298 K under N_2 (black) and CO_2 (red).

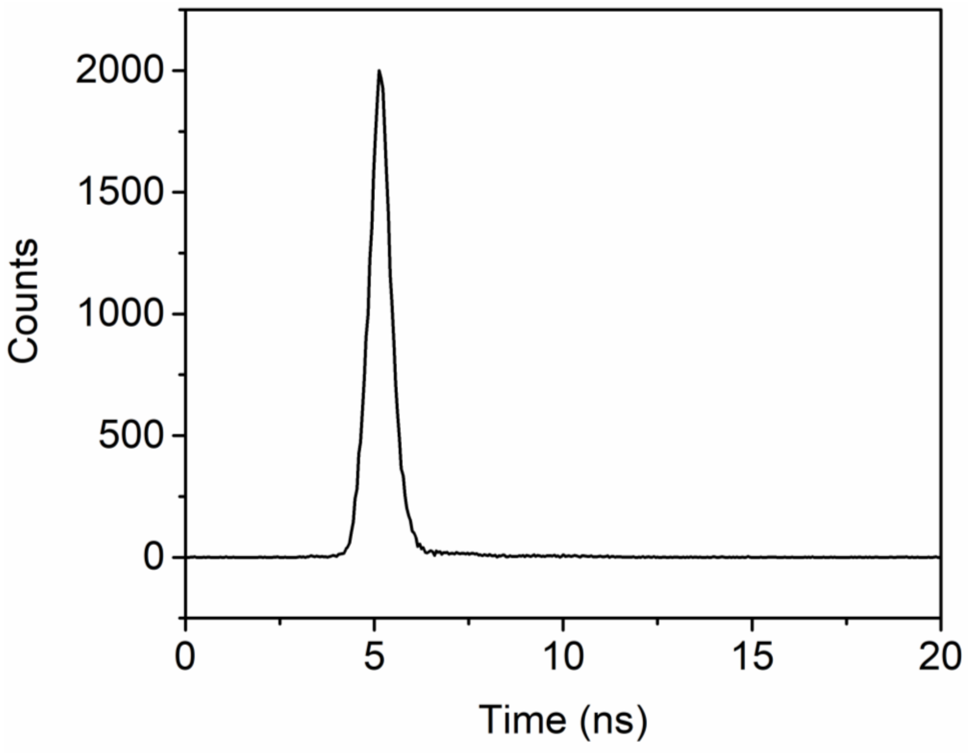

Supplementary Figure 74. Emission delay. Emission delay of the picosecond pulsed diode laser (λ = 472 nm). IRF was measured using silicon oxide (30% in H_2O) in a quartz cuvette (10–mm path length) at 298 K.

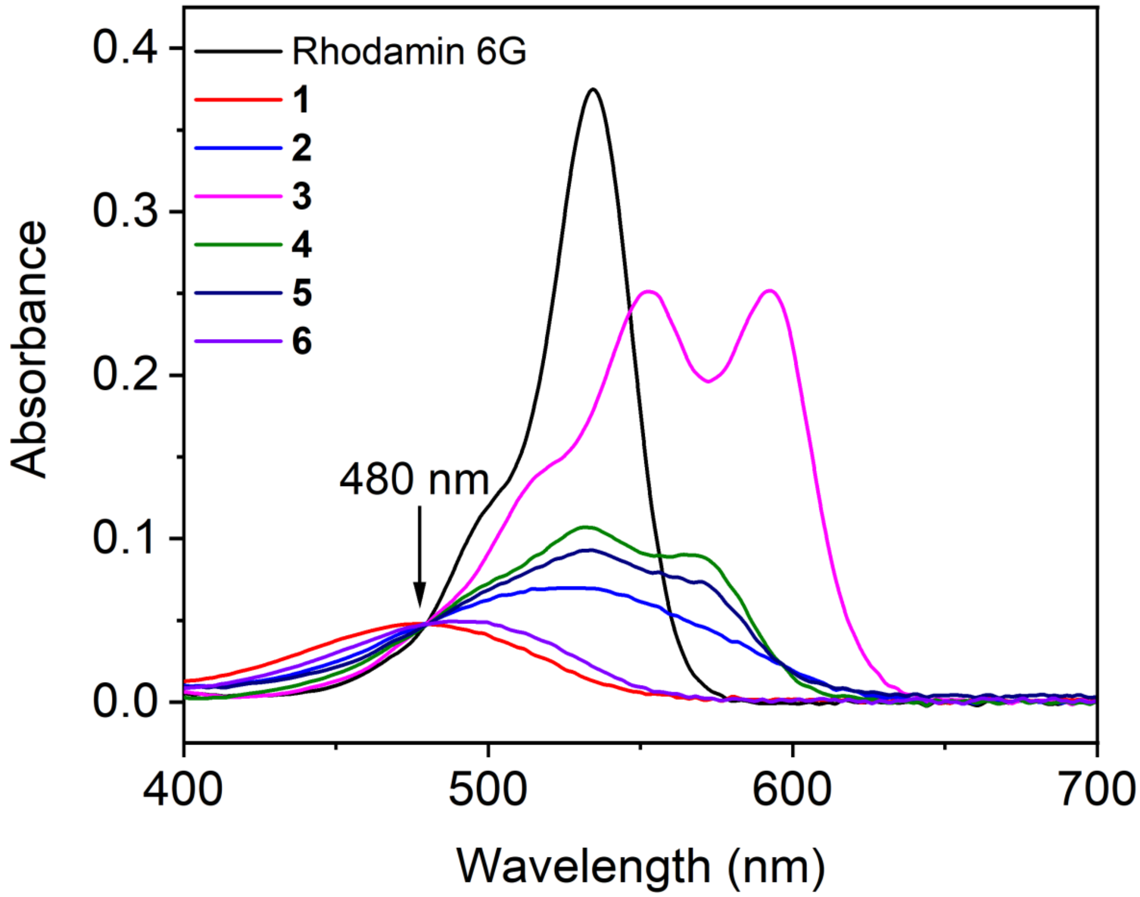

Supplementary Figure 75. UV-vis spectra. Absorption spectra of PS **1−6** and Rhodamin 6G in DMF in a quartz cuvette (10–mm path length) at 298 K. The spectra of all samples were adjusted to have the same absorbance at 480 nm for fluorescence quantum yield determination.

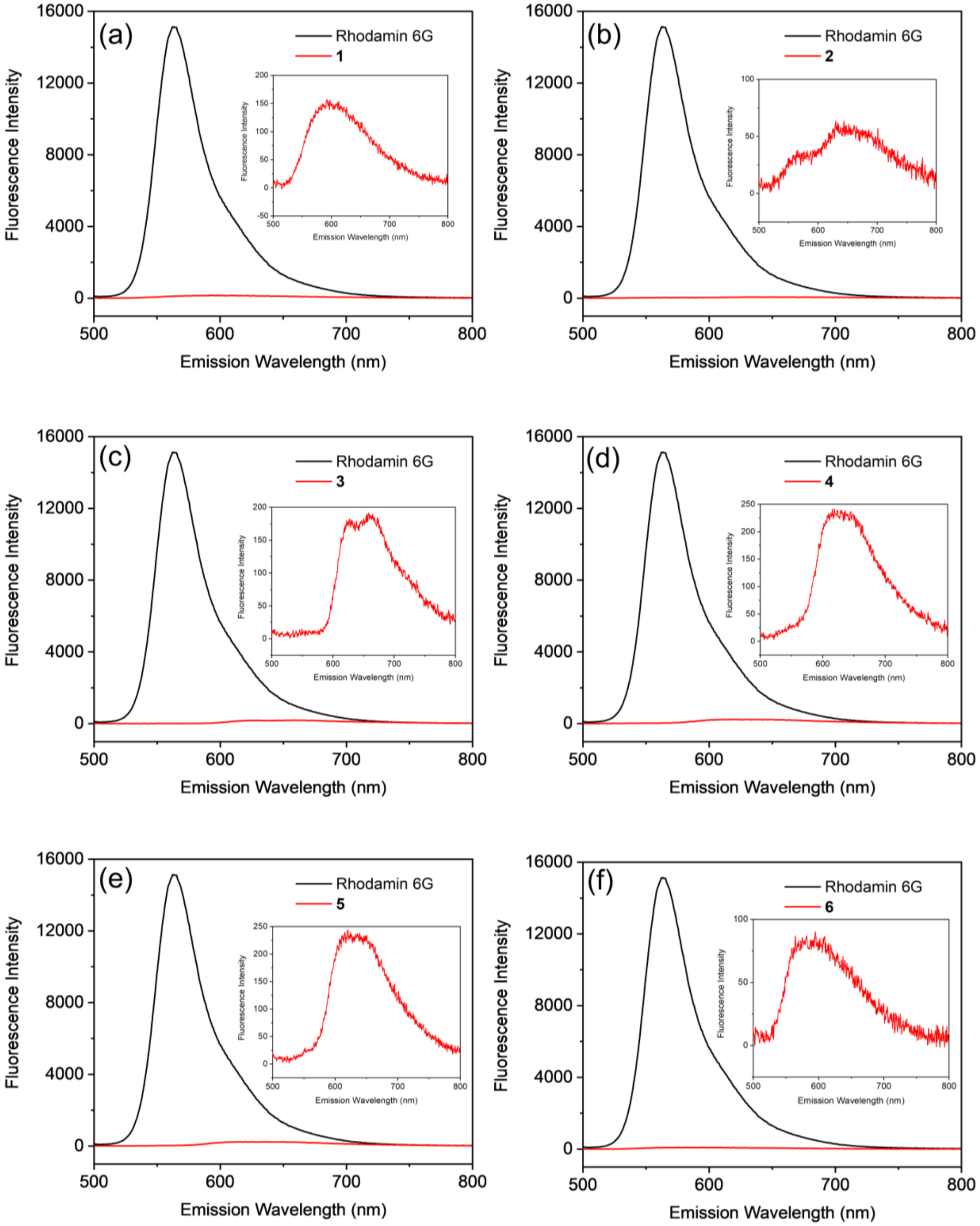

Supplementary Figure 76. Emission spectra. Emission spectra (λ_{exc} = 480 nm) of Rhodamin 6G and (a) **1**; (b) **2**; (c) **3**; (d) **4**; (e) **5**; and (f) **6** at 298 K in DMF in a quartz cuvette (10–mm path length) under N_2. The same samples were used as supplementary Figure 75. The area of each peak was integrated for fluorescence quantum yield determination.

Supplementary Table 1. Thermodynamic driving force for electron transfer of PSs 1–6.

PS	E_{red}/V	E_{ox}/V	$E_{0,0}$/eV	E^*_{red}/V	E^*_{ox}/V	ΔG / eV
**1**	−0.96, −1.59	1.32	2.30	0.71	-0.98	-0.38
**2**	−1.10, −1.70	0.82	2.09	0.39	-1.27	-0.06
**3**	−1.15, −1.64	0.73	2.03	0.39	-1.30	-0.06
**4**	−0.84, −1.44	0.81	2.11	0.67	-1.30	-0.34
**5**	−0.68, −1.19	0.91	2.10	0.91	-1.19	-0.58
**6**	−0.86, −1.30	1.40	2.26	0.96	-0.86	-0.63

$E_{0,0}$ values were obtained from the intersection of the normalized absorption and emission spectra of the fluorophore in DMF solution and converted to eV.[2] The ground state redox potentials (E_{ox} and E_{red}) were measured by electrochemical methods (CVs). The excited state redox potentials were obtained as follows: ESOP (Excited State Oxidation Potential) = $E_{ox}(PS^*)$ = E_{ox} - $E_{0,0}$; ESRP (Excited State Reduction Potential) = $E_{red}(PS^*)$ = E_{red} + $E_{0,0.}$ The thermodynamic driving force for electron transfer was calculated from Rehm-Weller equation: the difference between reduction potential of excited state of photosensitizer and oxidation potential of BIH as sacrificial reagent. ($\Delta G = E^0_{(D+/D)} - E^0_{(A/A-)} - E_{0,0} - e^2/\varepsilon d$). The last term which represents the columbic attraction energy was neglected because of small contribution to the overall energy. Therefore, the equation was simplified to $\Delta G = E_{ox}(BIH) - E^*_{red}(CuPP)$ where $E_{ox}(BIH)$ was + 0.33 V (vs SCE). Potentials are given versus SCE.

Supplementary Table 2. Data for photocatalytic CO_2 reduction.

PS	TON_{Fe}[a]	Yield rate of CO[a] (μmol/h)	TON_{PS}[b]	Yield rate of CO[b] (μmol/h)
**1**	2395 ± 228	0.15 ± 0.01	2011 ± 257	0.70 ± 0.09
**2**	2738 ± 190	0.17 ± 0.01	482 ± 76	0.17 ± 0.03
**3**	3551 ± 501	0.22 ± 0.03	1523 ± 126	0.53 ± 0.04
**4**	8360 ± 449	0.52 ± 0.03	2849 ± 161	0.99 ± 0.06
**5**	21616 ± 2351	1.35 ± 0.15	6012 ± 606	2.13 ± 0.14
**6**	907 ± 154	0.06 ± 0.01	1183 ± 78	0.41 ± 0.03

[a] 60 mM BIH, 0.6 μM FeTDHPP, and 20 μM PS, λ > 400 nm, TON_{Fe} and yield rate of CO calculated in 48 h. [b] 60 mM BIH, 20 μM FeTDHPP and 5 μM PS, λ > 400 nm, TON_{PS} and yield rate of CO calculated in 72 h. Error bars denote standard deviations, based on at least three separated runs.

Supplementary Table 3. Data for photocatalytic CO_2 reduction. Systems containing the same concentration of **5** and FeTDHPP with 60 mM BIH in 5.0 mL CO_2-saturated DMF under irradiaton with white LEDs (λ > 400 nm, 100 mW/cm^2) at 298 K. The error denotes standard deviation, based on 3 separated runs.

[PS]/[FeTDHPP] (μM)	CO (μmol)	TON	Sel_{CO}(%)
0.5	7.9 ± 0.6	3174 ± 245	99.1
1	18.0 ± 1.3	3587 ± 260	99.6
2	38.2 ± 1.4	3817 ± 136	99.9
5	124.4 ± 8.2	4978 ± 326	99.9
10	201.4	4028	99.9

Supplementary Table 4. The performance of photocatalytic CO_2 reduction to CO with organic photosensitizers in noble-metal-free systems in the literature.

PS	Catalyst	Electron donor	Solvent	TON_{CO} by Cat.	TON_{CO} by PS	Sel_{CO} (%)	Light source	Ref
**5** (20 µM)	FeTDHPP (0.6 µM)	BIH (60 mM)	DMF	21616	646	＞99.9	visible light irradiation (λ > 400 nm)	This work
**5** (5 µM)	FeTDHPP (20 µM)	BIH (60 mM)	DMF	1539	6156	> 99.9	visible light irradiation (λ > 400 nm)	This work
**5** (5 µM)	FeTDHPP (5 µM)	BIH (60 mM)	DMF	5258	5258	> 99.9	visible light irradiation (λ > 400 nm)	This work
Purpurin (0.2 mM)	Fe-*p*-TMA (2 µM)	TEA (50 mM)	MeCN/H_2O (1:9, v/v)	60	0.6	95.0	visible light irradiation (λ > 420 nm)	3
Purpurin (0.4 mM)	Fe-*p*-TMA (2 µM)	TEA (50 mM)	MeCN/H_2O (1:9, v/v)	71	0.355	95.0	visible light irradiation (λ > 420 nm)	3
Purpurin (2 mM)	$[Co(qpy)(H_2O)_2]^{2+}$ (5 µM)	BIH (100 mM)	DMF	790	1.975	90.0	blue LED (λ = 460 nm)	4
Purpurin (0.02 mM)	$[Fe(qpy)(H_2O)_2]^{2+}$ (5 µM)	BIH (100 mM)	DMF	1365	341.3	92.0	blue LED (λ = 460 nm)	4
Purpurin (0.02 mM)	$[Fe(qpy)(H_2O)_2]^{2+}$ (50 µM)	BIH (100 mM)	DMF	520	1300	97.0	blue LED (λ = 460 nm)	4
Purpurin (0.05 mM)	$[Fe(dqtpy)(H_2O)]^{2+}$ (50 µM)	BIH (100 mM)	DMF	544	544	99.3	blue LED (λ = 460 nm)	5
4CzIPN (0.05 mM)	FeTotpy (10 µM)	TEA (280 mM)	DMF/H_2O (3:2, v/v)	2250	450	99.3	visible light irradiation (λ = 420–650 nm)	6
4CzIPN (0.1 mM)	$Fe(Ntpy)_2$ (10 µM)	TEA (280mM)	DMF/H_2O (3:2, v/v)	6320	632	99.4	visible light irradiation (λ = 420–650 nm)	7
4CzIPN (0.05 mM)	Fe_6L_6 (L=Phdtpy) (4 µM)	TEA (280mM)	DMF/H_2O (3:2, v/v)	2493 (per Fe)	1196	99.6	visible light irradiation (λ = 420–650 nm)	8
9CNA (0.2 mM)	FeTDHPP (2 µM)	TEA (360mM)	MeCN	60	0.6	100.0	Xe lamp (λ > 400 nm)	9
3,7-di(4-biphenyl)-1-naphthalene-10-phenoxazine (1 mM)	Fe-p-TMA (10 µM)	TEA	DMF	140	1.4	73	visible light irradiation (λ > 435 nm)	10
phenazine (5mM)	$[Co(cyclam)(Cl)_2]^{+}$ (10mM)	TEA	TEA-MeOH-MeCN (1:1:2 v/v/v)	0.34	0.68	7.1	UV irradiation (λ > 290 nm)	11
2-ethylphenazine (5 mM)	$[Co(cyclam)(Cl)_2]^{+}$ (10mM)	TEA	TEA-MeOH-MeCN (1:1:2 v/v/v)	0.32	0.64	5.4	UV irradiation (λ > 290 nm)	11
p-terphenyl (3 mM)	CoTPP (0.05 mM)	TEA	MeCN 5% TEA	62	1.03	66	Xe lamp (λ < 300 nm)	12
p-terphenyl (3 mM)	FeTPP (0.05 mM)	TEA	MeCN 5% TEA	42	0.7	38	Xe lamp (λ < 300 nm)	12

Supplementary Table 4 (continued). The performance of photocatalytic CO_2 reduction to CO with organic photosensitizers in noble-metal-free systems in the literature.

PS	Catalyst	Electron donor	Solvent	TON_{CO} by Cat.	TON_{CO} by PS	Sel_{CO} (%)	Light source	Ref
p-terphenyl (2 mM)	$[Co(cyclam)]^{2+}$ (1.7 mM)	TEA	TEA-MeOH-MeCN (1:1:4 v/v/v)	4.5	3.83	58	UV irradiation (λ > 290 nm)	13
p-terphenyl (2 mM)	$[Co(MC\text{-}1)]^{2+}$ (1.7 mM)	TEA	TEA-MeOH-MeCN (1:1:4 v/v/v)	5.5	4.68	54	UV irradiation (λ > 290 nm)	13
p-terphenyl (2 mM)	$[Co(MC\text{-}2)]^{2+}$ (1.7 mM)	TEA	TEA-MeOH-MeCN (1:1:4 v/v/v)	5.3	4.51	52	UV irradiation (λ > 290 nm)	13
p-terphenyl (2 mM)	$[Co(MC\text{-}3)]^{2+}$ (1.7 mM)	TEA	TEA-MeOH-MeCN (1:1:4 v/v/v)	4.9	4.17	53	UV irradiation (λ > 290 nm)	13
p-terphenyl (2 mM)	$[Co(MC\text{-}4)]^{2+}$ (1.7 mM)	TEA	TEA-MeOH-MeCN (1:1:4 v/v/v)	2.0	1.70	53	UV irradiation (λ > 290 nm)	13
p-terphenyl (2 mM)	$[Co(MC\text{-}5)]^{2+}$ (1.7 mM)	TEA	TEA-MeOH-MeCN (1:1:4 v/v/v)	1.0	0.85	43	UV irradiation (λ > 290 nm)	13
p-terphenyl (2 mM)	$[Co(MC\text{-}6)]^{2+}$ (1.7 mM)	TEA	TEA-MeOH-MeCN (1:1:4 v/v/v)	27	22.95	81	UV irradiation (λ > 290 nm)	13
p-terphenyl (0.05 mM)	$[Co(cyclam)]^{2+}$ (1.7 mM)	TEOA	TEOA-MeOH-MeCN (1:1:4 v/v/v)	9.6	326.40	55	UV irradiation (λ > 290 nm)	14

Supplementary Table 5. The performance of photocatalytic CO_2 reduction with PS **5** in our study.

Photosensitizer	Catalyst	Electron donor	TON_{CO} by Cat.	TON_{CO} By PS	Sel_{CO}(%)
**5** (20 μM)	FeTDHPP (0.5 μM)	BIH (60 mM)	19158	479	＞99.9
**5** (20 μM)	FeTDHPP (0.6 μM)	BIH (60 mM)	21616	646	＞99.9
**5** (20 μM)	FeTDHPP (1 μM)	BIH (60 mM)	17020	851	＞99.9
**5** (20 μM)	FeTDHPP (2 μM)	BIH (60 mM)	11250	1125	＞99.9
**5** (20 μM)	FeTDHPP (10 μM)	BIH (60 mM)	3942	1971	＞99.9
**5** (20 μM)	FeTDHPP (20 μM)	BIH (60 mM)	2134	2134	＞99.9
**5** (20 μM)	FeTDHPP (50 μM)	BIH (60 mM)	891	2227	＞99.9
**5** (15 μM)	FeTDHPP (2 μM)	BIH (60 mM)	8772	1170	＞99.9
**5** (10 μM)	FeTDHPP (2 μM)	BIH (60 mM)	5593	1119	＞99.9
**5** (10 μM)	FeTDHPP (5 μM)	BIH (60 mM)	4128	2064	＞99.9
**5** (10 μM)	FeTDHPP (10 μM)	BIH (60 mM)	4028	4028	＞99.9
**5** (10 μM)	FeTDHPP (20 μM)	BIH (60 mM)	2325	4649	> 99.9
**5** (5 μM)	FeTDHPP (2 μM)	BIH (60 mM)	8780	3512	> 99.9
**5** (5 μM)	FeTDHPP (5 μM)	BIH (60 mM)	5258	5258	> 99.9
**5** (5 μM)	FeTDHPP (10 μM)	BIH (60 mM)	2576	5152	> 99.9
**5** (5 μM)	FeTDHPP (20 μM)	BIH (60 mM)	1539	6156	> 99.9

Supplementary References

1. Klaper, M., Wessig, P. & Linker, T. Base catalysed decomposition of anthracene endoperoxide. *Chem. Commun.* **52**, 1210-1213, (2016).
2. Mejía, E. *et al.* A noble-metal-free system for photocatalytic hydrogen production from water. *Chem. Eur. J.* **19**, 15972-15978, (2013).
3. Rao, H., Bonin, J. & Robert, M. Visible-light homogeneous photocatalytic conversion of CO_2 into CO in aqueous solutions with an iron catalyst. *ChemSusChem* **10**, 4447-4450, (2017).
4. Guo, Z. *et al.* Highly efficient and selective photocatalytic CO_2 reduction by iron and cobalt quaterpyridine complexes. *J. Am. Chem. Soc.* **138**, 9413-9416, (2016).
5. Chen, L. *et al.* A molecular noble metal-free system for efficient visible light-driven reduction of CO_2 to CO. *Dalton Trans.* **48**, 9596-9602, (2019).
6. Wang, Y., Gao, X.-W., Li, J. & Chao, D. Merging an organic TADF photosensitizer and a simple terpyridine–Fe(III) complex for photocatalytic CO_2 reduction. *Chem. Commun.* **56**, 12170-12173, (2020).
7. Wang, Y., Liu, T., Chen, L. & Chao, D. Water-assisted highly efficient photocatalytic reduction of CO_2 to CO with noble metal-free bis(terpyridine)iron(II) complexes and an organic photosensitizer. *Inorg. Chem.* **60**, 5590-5597, (2021).
8. Wang, Y., Chen, L., Liu, T. & Chao, D. Coordination-driven discrete metallo-supramolecular assembly for rapid and selective photochemical CO_2 reduction in aqueous solution. *Dalton Trans.* **50**, 6273-6280, (2021).
9. Bonin, J., Robert, M. & Routier, M. Selective and efficient photocatalytic CO_2 reduction to CO using visible light and an Iron-based homogeneous catalyst. *J. Am. Chem. Soc.* **136**, 16768-16771, (2014).
10. Rao, H., Lim, C.-H., Bonin, J., Miyake, G. M. & Robert, M. Visible-light-driven conversion of CO_2 to CH_4 with an organic sensitizer and an iron porphyrin catalyst. *J. Am. Chem. Soc.* **140**, 17830-17834, (2018).
11. Ogata, T. *et al.* Phenazine-photosensitized reduction of CO_2 mediated by a cobalt-cyclam complex through electron and hydrogen transfer. *J. Phys. Chem.* **99**, 11916-11922, (1995).
12. Dhanasekaran, T., Grodkowski, J., Neta, P., Hambright, P. & Fujita, E. p-terphenyl-sensitized photoreduction of CO_2 with cobalt and iron porphyrins. Interaction between CO and reduced metalloporphyrins. *J. Phys. Chem. A* **103**, 7742-7748, (1999).
13. Matsuoka, S. *et al.* Efficient and selective electron mediation of cobalt complexes with cyclam and related macrocycles in the p-terphenyl-catalyzed photoreduction of carbon dioxide. *J. Am. Chem. Soc.* **115**, 601-609, (1993).
14. Matsuoka, S., Yamamoto, K., Pac, C. & Yanagida, S. Enhanced p-terphenyl-catalyzed photoreduction of CO_2 to CO through the mediation of Co(III)–cyclam complex. *Chem. Lett.* **20**, 2099-2100, (1991).
